# Relationships between species richness and ecosystem services in Amazonian forests strongly influenced by biogeographical strata and forest types

**DOI:** 10.1038/s41598-022-09786-6

**Published:** 2022-04-08

**Authors:** Gijs Steur, Hans ter Steege, René W. Verburg, Daniel Sabatier, Jean-François Molino, Olaf S. Bánki, Hernan Castellanos, Juliana Stropp, Émile Fonty, Sofie Ruysschaert, David Galbraith, Michelle Kalamandeen, Tinde R. van Andel, Roel Brienen, Oliver L. Phillips, Kenneth J. Feeley, John Terborgh, Pita A. Verweij

**Affiliations:** 1 Copernicus Institute of Sustainable Development, Utrecht University, Utrecht, The Netherlands; 2 Utrecht University Botanic Gardens, Utrecht, The Netherlands; 3 Naturalis Biodiversity Center, Leiden, The Netherlands; 4 Systems Ecology, Vrije Universiteit Amsterdam, Amsterdam, The Netherlands; 5 AMAP, IRD, Cirad, CNRS, INRAE, Université de Montpellier, Montpellier, France; 6 Universidad Nacional Experimental de Guayana, Puerto Ordaz, Bolivar Venezuela; 7 Museo Nacional de Ciencias Naturales, Madrid, Spain; 8 Direction Régionale de La Guyane, ONF, Cayenne, French Guiana; 9 Ghent University, Ghent, Belgium; 10 University of Leeds, Leeds, UK; 11 University of Cambridge, Cambridge, UK; 12 Living with Lakes Centre, Laurentian University, Sudbury, ON Canada; 13 Biosystematics group, Wageningen University, Wageningen, The Netherlands; 14 University of Miami, Coral Gables, USA; 15 Fairchild Tropical Botanic Garden, Coral Gables, FL USA; 16 University of Florida, Gainesville, USA; 17 Centre for Tropical Environmental and Sustainability Science and College of Science and Engineering, James Cook University, Cairns, QLD Australia

**Keywords:** Plant ecology, Ecosystem services

## Abstract

Despite increasing attention for relationships between species richness and ecosystem services, for tropical forests such relationships are still under discussion. Contradicting relationships have been reported concerning carbon stock, while little is known about relationships concerning timber stock and the abundance of non-timber forest product producing plant species (NTFP abundance). Using 151 1-ha plots, we related tree and arborescent palm species richness to carbon stock, timber stock and NTFP abundance across the Guiana Shield, and using 283 1-ha plots, to carbon stock across all of Amazonia. We analysed how environmental heterogeneity influenced these relationships, assessing differences across and within multiple forest types, biogeographic regions and subregions. Species richness showed significant relationships with all three ecosystem services, but relationships differed between forest types and among biogeographical strata. We found that species richness was positively associated to carbon stock in all biogeographical strata. This association became obscured by variation across biogeographical regions at the scale of Amazonia, resembling a Simpson’s paradox. By contrast, species richness was weakly or not significantly related to timber stock and NTFP abundance, suggesting that species richness is not a good predictor for these ecosystem services. Our findings illustrate the importance of environmental stratification in analysing biodiversity-ecosystem services relationships.

## Introduction

Despite considerable scientific attention for the relationships between biodiversity and ecosystem services, the extent to which such relationships exist in tropical forests remains unclear. Tropical forests are one of the most species-rich ecosystems on Earth^1^, store an estimated 54% of the global aboveground carbon stock^2^ and provide valuable timber^3^ and non-timber forest products^4^, such as food, medicines and cultural ornaments. However, tropical forests are increasingly being degraded or lost^5^, threatening their biodiversity and their goods and services that benefit human wellbeing. Under the expectation that ecosystem services are generally positively linked to biodiversity, there is increasing attention for ecosystem services as a rationale to help conserve tropical forest biodiversity^6–8^. For example, contemporary conservation approaches, such as UN REDD+ , focus on tropical forests with high carbon stocks, assuming that such forests will be biodiverse as well^9^. However, it is uncertain to what extent the number of tree and arborescent palm species, hereafter referred to as ‘woody species richness’, is related to carbon storage, timber provisioning and non-timber forest product (NTFP) provisioning in tropical forests, obscuring the extent to which conservation of ecosystem services can help protect tropical forest biodiversity.

In tropical forests, woody species are the main components of the aboveground plant biomass, and can therefore, be expected to be related to biomass-based ecosystem services, such as carbon storage, timber provisioning, and the supply of non-timber forest products (‘NTFPs’). Several hypotheses have been proposed to explain how plant diversity can enhance biomass and therefore the relationship between woody species richness and aboveground biomass in tropical forests would be expected to be positive. According to the ‘niche complementary’ hypothesis^10^, species-rich communities have a higher variation in species traits, and thus, could better utilise limited available resources. This would result in increased productivity, which can in turn, result in higher aboveground biomass^11–14^. In addition, according to the ‘insurance’ hypothesis^15^, a higher variation in species traits allows a community to be more resilient against environmental fluctuations, maintain a high productivity across time and thus, enable a higher aboveground biomass^11,14^. Last, according to the ‘selection effect’ hypothesis^10^, species-rich communities have a higher chance of including species with higher biomass, resulting in higher sampled average aboveground biomass^11–13^.

However, although there has been considerable support for positive species-biomass relationships in grasslands and non-tropical forests and plantations ^16–20^, the empirical evidence for relationships between woody species richness and carbon storage, timber provisioning, and NTFP provisioning in tropical forests remains inconclusive. The review and meta-analysis of such relationships across tropical forests by Steur et al*.*^6^ identified contrasting results and knowledge gaps across Amazonia, the tropical forest area comprising of the Amazon River basin and the Guiana Shield. Most studies have focused on the aboveground carbon stock, hereafter referred to as ‘carbon stock’. In recent studies, both positive and non-significant relationships have been reported for woody species richness and carbon stock^11,13,21–23^. By contrast, little to no attention has been given to the relationship of woody species richness with commercially relevant timber stock, hereafter referred to as ‘timber stock’, or its relationship with the abundance of tree and arborescent palms that produce commercially relevant NTFPs, hereafter referred to as ‘NTFP abundance’^6^. As for timber and NTFP provisioning, only a subset of the available plant species will be relevant, while no a-priori prediction can be made for the relationships with species richness. Although a more recent study by Steur et al*.*^24^ reported a negative relationship between woody species richness and NTFP abundance in Suriname lowland tropical forests, the extent of this relationship across other tropical forests and different spatial scales remains unclear.

To date, the contrasting results for the relationship between woody species richness and carbon stock across Amazonia have remained unexplained. Although previous studies found that plot size can moderate the ‘species-carbon relationship’^11,13,19^, contrasting results have been found for studies that use the same plot size ^6^. For example, in studies using 1-ha plots, Aldana et al*.*^21^ found a positive relationship across Colombian tropical lowland forests, while Poorter et al*.*^11^ and Sullivan et al*.*^13^ did not find a significant bivariate relationship across a wide range of Neotropical forests. Although Poorter et al*.*^11^ ultimately found a positive relationship when variation in rainfall, stem density and stem diameter was accounted for, Sullivan et al*.*^13^ did not find any such positive relationship, even when variation in multiple climatic and edaphic variables were accounted for. As a possible explanation, the meta-analysis by Steur et al*.*^6^ suggested that contrasting results on the species-carbon relationship may be due to differences in geographical extent covered by the study area. The meta-analysis showed a positive species-carbon relationship across the tropics, but the strength of this relationship decreased with increasing amount of geographical extent covered. Such a pattern can also be observed in the aforementioned studies: Aldana et al*.*^21^ found a significant positive relationship at the geographical extent of Colombia, while Poorter et al*.*^11^ and Sullivan et al*.*^13^ found no significant bivariate relationship at larger extents ranging the Neotropics.

Steur et al*.*^6^ postulated that, with increasing geographical extent, an increasing amount of environmental heterogeneity is sampled, which ultimately moderates the relationship between woody species richness and carbon stock. In Amazonia, woody species diversity and aboveground biomass vary across environmental gradients likely to be increasingly sampled when the geographical extent of the study increases. For example, significant differences in woody species fisher’s alpha and aboveground biomass have been observed across soil and forest types ^25–27^ and across biogeographical regions and subregions of Amazonia ^25,28–30^. Specifically for forest types, Aldana et al*.*^21^ found a positive species-carbon relationship for Colombian terra firme forests, but no such relationship when terra firme forests were aggregated with flooded forests. However, a systematic analysis of the influence of soil type, forest type and biogeographical strata on relationships between woody species richness and ecosystem services for Amazonian tropical forests has not been conducted.

This study aims to provide insights into the relationships between species richness and multiple ecosystem services while accounting for the influence of environmental stratification at different spatial scales with respect to the tropical forests of Amazonia. For our analyses, we use two datasets of collectively 283 1-ha Amazonian lowland tropical forest plots: one spanning the Guiana Shield region composed of primary plot data and the other spanning all of Amazonia that was created by combining the Guiana Shield data with secondary published plot data. With the primary data from the Guiana Shield, we calculated woody species richness, carbon stock, timber stock and NTFP abundance, and tested their relationships across and within two main forest types and four biogeographical subregions. In addition, with the secondary data, we also tested the species-carbon relationship across and within six biogeographical regions of Amazonia. Unfortunately, local commercial demand for timber stock and NTFP abundance could only be adequately determined for the Guiana Shield region and was not available for the scale of Amazonia.

## Results

### Relationships across the Guiana Shield

**Figure 1:**
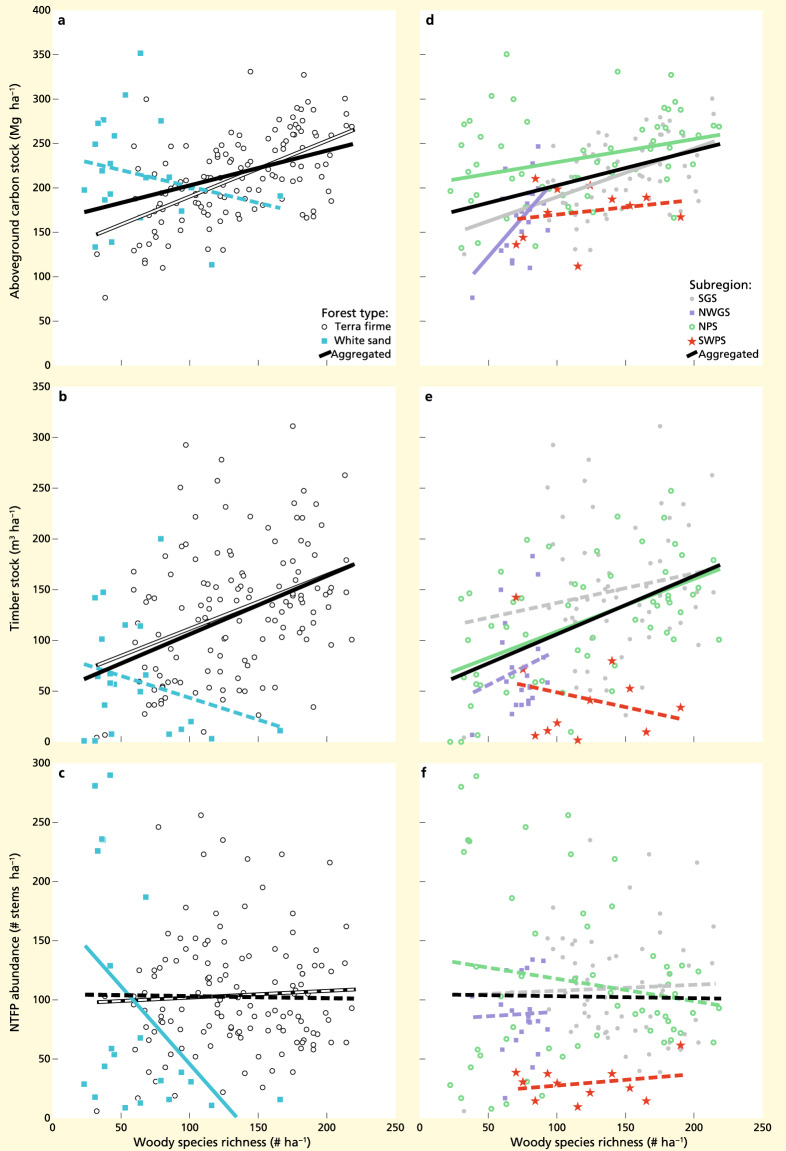
Visualisation of linear bivariate relationships between species richness and carbon stock, timber stock, and non-timber forest products (‘NTFP’) abundance, across and within two forest types and four biogeographical subregions of the Guiana Shield. Showing relationships between species richness and carbon stock (panels **a** and **d**), between species richness and timber stock (panels** b** and** e**), and between species richness and NTFP abundance (panels **c** and **f**). Relationships across all forest types and subregions indicated by black lines (n = 151), within terra firme forests by white lines (n = 130), within white sand forests by blue lines (n = 21), within the Southern Guiana Shield by gray lines (n = 63; SGS), within the north-western Guiana Shield by purple lines (n = 21; NWGS), within the northern Pleistocene sands by green lines (n = 56; NPS), and within the south-western Pleistocene sands in the upper Rio Negro region by red lines (n = 11; SWPS). Solid lines indicate significant relationships (*p* < 0.05) and dashed lines non-significant relationships (*p* ≥ 0.05). Forest plots are coloured according to forest type or subregion. Model details are included in Tables S2.4 and S2.7.

For the Guiana Shield, species richness showed a positive relationship with carbon stock and timber stock across all biogeographical subregions and forest types (explaining 15.8 and 18.2% of variation, respectively; both coefficients *p* ≤ 0.0003; Table S2.1), but relationships differed for the two forest types and four biogeographical subregions (Fig. 1). Species richness was positively related to carbon stock in three of the four subregions (all three coefficients *p* ≤ 0.0186), whereas it was positively related to timber stock only in one subregion (coefficient *p* < 0.0001). In addition, it was positively related to carbon stock and timber stock in terra firme forests, but not significantly related in white sand forests. By contrast, species richness was not significantly related to NTFP abundance across the biogeographical subregions and forest types (coefficient *p* = 0.8570; Table S2.1), only showing a significant but negative relationship with NTFP abundance in white sand forests (coefficient *p* = 0.0351).

Results showed that variation in carbon stock and timber stock was explained by a combination of species richness, biogeographical subregion and forest type, while variation in NTFP abundance was explained by biogeographical subregions only (Table 1). However, accounting for variation in biogeographical subregions and forest types did not result in significantly different relationships between species richness, carbon stock, timber stock and NTFP abundance across the Guiana Shield (Table 1 vs. Table S2.1). In all three relationships, biogeographical subregions explained a substantial part of the total variation (ranging between 14.7 and 19.3%). For carbon stock, species richness explained a similar amount of variation as when variation in forest type and biogeographical subregion was not accounted for (15.1 vs. 15.8%; Table 1 vs. Table S2.1). For timber stock, the contribution of species richness was considerably less (9.3 vs. 18.2%; Table 1 vs. Table S2.1). Last, forest type explained a small amount of variation in carbon stock and timber stock (2.4 and 6.5%, respectively; Table 1).

**Table 1:** Summary of optimized multiple linear models of carbon stock, timber stock and NTFP abundance predicted by species richness and environmental covariables across the Guiana Shield dataset (n = 151 1-ha plots).

	Relationship summary	Rel. contr. R^2^ (%)	Total R^2^ (%)
**Carbon stock**			
Subregions	Significant variable	19.3	
Species richness	Significant positive	15.1	
Forest type	Significant variable	2.4	
			36.8
**Timber stock**			
Subregions	Significant variable	18.1	
Species richness	Significant positive	9.3	
Forest type	Significant variable	6.5	
			33.9
**NTFP abundance**			
Subregions	Significant variable	14.7	
			14.7

Originally included predictors were species richness, forest type, and subregion. For each retained predictor, a summary of the relationship and the relative contribution to total model R^2^ (%) is given. NTFP abundance = abundance of species that produce non-timber forest products. Model details are included in Table S2.2.

### Relationships across Amazonia

**Figure 2:**
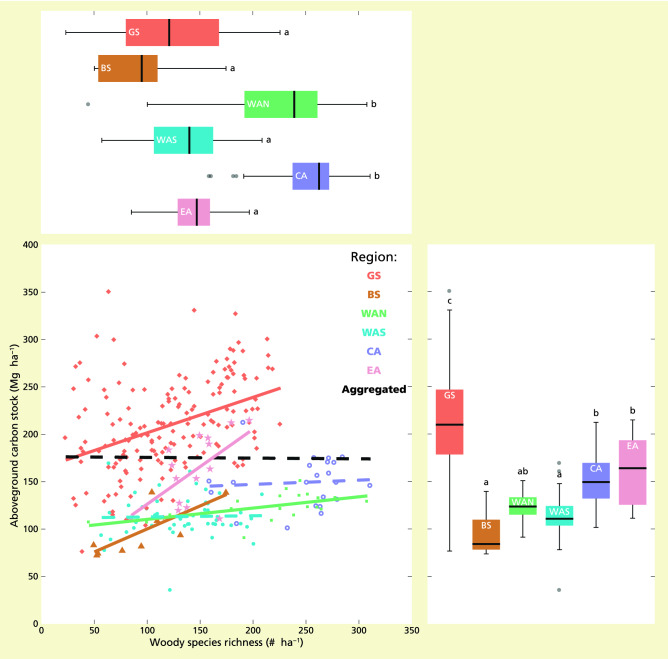
Visualisation of linear bivariate relationships between carbon stock and species richness for different biogeographical regions in the Amazonia dataset. Relationships across all biogeographical regions (Agg., n = 283): black line; for Guiana Shield (GS, n = 165): red line, Brazilian Shield (BS, n = 9): dark yellow line, north-western Amazonia (WAN, n = 21): green line, south-western Amazonia (WAS, n = 51): light blue line, central Amazonia (CA, n = 22): purple line, and eastern Amazonia (EA, n = 15): pink line. Showing boxplots for carbon stock (bottom left) and species richness (upper panel) across the regions with differences according to Tukey post-hoc tests indicated by different letters. Model details are included in Tables S2.13–S2.15.

In contrast to the positive relationship between species richness and carbon stock observed across the Guiana Shield (Table 1), across Amazonia species richness showed no significant relationship with carbon stock (slope − 0.007, *p* = 0.8950; Table S2.10). However, the relationship differed for single biogeographical regions, where the relationship was either positive, or non-significant but weakly positive (all slopes ≥ 0.013; Table S2.13; Fig. 2). When variation in carbon stock across biogeographical regions was accounted for, a positive relationship between species richness and carbon stock was found across Amazonia (slope 0.289, *p* < 0.0001; Table S2.12). By contrast, the relationship between species richness and carbon stock did not differ between forest types (Figure S2.5), and accounting for variation in carbon stock between forest types did not lead to a significant relationship (Table S2.12).

Results showed that 60.0% of variation in carbon stock was explained by species richness, biogeographical regions and forest types (Table 2). Here, variation in carbon stock was for a large part explained by variation across biogeographical regions (54.9%), while species richness and forest type had small contributions (3.4 and 1.7%, respectively).

**Table 2:** Summary of the optimized multiple linear model of carbon stock predicted by species richness and environmental covariables across the Amazonia dataset (n = 283 1-ha plots).

	Relationship summary	Rel. contr. R^2^ (%)	Total R^2^ (%)
**Carbon stock**			
Biogeographical region	Significant variable	54.9	
Species richness	Significant positive	3.4	
Forest type	Significant variable	1.7	
			60.0

Originally included predictors were species richness, forest type and biogeographical region. For each predictor, a summary of the relationship and the relative contribution to total model R^2^ (%) is given. Model details included in Table S2.11.

## Discussion

In this study we analysed how tree and arborescent palm species richness was related to aboveground carbon stock, commercially relevant timber stock, and commercially relevant NTFP abundance in tropical forests, and how these relationships were influenced by environmental stratification at different spatial scales. We found that species richness showed significant relationships with all three ecosystem services stock components, but its relationships were strongly influenced by variation across forest types and biogeographical strata. This is further explained below.

Across the Guiana Shield, species richness showed a positive relationship with carbon stock and timber, but not with NTFP abundance. Although relationships only differed in significance among the biogeographical subregions, they differed in direction between terra firme forests and white sand forests. Species richness was positively related to carbon stock and timber stock in terra firme forests, whereas it was negatively related to NTFP abundance in white sand forests. The positive species-carbon relationship across forests of the Guiana Shield is in line with the effects described by hypotheses such as the ‘niche complementarity’ and ‘selection effect’^10^ and is in line with previous findings at regional spatial scales^6,21^. To our knowledge, the relationship between species richness and timber stock has not been previously analysed for tropical forests. Interestingly, the observed positive species-timber relationship in terra firme forests of the Guiana Shield contrasts with the negative species-timber relationship found for subtropical forests in both the U.S.A. and Spain^20^, although this may be explained by the difference in ecosystems. The non-significant species-NTFP abundance relationship across the Guiana Shield and the negative relationship within white sand forests seems to contradict previous findings. Steur et al*.*^24^ found a negative species-NTFP abundance relationship for tropical forests in Suriname. However, this negative relationship was found across multiple forest types, including flooded forests that had low species richness and high NTFP abundance. These flooded forests most likely influenced the species-NTFP abundance relationship across all forest types.

In contrast to the relationship between species richness and carbon stock, no mechanism has been proposed for how species richness would influence commercial timber stock and NTFP abundance. Although our results suggest that species richness had a positive relationship with timber, the relationship was not found within multiple biogeographical subregions. For NTFP abundance, species richness did not contribute to explaining variation when variation across biogeographical subregions was accounted for (i.e. was included as an explanatory variable). We here tentatively propose that both commercial relevant timber stock and NTFP abundance are driven by variation in species floristic composition, rather than by species richness. For services such as commercial timber and NTFP provisioning, only a subset of all species is relevant (in this study, 9.4% of all morphospecies for timber and 3.8% for NTFPs), and such subsets are likely not random selections. For example, for Suriname, it was found that variation in commercially relevant NTFP abundance was driven by a particularly small selection of NTFP producing species with high abundances (referred to as ‘NTFP oligarchs’)^24^, and for commercial relevant timber stock, it is commonly known that selections tend to include more abundant than rare species. Additionally, as the relative abundance of species tends to vary across floristic regions in Amazonia, where, for example, certain species are dominant in particular forest types and biogeographical regions^31,32^, it can be expected that commercial timber stock and NTFP abundance are determined by floristic composition. In support, for NTFP abundance in Suriname tropical forests, Steur et al*.*^24^ found that floristic composition was a stronger predictor of NTFP abundance than species richness.

Across all of Amazonia, species richness had a positive relationship with carbon stock, but only when variation among biogeographical regions was accounted for. The positive species-carbon relationship across Amazonia partly contrasts with previous findings at continental spatial scales^11,13^. When variation across climatic and/or edaphic variables was accounted for, Sullivan et al*.*^13^ found no significant species-carbon relationship across South-America, while Poorter et al*.*^33^ did find a positive relationship across Meso- and South-America. Here, we propose that accounting for differences among biogeographical regions can explain the previously found contrasts at continental spatial scales. In our dataset, for individual regions, we found either a positive relationship or a non-significant, but weakly positive, relationship between carbon stock and species richness (Fig. 2). However, when the data were aggregated across all regions, this resulted in a non-significant, and weakly negative, relationship. This reflects a known statistical phenomenon referred to as a ‘Simpson’s paradox’^34^, in which a relationship appears in multiple distinct groups but disappears or reverses when the groups are combined. Additional post-hoc tests of leaving one region out at a time showed that this pattern was not dependent of any particular biogeographical region. This is the first time that an analysis based on empirical data provides evidence for a Simpson’s paradox in species-ecosystem service relationships.

It is likely that the observed differences in carbon stock across the biogeographical regions of Amazonia are influenced by multiple factors. For example, the biogeographical regions used in our analyses were recognised according to differences in substrate history, geological age and floristic composition, which could all contribute to variation in carbon stock. The substrate history and geological age of the biogeographical regions have been related to differences in soil fertility^35^, while multiple spatial gradients in floristic composition identified across the Amazon coincide with a spatial gradient in wood density^28^. However, further analysis is needed to obtain better insight into the relative contributions of these and other variables to explain the observed variation in carbon stock across the biogeographical regions. This requires data on multiple environmental variables, including floristic composition, climatic variables such as the length of the dry period, soil conditions, and intensity of disturbance.

In our analyses, terra firme forests determined the relationship of species richness with the carbon stock, timber stock, and NTFP abundance across the datasets. Although this is most likely the effect of unequal sample sizes, with terra firme forests being the dominant forest type in terms of sample size (n = 130 vs. n = 21 for the Guiana Shield dataset; n = 257 vs. n = 26 for the Amazonia dataset), we expect that the observed relationships reflect the general pattern. Terra firme forests are the most dominant forest type in terms of geographical area^32^ and were representatively sampled. Regardless, the analyses per forest type had added value. The significant relationship between species richness and NTFP abundance in white sand forests across the Guiana Shield would otherwise have been overlooked.

Due to the known scarcity of reliable and adequate information on which timber and NTFP species are being commercially traded^36–39^, we used a fixed set of timber and NTFP species to apply across the Guiana Shield plots. However, in reality, timber and NTFP species can be expected to vary according to socio-economic factors, such as culture, access, and harvest costs, which may change over space and time. Therefore, estimates of timber stock and NTFP abundance can be expected to differ across spatial gradients, and thus, their possible relationships with species richness cannot be easily generalised. To circumvent this, timber stock and NTFP abundance would have to be estimated on the basis of ‘flexible’ species selections that can change according to local socio-economic contexts. To this end, detailed information on both commercially relevant timber and NTFP species is urgently needed. Yet, for our study area, we did not observe major differences in selected species, and we included broad selections of species, which should make timber stock and NTFP abundance robust against small deviations in species selection. It must be noted that our approach of quantifying commercial relevant timber stock and NTFP abundance does not consider the value of timber and NTFPs for subsistence use. In addition, NTFPs can also be derived from other growth forms, such as lianas, shrubs and herbs. Last, because NTFP production data was not available we used NTFP abundance as a proxy for NTFP stock, following similar assessments of NTFP stock ^24,40^. A limitation of this approach is that each NTFP species individual has an equal contribution to NTFP stock, whereas it can be expected that large individuals may have a larger contribution than smaller individuals and that production volumes can differ for different types of NTFPs, for example barks vs. seeds.

Our findings illustrate the importance of considering environmental stratification and spatial scale when analysing relationships between biodiversity and ecosystem services. First, environmental stratification can help detect relationships that are otherwise obscured by environmental heterogeneity. For example, although the association between species richness and carbon stock across Amazonia was relatively weak (explaining ~ 3% of total variation vs. ~ 15% in the Guiana Shield) and was obscured by variation in carbon stock across biogeographical strata, by using environmental stratification the positive relationship remained detectable. Second, environmental heterogeneity tends to vary with spatial scale; therefore, its importance needs to be checked according to spatial scale. For example, at the regional scale of the Guiana Shield, biogeographical subregions explained a moderate amount of variation in carbon stock (~ 20%), while at the spatial scale of Amazonia, biogeographical regions explained more than half of total variation in carbon stock (~ 55%). Such an increase and ultimate importance of variation across biogeographical strata might also explain the absence of a significant relationship between species richness and carbon stock across African and/or Asian tropical forests as reported by Sullivan et al*.*^13^.

In our analyses, we found evidence of a positive relationship between species richness and carbon stock across and within Amazonia. This supports the notion that win–win scenarios are possible in conservation approaches, where, for example, REDD+ can be expected to help conserve tropical forests that contain large amounts of carbon stock and high concentrations of species^9^. However, we conclude that species richness is not always a strong predictor of biomass-based ecosystem services. In our analyses, NTFP abundance was not driven by species richness, and we ultimately expect the same for timber stock. We expect that differences in floristic composition, linked to differences across forest types and biogeographical strata, will be more relevant than species richness in explaining variation in timber stock and NTFP abundance. This would mean that conserving timber and NTFP related ecosystem services requires the development of additional region-specific strategies that account for differences in floristic composition. For example, areas with high concentrations of timber or NTFPs could be considered in the designation of multiple use protected areas^41^, such as the extractive reserves in Brazil, or be included as ‘high conservation value areas’ (HCVAs) in sustainable forest management certification^42^.

## Methods

### Guiana Shield dataset

**Figure 3:**
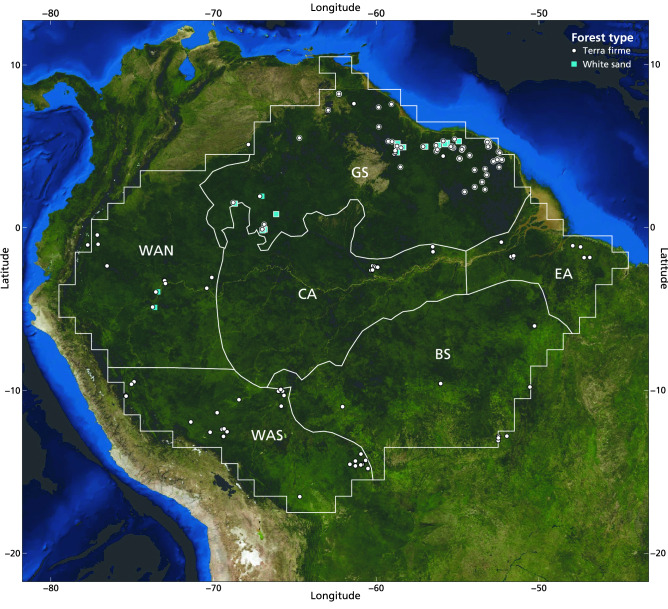
Map of the 283 1-ha old-growth lowland tropical forest plots across Amazonia. The plots of the Guiana Shield dataset are marked with an additional white contour (See Supplementary Appendix S1, Figure S1.1 for the Guiana Shield dataset plots only). For each plot, the forest type is indicated by symbols, where white circle = terra firme forest, and blue square = white sand forest. Approximate borders of the six biogeographical regions of Amazonia, reproduced from ter Steege et al.^32^, are indicated with white lines. Abbreviations for the regions are GS = Guiana Shield, BS = Brazilian Shield, WAN = north-western Amazonia, WAS = south-western Amazonia, CA = central Amazonia, and EA = eastern Amazonia. Figure created in R^43^, background satellite imagery of South America by NASA^44^.

We compiled a dataset of 151 1-ha lowland tropical forest plots spanning the Guiana Shield biogeographical region in Amazonia, most from the Amazon Tree Diversity Network (ATDN) (Fig. 3; Table 3; references provided in Table S1.3). These plots represent old-growth tropical forest vegetation on terra firme soils with limited signs of anthropogenic disturbance. In each plot, all trees and arborescent palms, hereafter referred to as ‘woody species’, with a diameter at breast height (‘DBH’; 1.3 m) of ≥ 10 cm, were measured and identified to at least a unique morphospecies. In line with previous large-scale assessments of relationships between plant diversity and ecosystem services^11,13^, at least 60% of the stems had been identified up to the species level, at least 80% up to the genus level and 100% up to the family level. Taxonomy followed the ‘Dynamic Amazon Tree Checklist’ (updated version 20200422)^47^.

**Table 3:** Overview of the two datasets used in this study, showing sample size, geographical extent and the number of biogeographical strata and forest types included.

	Guiana Shield dataset	Amazonia dataset
Number of 1-ha plots	151	283
Rectangular geographical extent	1.7 × 10^6^ km^2^	9.4 × 10^6^ km^2^
Number of biogeographical strata	4 subregions	6 regions
Number of forest types	2	2
	Mean ± s.d	Mean ± s.d
Aboveground carbon stock (Mg ha^−1^)	212.2 ± 49.48	175.34 ± 59.13
Timber stock (m^3^ ha^−1^)	119.8 ± 67.82	NA
NTFP abundance (stems ha^−1^)	102.71 ± 57.94	NA
Woody species richness (species ha^−1^)	123.93 ± 50.37	141.60 ± 62.96

Biogeographical strata and forest types were recognized after Stropp^30^, ter Steege et al*.*^32,45^ and ter Steege & Zondervan^46^. In addition, for each of the three ecosystem service stock components and woody species richness showing their mean value and standard deviation (mean ± s.d.). See Table S1.3 for a summary of the plot data, including references. NTFP abundance = abundance of species that produce non-timber forest products.

For these plots, we calculated woody species richness (species ha^−1^), and the stock component of the ecosystem services carbon storage, timber provisioning, and non-timber forest product (‘NTFP’) provisioning. Aboveground carbon stock per plot (Mg ha^−1^), hereafter referred to as ‘carbon stock’, was calculated following Sullivan et al*.*^13^: aboveground biomass was estimated from stem diameter, height, and wood density using the pantropical allometric equation of Chave et al*.*^48^. For this, stem height was estimated from stem diameter using biogeographical region-specific ‘Weibull’ equations developed by Feldpausch et al.^49^, and carbon stock was estimated by multiplying the biomass with a factor of 0.471. Wood density was retrieved from an appended version of the global wood density database by Chave et al.^50^ (ter Steege et al*.* in prep.; version 20,200,401). Applying a different allometric equation calibrated for the neotropics that did not require separate height estimation did not result in significantly different estimates (Supplementary Appendix S1).

Timber stock per plot (m^3^ ha^−1^), hereafter referred to as ‘timber stock’, was estimated by calculating the volume of tree species that had been recently commercially traded. Following Piponiot et al*.*^36^, we identified commercially relevant timber species as all timber tree species that have been reportedly commercially traded over the last 25 years (1995–2020) in at least one of the geographical areas included (See Table S1.1 for the references), and we considered trees with DBH ≥ 50 cm eligible for harvest under local forestry laws. This identified 727 commercially relevant timber tree species in our plots (9.4% of all morphospecies). Tree volume was estimated from tree diameter using the moist-forest allometric equation of Chave et al*.*^51^. Following Steur et al.^24^, the number of tree and arborescent palm individuals that produce commercially relevant NTFPs, hereafter referred to as ‘NTFP abundance’, was counted per plot (stems ha^−1^) as a proxy for NTFP stock. For this, we counted the tree and palm individuals of species that are known to produce NTFPs, hereafter referred to as ‘NTFP species’, that have been commercially traded over the last 25 years (1995–2020) in at least one of the geographical areas included. This identified 295 commercially relevant NTFP species present in our plots (3.8% of all morphospecies), which were mainly used as food, crafts, medicines and for cultural services (e.g. for rituals)(See Table S1.2, including references).

### Amazonia dataset

We combined the Guiana Shield data with data from 132 1-ha tropical forest plots published by Sullivan et al*.*^13^ to create a dataset of 283 plot measurements of woody species richness and carbon stock across Amazonia (Fig. 3; Table 3; references provided in Table S1.3). This also added 14 additional plots for the Guiana Shield region. Taxonomic precision and the minimum DBH used by Sullivan et al*.*^13^ were comparable to the Guiana Shield dataset, see Supplementary Appendix S1 for more information.

### Environmental covariables

To investigate how relationships with woody species richness changed according to environmental heterogeneity, we used forest type and biogeographical strata as categorical environmental covariables.

After ter Steege et al*.*^32,45^, we classified all plots into two main forest types on well-drained soils (Fig. 3): forests on brown soils, hereafter referred to as ‘terra firme forests’ (TF; n = 130 for Guiana Shield dataset, n = 257 for Amazonia dataset) and forests on white sands, hereafter referred to as ‘white sand forests’ (PZ; n = 21 for Guiana Shield dataset, n = 26 for Amazonia dataset). These forest types differ mainly in physiognomy, species composition, and substrate origin, and their sample sizes reflect the geographical coverage of these forest types, where terra firme forests cover more than 50% of Amazonia and white sand forests just under 5%^32^. In addition, we classified all plots into six biogeographical regions (Fig. 3), and the plots from the Guiana Shield database into four biogeographical subregions (Figure S1.1). After ter Steege et al*.*^32,45^ we recognised the following Amazonian biogeographical regions: the Guiana Shield (GS; n = 165), the Brazilian Shield (BS; n = 9), north-western Amazonia (WAN; n = 21), south-western Amazonia (WAS; n = 51), central Amazonia (CA; n = 22) and eastern Amazonia (EA; n = 15). Based on the Guiana Shield ‘forest regions’ identified by ter Steege & Zondervan^46^ and revised after floristic analyses carried out by Stropp^30^, we recognized the following forest subregions: forests of the northern Pleistocene sands (NPS, n = 56), south-western Pleistocene sands in the upper Rio Negro region (SWPS, n = 11), southern Guiana Shield (SGS, n = 63) and north-western Guiana Shield (NWGS, n = 21). These biogeographical strata have been identified according to differences in substrate history, geological age and floristic composition. More information on forest types and biogeographical strata is provided in Supplementary Appendix S1.

Although soil type information was also available for the Guiana Shield dataset, we found high collinearity of soil class with both biogeographical subregions and forest types. Therefore, we excluded it from further analyses. For reference, information on soil type is included in Supplementary Appendix S1.

### Statistical analyses

We used standard linear models to analyse relationships between species richness and ecosystem service stock components and to explore how biogeographical strata and forest types influenced these relationships. To analyse how species richness was related to the different ecosystem services while accounting for potential confounding variables, we used multiple linear regression models that were optimised using a backward model selection procedure proposed by Crawley^52^. All dependent variables followed an approximate normal distribution, independent variables were checked for multicollinearity, and each model showed approximately homogenous variances. We used the relative contribution to the total amount of variation explained as a measure of the relative importance of the variables. The relative contribution was calculated according to the amount of explained variation added when a variable is included, taking the average of this amount across all possible variable orders in the model. In this way, the relative contribution of the variable to R^2^ is compensated for the amount of variation already explained by other variables in the model^53^.

We tested for significant variation in ecosystem service components and woody species richness across biogeographical strata and forest types by using analysis of variance F-tests and applied post-hoc Tukey tests to assess any differences among the groups. The Tukey post-hoc test adjusts the p-value for multiple testing, controlling for the increased chance of obtaining a false positive when multiple tests are conducted in sequence (Type I error). We checked for spatial autocorrelation in the model residuals by plotting them in a map and by performing Moran’s I tests. Although we found significant spatial autocorrelation for the three models based on the Guiana Shield data and the model based on the Amazonia data (all four *p* < 0.0200), sensitivity analyses by leaving one biogeographical stratum out at a time did not result in significant differences. The spatial autocorrelation is believed to be inherent to our data, because some of the plots have the same longitude and latitude due to GPS limitations at the time of their census (e.g. the plots ALP-01 and ALP-30 from Sullivan et al.^13^).

All statistical analyses were conducted using R^43^. Additional details on the statistical analyses and software used are included in Supplementary Appendix S1. Supplementary Results are provided in Supplementary Appendix S2.

## Supplementary Information

Supplementary Information.

## Data Availability

Plot data is provided in the Supporting Information.
