# Supplementary material for: Relationships between species richness and ecosystem services in Amazonian forests strongly influenced by biogeographical strata and forest types
Source: Sci Rep. 2022 Apr 8;12:5960. doi: 10.1038/s41598-022-09786-6 (PMC8993798; doi:10.1038/s41598-022-09786-6)
Supplement: Supplementary file 1 — Supplementary Information. [file 41598_2022_9786_MOESM1_ESM.docx]

## Contents Supporting Information

Contents

[Contents Supporting Information 1](#_Toc98087614)

[Annex 1: Methodology and Material 2](#_Toc98087615)

[Guiana Shield dataset 2](#_Toc98087616)

[Plant diversity indicators and ecosystem services 2](#_Toc98087617)

[Amazonia dataset 3](#_Toc98087618)

[Environmental covariables 4](#_Toc98087619)

[Review of aboveground carbon stock estimation methods 5](#_Toc98087620)

[Statistical analyses 7](#_Toc98087621)

[Software 9](#_Toc98087622)

[References 9](#_Toc98087623)

[Table S1.1. List of commercially relevant timber tree species in the Guiana Shield region 11](#_Toc98087624)

[Table S1.2. List of commercially relevant NTFP taxa in the Guiana Shield region 18](#_Toc98087625)

[Table S1.3. Summary of plot data 22](#_Toc98087626)

[Annex 2: Supplementary results 30](#_Toc98087627)

[Guiana Shield linear relationships across environmental covariables 30](#_Toc98087628)

[Amazonia linear relationships across environmental covariables 36](#_Toc98087629)

### Annex 1: Methodology and Material

#### Guiana Shield dataset

We compiled a dataset of 151 1-ha lowland tropical forest plots spanning the Guiana Shield region in Amazonia (Figure S1.1). These plots had been previously recorded by various authors, and most are included in the Amazon Tree Diversity Network (ATDN) (Table S1.3). Of the 151 plots, two were 0.98 ha in size but were treated as 1 ha plots. All of the plots represented old-growth tropical forest vegetation on terra firme soils, where all but two had shown visual signs of limited anthropogenic disturbance. In each plot, all trees and arborescent palms with a minimum diameter at breast height of 10 cm (‘DBH’, 1.3 m), hereafter referred to as ‘woody species’, had been measured and identified. In line with previous plant diversity and ecosystem services assessments at relatively large geographical extents (Poorter et al., 2015; Sullivan et al., 2017) at least 60% of the stems had been identified up to the species level, at least 80% up to the genus level and 100% up to the family level. Taxonomy of the woody species was updated following the ‘Dynamic Amazon Tree Checklist’ (ter Steege et al. 2019; updated version 20200422).


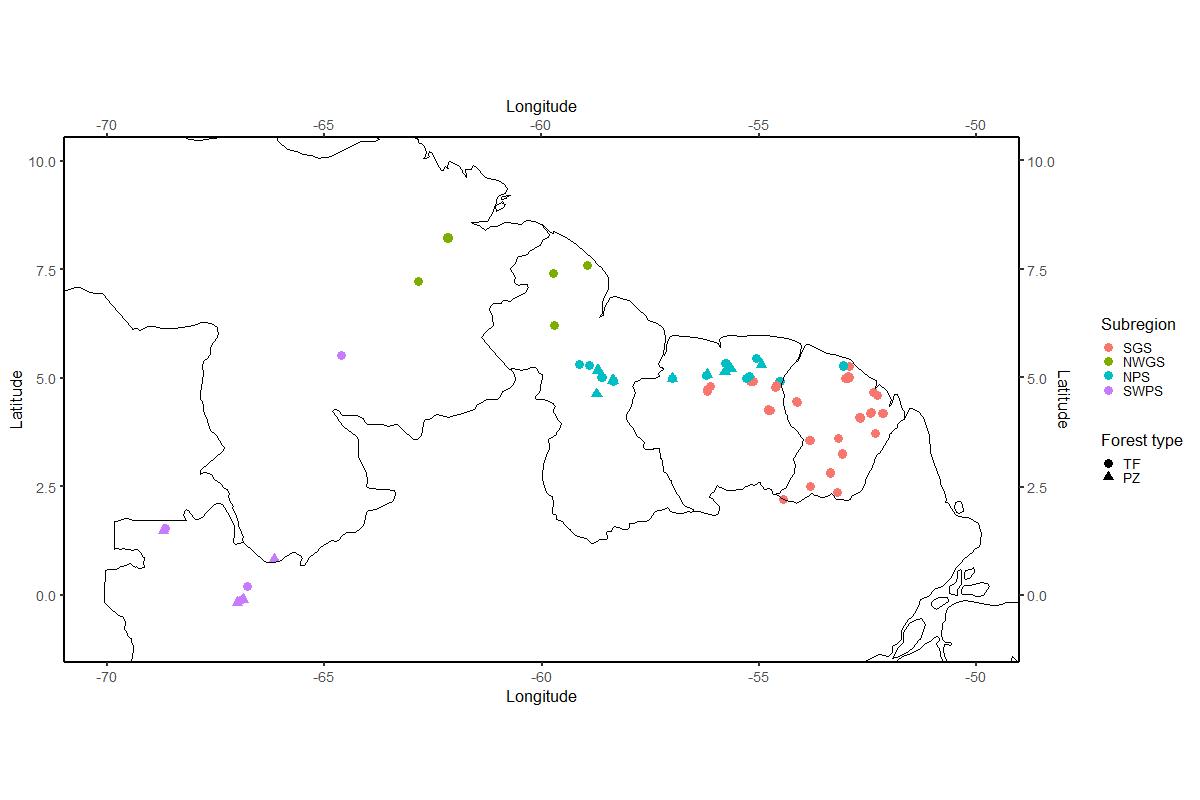


**Figure S1.1.** Map of the 151 1-ha Guiana Shield dataset plots. For each plot, the forest type is indicated by symbols, where circle = terra firme forest (TF, n = 130), and triangle = white sand forest (PZ, n = 21), and subregion is indicated by colour, where red = forests of the southern Guiana Shield (SGS, n = 63), green = forests of the north-western Guiana Shield (NWGS, n = 21), turquoise = forests of the northern Pleistocene sands (NPS, n = 56), and purple = forests of the south-western Pleistocene sands (SWPS, n = 11). Country borders reproduced after the ggplot2 world database (Wickham, 2016) are indicated by black lines.

#### Plant diversity indicators and ecosystem services

For all Guiana Shield dataset plots, we calculated woody species richness (species ha^-1^) and the stock component of three ecosystem services: carbon storage, timber provisioning and non-timber forest product (NTFP) provisioning. Carbon storage was indicated by the aboveground carbon stock (in Mg ha^-1^), timber provisioning was indicated by the commercially relevant timber stock (m^3^ ha^-1^), and non-timber forest product (NTFP) provisioning was indicated by the number of stems of commercially relevant non-timber forest product-producing woody plant species, or ‘NTFP abundance’ (stems ha^-1^). Carbon stock on itself is considered an ecosystem service, whereas timber stock and NTFP stock represent the potential of their services. Although not all timber stock and NTFP stock will be ultimately used, it is generally assumed that timber stock and NTFP stock will be positively related to their use. Therefore, in this paper all three services stocks are used as representatives for their ecosystem services.

Aboveground carbon stock per plot was estimated following Sullivan et al. (2017). Here, first tree aboveground biomass was estimated on the basis of stem diameter, height, and wood density by using the pantropical allometric equation of Chave et al. (2014). Tree height was estimated on the basis of tree diameter by using biogeographical region specific ‘Weibull’ equations developed by Feldpausch et al. (2012). Wood density was retrieved from an appended version of the Chave et al. (2009) global wood density database, specifically for Amazonian woody species (ter Steege et al., in prep.; version 20200401). Second, tree biomass was converted to carbon stock by applying a conversion factor of 0.471.

We considered using a single allometric equation that was calibrated for the neotropics specifically and does not use height as input instead of the approach of Sullivan et al. (2017), but we found that using such an equation did not lead to significant different estimates. Ultimately, we wanted to keep the method of calculating carbon stock consistent across datasets, and we therefore choose to use the approach of Sullivan et al. (2017). For more details, see the section comparing the Guiana Shield and Amazonia datasets, below.

Commercially relevant timber stock per plot was estimated similar to Piponiot et al. (2019). First, a list of recently commercially traded timber tree species was compiled on the basis of the tree species that were reportedly traded for timber in at least one of the relevant countries or country states for the last 25 years (1995-2020). Relevant countries or country states included: Brazil with the states of Pará and Amapá, Venezuela with the states of Bolivar and Amazonas, Guyana, Suriname, and French Guiana. For these relevant countries and country states we compiled a list of 1184 reported timber tree species, 727 of which were present in our plots. See Table S1.1 (below) for an overview of the morphospecies included and used sources. Second, for each plot the stem volume was calculated for individuals of the relevant tree species with DBH ≥ 50 cm, in accordance to local forestry laws. Stem volume was calculated by using the neotropical moist-forest allometric equation of Chave et al. (2005).

Following Steur et al. (2021), the number of tree and arborescent palm individuals that produce commercially relevant NTFPs, hereafter referred to as ‘NTFP abundance’, was counted per plot (stems ha^-1^) as a proxy for NTFP stock.. Similar to the approach for timber stock, a list was compiled of tree and arborescent palm species that were reported to have produced commercially traded non-timber forest products in at least one of the relevant countries or country states for the last 25 years (1995-2020). For the relevant countries and country states we compiled a list of 216 woody NTFP species and 7 woody NTFP genera, of which 295 morphospecies were present in our plots. See Table S1.2 (below) for an overview of the morphospecies included and used sources.

#### Amazonia dataset

We combined the Guiana Shield data with data from 132 1-ha tropical forest plots published by Sullivan et al. (2017) to create a dataset of 283 plot measurements of woody species richness and carbon stock across Amazonia (Figure 1; Table 1; references provided in Table S1.3). Taxonomic precision and the minimum DBH used by Sullivan et al. (2017) was comparable to our Guiana Shield dataset data. The data taken from Sullivan et al. (2017) also included 14 additional plot measurements on the Guiana Shield that were not included in the Guiana Shield dataset.

#### Environmental covariables

To investigate how relationships with woody species richness changed according to environmental heterogeneity, we used forest type and biogeographical strata as categorical environmental covariables.

For the entire Amazonia dataset (including the Guiana Shield dataset), we classified all available plots into two main non-flooded forest types, recognized after ter Steege et al., (2013) and ter Steege, Henkel, et al. (2019): forests on brown soils, hereafter referred to as ‘terra firme forests’ (TF; n = 130 for Guiana Shield dataset, n = 257 for Amazonia dataset) and forests on white sands, hereafter referred to as ‘white sand forests’ (PZ; n = 21 for Guiana Shield dataset, n = 26 for Amazonia dataset). These forest types mainly differ in physiognomy, species composition, and substrate origin, and their sample sizes reflect the geographical coverage of these forest types, where terra firme forests cover more than 50 % of Amazonia and white sand forests just under 5 % (ter Steege, Henkel, et al., 2019). Both forest types represent forests that occur on well-drained, never inundated soils, but white sands forests occur on bleached and leached, nutrient-poor sandy soils (Albic Arenosols), while the terra firme forests on brown soils include brown sands (sand, loam, clay) and Leptosols. Previous research has found that terra firme forests on brown soils are generally relatively tree species rich (Stropp, 2011; ter Steege, Henkel, et al., 2019; ter Steege & Zondervan, 2000), while white sand forests are generally relatively species poor (Stropp, 2011; ter Steege et al., 2013; ter Steege, Henkel, et al., 2019).

For the entire Amazonia dataset (including the Guiana Shield dataset), we classified all available plots into six biogeographical regions (Figure 1), recognized after ter Steege et al., (2013) and ter Steege, Henkel, et al. (2019): the Guiana Shield (GS; n = 165), the Brazilian Shield (BS; n = 9), north-western Amazonia (WAN; n = 21), south-western Amazonia (WAS; n = 51), central Amazonia (CA; n = 22) and eastern Amazonia (EA; n = 15). These biogeographical regions have been identified according to differences in substrate history, geological age and floristic composition. Here, both GS and BS dominated by relatively nutrient poor igneous and metamorphic rocks but are spatially separated from each other, forming distinct floristic clusters. Both WAN and WAS are both dominated by relatively nutrient rich Andean sediments but differ in mean annual precipitation, where WAN is wetter than WAS, forming two different floristic clusters. Last, both CA en EA are different mixtures of nutrient poor sediments originating from GS, BS, WAN and WAS, forming two different floristic clusters.

For the Guiana Shield dataset, we classified the plots into four subregions of the Guiana Shield biogeographical region (Figure S1.1), recognized after the ‘forest regions’ identified by ter Steege & Zondervan (2000) and revised after the floristic analyses between West and East terra firme forests of the Guiana Shield region carried out by Stropp (2011). On the basis of differences in substrate history and age, and by differences in floristic composition, we recognized the following subregions: forests of the northern Pleistocene sands (NPS, n = 56), forests of the south-western Pleistocene sands in the upper Rio Negro region (SWPS, n = 11), forests of the southern Guiana Shield (SGS, n = 63) and forests of the north-western Guiana Shield (NWGS, n = 21). SGS and NWGS lie on the actual Guiana Shield formation and consist of soils that have developed from the relatively ancient Pre-Cambrian crystalline substrates. By contrast, NPS and SWPS lie on the periphery of the Guiana Shield formation and consist of varied weathered soils that have been deposited during the relatively younger Tertiary-Pleistocene (ter Steege & Zondervan, 2000). In general, the forests on the Guiana Shield formation have a higher fisher’s alpha (are more diverse) than the forests on the Pleistocene sands. However, there are also floristic differences between the forests on the Guiana Shield formation and between the forests on the Pleistocene sands. The forests of SGS (the ‘Guiana peneplain’) have a relatively higher fisher’s alpha than the forest of NWGS and different genera tend to dominate the forest (Stropp et al., 2009; ter Steege & Zondervan, 2000). In addition, the forests of SWPS have a higher alpha diversity than the forests of the NPS (Stropp, 2011).

Last, originally, we had also data available on the soil type of the Guiana Shield dataset plots. However, as we found that soil class was highly collinear to both biogeographical subregions and forest types, we therefore excluded it from our analyses. For reference, the classification of the soils is given below.

For the plots in the Guiana Shield dataset the main soil type was noted. We classified these soil types into three soil classes based on the description Amazonian soils and their putative relationships to forest productivity and species composition (Hawes et al., 2012; Quesada et al., 2012; Saatchi et al., 2008): brown sands (n = 64), Leptosols (n = 67) and Albic Arenosols (n = 21). Here, the class brown sands include fine textured, strongly weathered, brownish, sandy, loamy or clayey soils (i.e. Ferralic Arenosols, Haplic Ferralsols and Xanthic Ferralsols; Plinthosols; Haplic Acrisols and Humic Acrisols). They have a high water-permeability and due to the presence of organic material they are moderately fertile and have a moderate water-holding capacity. Brown sands can be either acidic (Acrisols) or neutral (Ferrasols), which potentially impacts the species composition. However, as Acrisols are rare on the Guiana Shield and are most similar to Ferrasols, we combined them into one class. The class Leptosols include shallow clayey to stony soils on weather-resistant rock, for example in the Guiana Shield on lateritic caps. They have a low water-permeability and can be relatively fertile but in general inhibit plant growth with their limited depth. Last, the class Albic Arenosols includes fine-textured, extremely weathered, white-bleached, sandy soils. These have a high water-permeability but due to little organic material, they have a low water-holding capacity and are one of the most infertile soils in the Guiana Shield.

Table S1.4 Overview of the number of forest types per biogeographical stratum for the Guiana Shield dataset (n = 151) and the Amazonia dataset (n = 284). Forest types are TF = terra firme forests and PZ = white sand forests. Biogeographical subregions of the Guiana Shield are SGS = Forests of the Southern Guiana Shield, NWGS = Forests of the North-Western Guiana Shield, NPS = Forests of the Northern Pleistocene sands, and SWPS = Forests of the South-Western Pleistocene sands. Biogeographical regions of Amazonia are GS = Guiana Shield, BS = Brazilian Shield, WAN = North-West Amazonia, WAS = South-West Amazonia, CA = Central Amazonia, and EA = Eastern Amazonia.

| **Guiana Shield dataset** | **SGS** | **NWGS** | **NPS** | **SWPS** |  |  | **Totals** |
| --- | --- | --- | --- | --- | --- | --- | --- |
| **TF** | 63 | 21 | 40 | 6 |  |  |  |
| **PZ** | 0 | 0 | 16 | 5 |  |  | 21 |
| **Totals** | 63 | 21 | 56 | 11 |  |  | 151 |
| **Amazonia dataset** | **GS** | **BS** | **WAN** | **WAS** | **CA** | **EA** |  |
| **TF** | 141 | 9 | 19 | 51 | 22 | 15 | 257 |
| **PZ** | 24 | 0 | 2 | 0 | 0 | 0 | 26 |
| **Totals** | 165 | 9 | 21 | 51 | 22 | 15 | 283 |

#### Review of aboveground carbon stock estimation methods

When reviewing the methodology of Sullivan et al. (2017) we noticed that they used a tree volume allometric equation based on stem diameter and stem height while the stem height measurements were not available. They used the stem diameter to also estimate the stem height using a separate region-specific tree height allometric equation. However, this presented approach does not follow proper error propagation as the error of the height estimate is not inputted into the tree volume allometric equation. In addition, Sullivan et al. (2017) for their pantropical analyses used a tree volume allometric equation calibrated with trees from across the tropics, while for our analyses we focus on the Neotropics only. Using a pantropical tree volume allometric equation might lead to a bias for neotropical trees. Both issues could potentially reduce the amount of variation that can be explained by plant diversity or environmental covariables. Therefore, we investigated the impact of these two issues by calculating the aboveground biomass for the Guiana Shield dataset using two approaches: the ‘Sullivan et al. (2017) approach’ sensu Sullivan et al. (2017) and a second, ‘alternative approach’, using a neotropical tree volume allometric equation that uses only tree diameter instead of also tree height.

For the alternative approach we used the moist forest tree diameter allometric equation by Chave et al. (2005). This equation was calibrated for south-American tropical forests and does not require height measurements. We did not use the different dry, moist and wet forest equations by Chave et al. (2005) because this would artificially introduce differences in our estimates on the basis of a hard climatic threshold. In addition, we did not use a separate allometric equation for palm biomass, as the comparison by Selaya et al. (2017) showed that using a palm-specific allometric equation does not necessarily improve the accuracy of biomass estimates. The aboveground biomass was converted to aboveground carbon stock by multiplying with the conversion factor 0.474.

Mean aboveground carbon stock estimated by the alternative approach was not significantly different from the mean aboveground carbon stock estimated by the Sullivan et al. (2017) approach (t-value = 0.72264, df = 301.64, p-value = 0.4705; Table S1.5). In addition, a linear regression model explaining the aboveground carbon stock estimated by the Sullivan et al. (2017) approach by the estimates by the Alternative approach showed that both estimates were highly related (R = 99.3%; Table S1.6, Figure S1.1). We therefore concluded that the impact of using the Sullivan et al. (2017) is likely to be minor.

**Table S1.5**. Estimated aboveground carbon stock across the 151 1 ha plots of the Guiana Shield dataset, using the method of Sullivan et al. (2017) and the alternative method (see text). Showing the mean with standard deviation, the minimum and the maximum.

| **Aboveground carbon stock (Mg ha-1) across 151 1 ha Guiana Shield plots** | **Mean ± SD** | **min - max** |
| --- | --- | --- |
| **Method Sullivan et al. 2017** | 212.20 ± 49.48 | 76.30 - 350.60 |
| **Alternative method** | 207.90 ± 51.37 | 70.49 - 360.79 |

**Table S1.6**. Detailed results for the bivariate linear model of aboveground carbon stock values across the Guiana Shield dataset (n = 151) estimated by the Sullivan et al. (2017) approach predicted by the estimates by the alternative approach (see text). Showing model coefficients with its standard error (SE), the t-test value, the p -value of the t-test (H0: b = 0), and the R^2^ of the total model.

| Model | | Coefficient | SE | t-value | p-value | R^2^ |
| --- | --- | --- | --- | --- | --- | --- |
| Model AGC_Sullivan_ ~ AGC_Alternative_ | |  |  |  |  |  |
|  | Intercept | 12.684816 | 1.457244 | 8.705 | 5.53E-15 |  |
|  | AGC_Alternative_ | 0.959644 | 0.006806 | 141.002 | < 2e-16 |  |
|  |  |  |  |  |  | 0.9926 |


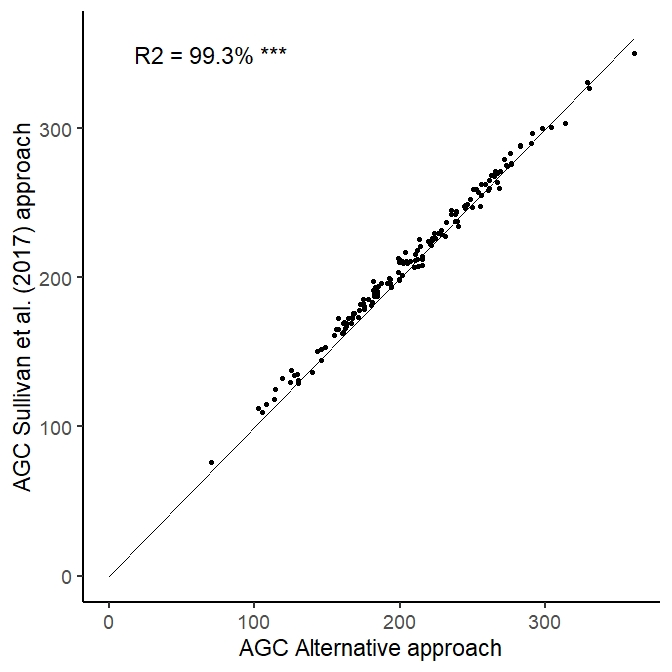


**Figure S1.2.** Comparison of aboveground carbon stock (AGC, in Mg ha^-1^) for the 151 1-ha Guiana Shield plots estimated by the Sullivan et al (2017) approach (y-axis) and the Alternative approach (x-axis). A perfect relationship between both estimation approaches is indicated by the black line.

#### Statistical analyses

We used standard linear models to analyse relationships between species richness and ecosystem service stock components and to explore how the environmental covariables influenced these relationships. To test for direct and indirect effects of species richness and also test the extent to which species richness and environmental covariables independently contributed to explaining variation in ecosystem services, we used multiple linear regression models that were optimised using a backward model selection procedure proposed by Crawley (2015). In this procedure, a full model containing all relevant and non-collinear variables is optimized by excluding one variable at a time, and testing with a Log-Likelihood Ratio test whether this does not lead to a significant difference in the amount of variation explained. This is repeated until no variable can be removed without the model explaining a significantly different amount of variation.

Regarding linear model assumptions, all dependent variables, here all three ecosystem services, followed an approximate normal distribution (Figure S1.4-S1.5). Log transformation did not seem to significantly improve distributions. Multicollinearity between explanatory variables might lead to erroneous exclusion of explanatory variables under model optimization. Therefore, we checked for multicollinearity between the explanatory variables in the full model by omitting any variables with a Variance Inflation Factor (VIF) larger than 3 (after Zuur et al., 2010). The sample sizes of our covariables were unequal (forest type, biogeographical region, biogeographical subregion), which could have potentially led to heterogenous model variances and therefore erroneous exclusion of the covariables under model optimization. However, we checked the residuals vs the fitted values of all of our full and optimized models and did not detect clear heterogeneity for our models. Last, low sample sizes could lead to a low power to detecting relationships and could lead to potential model overfitting. Generally, a conservative value of at least 10 samples per parameter is advocated for linear regression (Crawley, 2015). Although in one case the number of samples per parameter was lower than 10 (i.e. for the region of the Brazilian Shield, n = 9), all other parameters in our models were above the conservative number. In addition, to check for effects of overfitting, we also carried out a regression analysis for each covariable parameter separately.


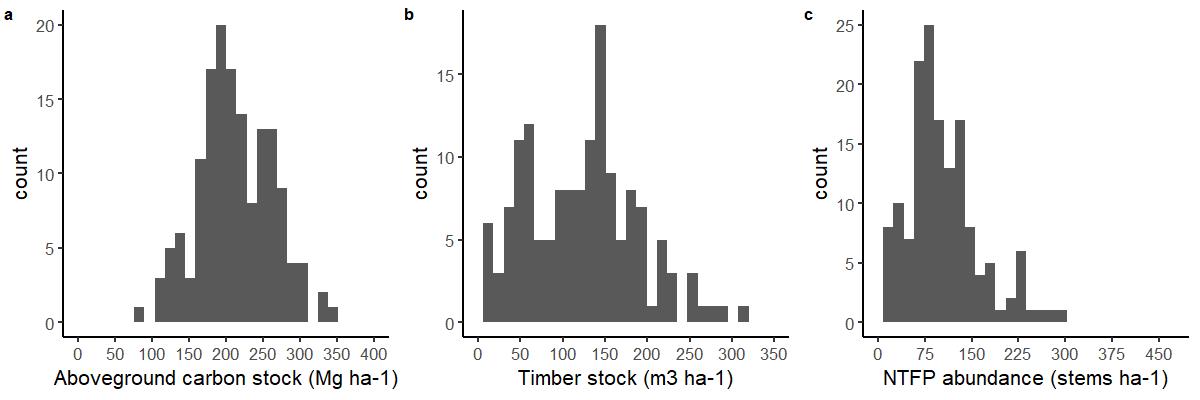


**Figure S1.4** Histograms of aboveground carbon stock (Mg ha^-1^; panel a), timber stock (m^3^ ha^-1^; panel b) and NTFP abundance (stems ha^-1^; panel c) in the Guiana Shield dataset (n = 151).


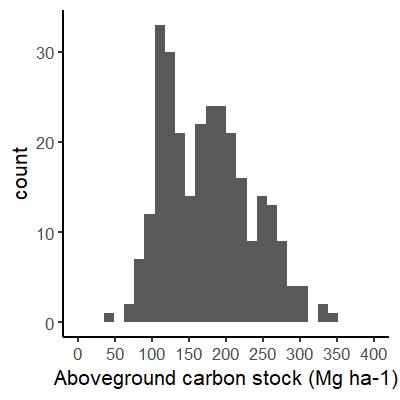


**Figure S1.5** Histogram of aboveground carbon stock (Mg ha^-1^) in the Amazonia dataset (n = 283).

We used the relative contribution to the total amount of variation explained (R^2^; after Lindeman et al., 1980) as a measure of relative importance of variables because it can be calculated for both continuous as categorial variables. The relative contribution of each variable is calculated according to the amount of explained variation that is added when a variable is included, and taking the average of this amount across all possible variable orders in the model. In this way, the relative contribution of the variable to R^2^ is compensated for the amount of variation already explained by other variables in the model.

We tested if there was significant variation in ecosystem services and/or plant diversity indicators across soil classes, forest types and biogeographical regions, by using analysis of variance F-tests. If significant variation across groups was detected, we applied post-hoc Tukey tests to assess the differences among the groups. The Tukey Post-hoc test adjusts the p-value for multiple testing, controlling for the increased chance of obtaining a false positive when multiple tests are carried out in sequence (Type I error).

As stem density, basal area and wood density had been used to estimate aboveground carbon stock, these variables are structurally collinear. Therefore, these variables were not used for model optimization of aboveground carbon stock models. The structural collinearity of stem density, basal area and wood density with timber stock and NTFP abundance was expected to be less problematic: for estimates of timber stock and NTFP abundance only a subset of all woody species had been used.

#### Software

All statistical analyses were carried out in R (R Core Team, 2020). In addition, we used the following R packages: *stringr* (Wickham, 2019) for general coding support; *vegan* (Oksanen et al., 2019) for calculation of all plant diversity indicators except for the Camargo evenness for which own code was used; *Hmisc* (Harrell Jr, 2020) for calculation of correlation matrices; *pastecs* (Grosjean & Ibanez, 2018) for standard variable statistics; *car* (Fox & Weisberg, 2019) to calculate variance inflation factors; *relaimpo* (Grömping, 2006) to calculate the relative contribution to R^2^; *multcomp* (Hothorn et al., 2008) to carry out post-hoc Tukey tests; and *ggplot2*, *ggrepel*, *raster* and *ggpubr* (Hijmans, 2020; Kassambara, 2019; Slowikowski, 2019; Wickham, 2016) for graphical output.

#### References

Chave, J., Andalo, C., Brown, S., Cairns, M. A., Chambers, J. Q., Eamus, D., Fölster, H., Fromard, F., Higuchi, N., Kira, T., Lescure, J.-P., Nelson, B. W., Ogawa, H., Puig, H., Riéra, B., & Yamakura, T. (2005). Tree allometry and improved estimation of carbon stocks and balance in tropical forests. *Oecologia*, *145*(1), 87–99. https://doi.org/10.1007/s00442-005-0100-x

Chave, J., Coomes, D., Jansen, S., Lewis, S. L., Swenson, N. G., & Zanne, A. E. (2009). Towards a worldwide wood economics spectrum. *Ecology Letters*, *12*(4), 351–366. https://doi.org/10.1111/j.1461-0248.2009.01285.x

Chave, J., Réjou-Méchain, M., Búrquez, A., Chidumayo, E., Colgan, M. S., Delitti, W. B. C., Duque, A., Eid, T., Fearnside, P. M., Goodman, R. C., Henry, M., Martínez-Yrízar, A., Mugasha, W. A., Muller-Landau, H. C., Mencuccini, M., Nelson, B. W., Ngomanda, A., Nogueira, E. M., Ortiz-Malavassi, E., … Vieilledent, G. (2014). Improved allometric models to estimate the aboveground biomass of tropical trees. *Global Change Biology*, *20*(10), 3177–3190. https://doi.org/10.1111/gcb.12629

Crawley, M. J. (2015). *Statistics - An introduction using R* (Second edi). John Wiley & Sons, Inc. https://doi.org/10.1002/9781119941750

Feldpausch, T. R., Lloyd, J., Lewis, S. L., Brienen, R. J. W., Gloor, M., Monteagudo Mendoza, a., Lopez-Gonzalez, G., Banin, L., Abu Salim, K., Affum-Baffoe, K., Alexiades, M., Almeida, S., Amaral, I., Andrade, a., Aragão, L. E. O. C., Araujo Murakami, a., Arets, E. J. M., Arroyo, L., Aymard C., G. a., … Phillips, O. L. (2012). Tree height integrated into pantropical forest biomass estimates. *Biogeosciences*, *9*(8), 3381–3403. https://doi.org/10.5194/bg-9-3381-2012

Fox, J., & Weisberg, S. (2019). *car: An R Companion to Applied Regression.* (Third Edition). Thousand Oaks CA: Sage. https://socialsciences.mcmaster.ca/jfox/Books/Companion/%0A

Grömping, U. (2006). Relative importance for linear regression in R: The package relaimpo. *Journal of Statistical Software*, *17*(1), 1–27. https://doi.org/10.18637/jss.v017.i01

Grosjean, P., & Ibanez, F. (2018). *pastecs: Package for Analysis of Space-Time Ecological Series* (R package version 2.2-3). https://cran.r-project.org/package=pastecs

Harrell Jr, F. E. (2020). *Hmisc: Harrell Miscellaneous* (R package version 4.4-0). https://cran.r-project.org/package=Hmisc

Hawes, J. E., Peres, C. A., Riley, L. B., & Hess, L. L. (2012). Landscape-scale variation in structure and biomass of Amazonian seasonally flooded and unflooded forests. *Forest Ecology and Management*, *281*, 163–176. https://doi.org/10.1016/j.foreco.2012.06.023

Hijmans, R. J. (2020). *raster: Geographic Data Analysis and Modeling* (R package version 3.0-12). https://cran.r-project.org/package=raster

Hothorn, T., Bretz, F., & Westfall, P. (2008). multcomp: Simultaneous Inference in General Parametric Models. *Biometrical Journal*, *50*(3), 346–363. https://doi.org/10.1002/bimj.200810425

Kassambara, A. (2019). *ggpubr: “ggplot2” Based Publication Ready Plots* (R package version 0.2.4). https://cran.r-project.org/package=ggpubr

Lindeman, R. H., Merenda, P. F., & Gold, R. Z. (1980). *Introduction to Bivariate and Multivariate Analysis*. Glenview, IL : Scott, Foresman and Comp.

Oksanen, J., Guillaume Blanchet, F Friendly, M., Kindt, R., Legendre, P., McGlinn, D., Minchin, P. R., O’Hara, R. B., Simpson, G. L., Solymos, P., Henry, M., Stevens, H., Szoecs, E., & Wagner, H. (2019). *vegan: Community Ecology Package* (R package version 2.5-6). https://cran.r-project.org/package=vegan

Piponiot, C., Rödig, E., Putz, F. E., Rutishauser, E., Sist, P., Ascarrunz, N., Blanc, L., Derroire, G., Descroix, L., Guedes, M. C., Coronado, E. H., Huth, A., Kanashiro, M., Licona, J. C., Mazzei, L., D’Oliveira, M. V. N., Peña-Claros, M., Rodney, K., Shenkin, A., … Hérault, B. (2019). Can timber provision from Amazonian production forests be sustainable? *Environmental Research Letters*, *14*(6), 64014. https://doi.org/10.1088/1748-9326/ab195e

Poorter, L., van der Sande, M. T., Thompson, J., Arets, E. J. M. M., Alarcón, A., Álvarez-Sánchez, F. J., Ascarrunz, N. L., Balvanera, P., Barajas-Guzmán, G., Boit, A., Bongers, F., Carvalho, F. A., Casanoves, F., Cornejo-Tenorio, G., Costa, F. R. C., de Castilho, C. V., Duivenvoorden, J. F., Dutrieux, L. P., Enquist, B. J., … Peña-Claros, M. (2015). Diversity enhances carbon storage in tropical forests. *Global Ecology and Biogeography*, *24*(11), 1314–1328. https://doi.org/10.1111/geb.12364

Quesada, C. A., Phillips, O. L., Schwarz, M., Czimczik, C. I., Baker, T. R., Patiño, S., Fyllas, N. M., Hodnett, M. G., Herrera, R., Almeida, S., Alvarez Dávila, E., Arneth, A., Arroyo, L., Chao, K. J., Dezzeo, N., Erwin, T., di Fiore, A., Higuchi, N., Honorio Coronado, E., … Lloyd, J. (2012). Basin-wide variations in Amazon forest structure and function are mediated by both soils and climate. *Biogeosciences*, *9*(6), 2203–2246. https://doi.org/10.5194/bg-9-2203-2012

R Core Team. (2020). *R: A Language and Environment for Statistical Computing* (4.0.2.). R Foundation for Statistical Computing. https://www.r-project.org

Saatchi, S., Buermann, W., ter Steege, H., Mori, S., & Smith, T. B. (2008). Modeling distribution of Amazonian tree species and diversity using remote sensing measurements. *Remote Sensing of Environment*, *112*, 2000–2017. https://doi.org/10.1016/j.rse.2008.01.008

Selaya, N. G., Zuidema, P. A., Baraloto, C., Vos, V. A., Brienen, R. J. W., Pitman, N., Brown, F., Duchelle, A. E., Araujo-Murakami, A., Oliveira Carillo, L. A., Vasquez Colomo, G. H., Meo Chupinagua, S., Fuentes Nay, H., & Perz, S. (2017). Economically important species dominate aboveground carbon storage in forests of southwestern Amazonia. *Ecology and Society*, *22*(2), art40. https://doi.org/10.5751/ES-09297-220240

Slowikowski, K. (2019). *ggrepel: Automatically Position Non-Overlapping Text Labels with “ggplot2”* (R package version 0.8.1). https://cran.r-project.org/package=ggrepel

Steur, G., Verburg, R. W., Wassen, M. J., Teunissen, P. A., & Verweij, P. A. (2021). Exploring relationships between abundance of non-timber forest product species and tropical forest plant diversity. *Ecological Indicators*, *121*(December 2020), 107202. https://doi.org/10.1016/j.ecolind.2020.107202

Stropp, J. (2011). *Towards an Understanding of Tree Diversity*. PhD Thesis Utrecht University. ISBN:9789039355268.

Stropp, J., Ter Steege, H., & Malhi, Y. (2009). Disentangling regional and local tree diversity in the Amazon. *Ecography*, *32*(1), 46–54. https://doi.org/10.1111/j.1600-0587.2009.05811.x

Sullivan, M. J. P. P., Talbot, J., Lewis, S. L., Phillips, O. L., Qie, L., Begne, S. K., Chave, J., Cuni-Sanchez, A., Hubau, W., Lopez-Gonzalez, G., Miles, L., Monteagudo-Mendoza, A., Sonké, B., Sunderland, T., ter Steege, H., White, L. J. T. T., Affum-Baffoe, K., Aiba, S., de Almeida, E. C., … Zemagho, L. (2017). Diversity and carbon storage across the tropical forest biome. *Scientific Reports*, *7*(1), 39102. https://doi.org/10.1038/srep39102

ter Steege, H., Henkel, T. W., Helal, N., Marimon, B. S., Marimon-Junior, B. H., Huth, A., Groeneveld, J., Sabatier, D., Coelho, L. de S., Filho, D. de A. L., Salomão, R. P., Amaral, I. L., Matos, F. D. de A., Castilho, C. V., Phillips, O. L., Guevara, J. E., Carim, M. de J. V., Cárdenas López, D., Magnusson, W. E., … Melgaço, K. (2019). Rarity of monodominance in hyperdiverse Amazonian forests. *Scientific Reports*, *9*(1), 13822. https://doi.org/10.1038/s41598-019-50323-9

ter Steege, H., Mota de Oliveira, S., Pitman, N. C. A., Sabatier, D., Antonelli, A., Guevara Andino, J. E., Aymard, G. A., & Salomão, R. P. (2019). Towards a dynamic list of Amazonian tree species. *Scientific Reports*, *9*(1), 3501. https://doi.org/10.1038/s41598-019-40101-y

ter Steege, H., Pitman, N. C. A., Sabatier, D., Baraloto, C., Salomão, R. P., Guevara, J. E., Phillips, O. L., Castilho, C. V., Magnusson, W. E., Molino, J.-F. F., Monteagudo, A., Núñez Vargas, P., Montero, J. C., Feldpausch, T. R., Coronado, E. N. H. H., Killeen, T. J., Mostacedo, B., Vasquez, R., Assis, R. L., … Silman, M. R. (2013). Hyperdominance in the Amazonian tree flora. *Science*, *342*(6156), 1243092. https://doi.org/10.1126/science.1243092

ter Steege, H., & Zondervan, G. (2000). A preliminary analysis of large-scale forest inventory data of the Guiana Shield. In H. ter Steege (Ed.), *Plant diversity in Guyana. With recommendation for a protected areas strategy.* (Tropenbos, pp. 35–54). Tropenbos Foundation, Wageningen. https://doi.org/10.1007/s117-002-8112-z

Wickham, H. (2016). *ggplot2: Elegant Graphics for Data Analysis*. Springer-Verlag New York.

Wickham, H. (2019). *stringr: Simple, Consistent Wrappers for Common String Operations* (R package version 1.4.0.). https://cran.r-project.org/package=stringr

Zuur, A. F., Ieno, E. N., & Elphick, C. S. (2010). A protocol for data exploration to avoid common statistical problems. *Methods in Ecology and Evolution*, *1*(1), 3–14. https://doi.org/10.1111/j.2041-210X.2009.00001.x

#### Table S1.1. List of commercially relevant timber tree species in the Guiana Shield region

| **Timber tree species** | **References** |
| --- | --- |
| Abarema jupunba | 1, 3, 5, 6, 7, 8 |
| Acioa longipendula | 5, 8 |
| Acosmium cardenasii | 5 |
| Acrocomia aculeata | 5, 8 |
| Agonandra brasiliensis | 5, 8 |
| Aiouea montana | 5, 8 |
| Albizia niopoides | 5, 8 |
| Alchornea triplinervia | 5 |
| Alchorneopsis floribunda | 5, 7 |
| Aldina insignis | 5 |
| Alexa imperatricis | 3, 5, 8 |
| Alexa wachenheimii | 5, 7 |
| Allantoma decandra | 2, 5 |
| Amanoa guianensis | 5 |
| Ampelocera ruizii | 5 |
| Amphiodon effusus | 5, 8 |
| Anacardium giganteum | 5, 8 |
| Anacardium parvifolium | 5, 8 |
| Anacardium spruceanum | 1, 2, 5, 6, 8 |
| Anadenanthera colubrina | 2, 5, 8 |
| Anadenanthera peregrina | 5, 8 |
| Andira coriacea | 5, 7, 8 |
| Andira fraxinifolia | 5, 8 |
| Andira inermis | 1, 3, 5, 6, 7, 8 |
| Andira parviflora | 5, 8 |
| Andira surinamensis | 1, 3, 5, 6, 7, 8 |
| Aniba canelilla | 5, 8 |
| Aniba citrifolia | 5, 8 |
| Aniba guianensis | 5, 8 |
| Aniba hostmanniana | 5, 7, 8 |
| Aniba hypoglauca | 3, 5 |
| Aniba kappleri | 5, 7, 8 |
| Aniba megaphylla | 5, 8 |
| Aniba panurensis | 1, 5, 6, 8 |
| Aniba parviflora | 5, 8 |
| Aniba rosiodora | 1, 5, 6, 8 |
| Aniba terminalis | 5, 8 |
| Aniba williamsii | 5, 8 |
| Antonia ovata | 1, 3, 5, 6 |
| Apeiba albiflora | 5, 8 |
| Apeiba glabra | 5, 8 |
| Apeiba petoumo | 2, 5, 8 |
| Apuleia leiocarpa | 2, 5, 8 |
| Aspidosperma album | 1, 3, 5, 6, 7, 8 |
| Aspidosperma carapanauba | 5, 8 |
| Aspidosperma cylindrocarpon | 5 |
| Aspidosperma desmanthum | 2, 3, 5, 7, 8 |
| Aspidosperma discolor | 5, 8 |
| Aspidosperma excelsum | 5, 7, 8 |
| Aspidosperma helstonei | 5, 7 |
| Aspidosperma megalocarpon | 5, 8 |
| Aspidosperma parvifolium | 3, 5 |
| Aspidosperma sandwithianum | 5, 7, 8 |
| Aspidosperma spruceanum | 5, 8 |
| Aspidosperma tomentosum | 5 |
| Astronium graveolens | 2, 5, 8 |
| Astronium lecointei | 2, 5, 7, 8 |
| Astronium ulei | 2, 3, 5, 8 |
| Bagassa guianensis | 1, 2, 3, 4, 5, 6, 7, 8 |
| Balizia pedicellaris | 2, 4, 5, 8 |
| Barnebydendron riedelii | 5 |
| Batesia floribunda | 5 |
| Batocarpus amazonicus | 5 |
| Bocoa prouacensis | 1, 4, 5, 6, 7 |
| Bowdichia virgilioides | 2, 5, 8 |
| Brosimum acutifolium | 2, 4, 5, 7, 8 |
| Brosimum alicastrum | 5 |
| Brosimum guianense | 1, 4, 5, 6, 7, 8 |
| Brosimum lactescens | 5, 8 |
| Brosimum parinarioides | 2, 4, 5, 7, 8 |
| Brosimum potabile | 2, 5, 8 |
| Brosimum rubescens | 1, 2, 4, 5, 6, 7, 8 |
| Brosimum utile | 4, 5, 8 |
| Byrsonima aerugo | 5, 8 |
| Byrsonima crassifolia | 5, 8 |
| Byrsonima crispa | 5, 8 |
| Byrsonima densa | 5, 8 |
| Byrsonima laevigata | 5 |
| Byrsonima stipulacea | 5, 8 |
| Calatola costaricensis | 5 |
| Calliandra laxa | 5, 8 |
| Calophyllum brasiliense | 2, 3, 5, 7, 8 |
| Calycophyllum megistocaulum | 5, 8 |
| Candolleodendron brachystachyum | 5 |
| Capirona decorticans | 5 |
| Caraipa densifolia | 2, 5, 7, 8 |
| Caraipa punctulata | 5, 8 |
| Caraipa racemosa | 5 |
| Caraipa richardiana | 5, 7, 8 |
| Carapa guianensis | 1, 2, 3, 4, 5, 6, 7, 8 |
| Carapa surinamensis | 1, 3, 4, 5, 6 |
| Cariniana estrellensis | 5 |
| Cariniana ianeirensis | 5 |
| Cariniana micrantha | 2, 5, 8 |
| Caryocar glabrum | 2, 4, 5, 8 |
| Caryocar microcarpum | 4, 5, 8 |
| Caryocar nuciferum | 1, 5, 6 |
| Caryocar villosum | 2, 5, 8 |
| Casearia arborea | 5, 8 |
| Casearia commersoniana | 5, 8 |
| Casearia decandra | 5, 8 |
| Casearia grandiflora | 5, 8 |
| Casearia javitensis | 5, 8 |
| Casearia negrensis | 5, 8 |
| Casearia pitumba | 5, 8 |
| Casearia spinescens | 5, 8 |
| Casearia sylvestris | 5, 8 |
| Cassia spruceana | 5 |
| Catostemma altsonii | 3, 5 |
| Catostemma commune | 3, 5 |
| Catostemma fragrans | 3, 5 |
| Cecropia sciadophylla | 5 |
| Cedrela fissilis | 2, 5, 8 |
| Cedrela odorata | 1, 2, 3, 4, 5, 6, 7, 8 |
| Cedrelinga cateniformis | 1, 2, 5, 6, 7, 8 |
| Ceiba pentandra | 2, 5, 8 |
| Ceiba samauma | 5 |
| Centrolobium microchaete | 5 |
| Chaetocarpus schomburgkianus | 5, 7 |
| Chamaecrista adiantifolia | 5, 8 |
| Chamaecrista apoucouita | 5 |
| Chaunochiton kappleri | 5, 7 |
| Chimarrhis barbata | 5, 8 |
| Chimarrhis turbinata | 5, 8 |
| Chlorocardium rodiei | 3, 5 |
| Chromolucuma rubriflora | 5, 8 |
| Chrysophyllum argenteum | 5, 8 |
| Chrysophyllum cuneifolium | 5, 7, 8 |
| Chrysophyllum eximium | 5, 8 |
| Chrysophyllum gonocarpum | 5 |
| Chrysophyllum lucentifolium | 2, 5 |
| Chrysophyllum pomiferum | 3, 4, 5, 7, 8 |
| Chrysophyllum prieurii | 5, 8 |
| Chrysophyllum sanguinolentum | 4, 5, 8 |
| Chrysophyllum sparsiflorum | 5, 8 |
| Chrysophyllum venezuelanense | 5, 8 |
| Citharexylum spinosum | 5, 8 |
| Clarisia racemosa | 2, 5, 8 |
| Clathrotropis brachypetala | 3, 5 |
| Clathrotropis macrocarpa | 3, 5, 8 |
| Copaifera guyanensis | 1, 5, 6, 8 |
| Copaifera martii | 5, 8 |
| Cordia alliodora | 1, 5, 6, 8 |
| Cordia bicolor | 2, 5, 8 |
| Cordia exaltata | 5, 8 |
| Cordia fallax | 5, 8 |
| Cordia goeldiana | 2, 5, 8 |
| Cordia laevifrons | 5, 8 |
| Cordia nodosa | 5, 8 |
| Cordia panicularis | 5, 8 |
| Cordia sagotii | 2, 5, 8 |
| Cordia tetrandra | 5, 8 |
| Couepia bracteosa | 5, 8 |
| Couepia caryophylloides | 1, 5, 6 |
| Couepia guianensis | 5, 8 |
| Couepia joaquinae | 5, 8 |
| Couepia magnoliifolia | 5, 8 |
| Couepia robusta | 5, 8 |
| Couma guianensis | 4, 5, 7, 8 |
| Couma macrocarpa | 5, 8 |
| Couma utilis | 5, 8 |
| Couratari gloriosa | 3, 5 |
| Couratari guianensis | 1, 2, 3, 5, 6, 8 |
| Couratari multiflora | 2, 3, 5, 8 |
| Couratari oblongifolia | 2, 5, 7, 8 |
| Couratari oligantha | 5, 8 |
| Couratari stellata | 2, 5, 7, 8 |
| Crudia bracteata | 5, 8 |
| Crudia glaberrima | 5, 8 |
| Cupania diphylla | 5, 8 |
| Cupania hirsuta | 5, 8 |
| Cupania scrobiculata | 5, 8 |
| Cyclolobium brasiliense | 5 |
| Dacryodes nitens | 5 |
| Dalbergia ecastaphyllum | 5, 8 |
| Dendrobangia boliviana | 4, 5 |
| Dendropanax arboreus | 5 |
| Dialium guianense | 5, 8 |
| Dicorynia guianensis | 1, 4, 5, 6, 7 |
| Didymopanax decaphyllus | 3, 5, 7, 8 |
| Didymopanax morototoni | 1, 2, 3, 5, 6, 7, 8 |
| Dimorphandra polyandra | 3, 5, 8 |
| Dinizia excelsa | 2, 5, 8 |
| Diospyros capreifolia | 5, 8 |
| Diospyros carbonaria | 5, 8 |
| Diospyros cayennensis | 5, 8 |
| Diploon cuspidatum | 5, 8 |
| Diplotropis purpurea | 1, 2, 3, 4, 5, 6, 7, 8 |
| Diplotropis triloba | 5 |
| Dipteryx magnifica | 2, 5, 8 |
| Dipteryx odorata | 1, 2, 3, 4, 5, 6, 7, 8 |
| Dipteryx punctata | 1, 4, 5, 6, 7, 8 |
| Drypetes variabilis | 1, 5, 6, 7, 8 |
| Duroia eriopila | 5 |
| Duroia longiflora | 5 |
| Ecclinusa guianensis | 5 |
| Ecclinusa lanceolata | 5, 8 |
| Ecclinusa ramiflora | 5, 8 |
| Emmotum fagifolium | 5, 8 |
| Emmotum nitens | 5, 8 |
| Endopleura uchi | 5, 8 |
| Enterolobium oldemanii | 4, 5 |
| Enterolobium schomburgkii | 2, 4, 5, 7, 8 |
| Eperua falcata | 1, 3, 4, 5, 6, 7, 8 |
| Eperua grandiflora | 1, 3, 4, 5, 6 |
| Eperua jenmanii | 3, 5 |
| Eperua rubiginosa | 1, 3, 4, 5, 6 |
| Eriotheca crassa | 5, 7, 8 |
| Eriotheca globosa | 5, 8 |
| Eriotheca longitubulosa | 5 |
| Eriotheca surinamensis | 5, 8 |
| Erisma calcaratum | 5, 8 |
| Erisma uncinatum | 1, 2, 5, 6, 7, 8 |
| Eschweilera alata | 3, 5 |
| Eschweilera albiflora | 5, 8 |
| Eschweilera apiculata | 5, 8 |
| Eschweilera atropetiolata | 5, 8 |
| Eschweilera collina | 5, 8 |
| Eschweilera coriacea | 1, 2, 3, 5, 6, 8 |
| Eschweilera decolorans | 3, 5 |
| Eschweilera grandiflora | 5, 8 |
| Eschweilera micrantha | 5, 8 |
| Eschweilera ovata | 5, 8 |
| Eschweilera parviflora | 3, 5, 8 |
| Eschweilera pedicellata | 3, 5, 7, 8 |
| Eschweilera rhododendrifolia | 5, 8 |
| Eschweilera sagotiana | 3, 5, 8 |
| Eschweilera subglandulosa | 3, 5, 7, 8 |
| Eschweilera truncata | 5, 8 |
| Eschweilera wachenheimii | 3, 5 |
| Eugenia coffeifolia | 5, 8 |
| Eugenia cupulata | 5, 8 |
| Eugenia florida | 5, 8 |
| Eugenia lambertiana | 5, 8 |
| Eugenia moschata | 5, 8 |
| Eugenia patrisii | 5, 8 |
| Eugenia protenta | 5, 8 |
| Eugenia punicifolia | 5, 8 |
| Eugenia stictopetala | 5, 8 |
| Eugenia wentii | 5, 8 |
| Eugenia wullschlaegeliana | 5, 8 |
| Euplassa pinnata | 5, 8 |
| Exellodendron barbatum | 5, 8 |
| Ficus americana | 5, 8 |
| Ficus boliviana | 5 |
| Ficus coerulescens | 5 |
| Ficus gomelleira | 5, 8 |
| Ficus insipida | 5, 8 |
| Ficus maxima | 5, 8 |
| Ficus nymphaeifolia | 5, 8 |
| Ficus paraensis | 5, 8 |
| Ficus pertusa | 5, 8 |
| Garcinia benthamiana | 1, 5, 6 |
| Garcinia macrophylla | 1, 5, 6 |
| Garcinia madruno | 1, 5, 6 |
| Gaulettia elata | 5, 8 |
| Geissospermum laeve | 5, 8 |
| Geissospermum sericeum | 5, 8 |
| Genipa americana | 5 |
| Glycydendron amazonicum | 4, 5 |
| Goupia glabra | 1, 2, 3, 4, 5, 6, 7, 8 |
| Guarea gomma | 5, 8 |
| Guarea guidonia | 5, 8 |
| Guarea kunthiana | 5, 8 |
| Guarea macrophylla | 5, 8 |
| Guarea pubescens | 5, 8 |
| Guarea scabra | 5, 8 |
| Guarea silvatica | 5, 8 |
| Guarea trunciflora | 5, 8 |
| Guatteria megalophylla | 5, 8 |
| Guatteria punctata | 5, 8 |
| Guatteria schomburgkiana | 5, 8 |
| Guazuma ulmifolia | 5, 8 |
| Guianodendron praeclarum | 3, 5 |
| Gustavia augusta | 5, 8 |
| Gustavia hexapetala | 5, 8 |
| Gustavia poeppigiana | 5, 8 |
| Handroanthus capitatus | 1, 5, 6, 7, 8 |
| Handroanthus impetiginosus | 2, 4, 5, 8 |
| Handroanthus incanus | 2, 5 |
| Handroanthus ochraceus | 2, 5, 8 |
| Handroanthus serratifolius | 1, 2, 3, 4, 5, 6, 7, 8 |
| Hebepetalum humiriifolium | 5 |
| Heisteria ovata | 5 |
| Helicostylis pedunculata | 5, 8 |
| Helicostylis scabra | 5, 8 |
| Helicostylis tomentosa | 5, 8 |
| Heliocarpus americanus | 5 |
| Hernandia guianensis | 5, 8 |
| Hevea benthamiana | 5, 8 |
| Hevea guianensis | 5, 8 |
| Hevea pauciflora | 5, 8 |
| Hieronyma alchorneoides | 3, 5, 7, 8 |
| Himatanthus articulatus | 5, 8 |
| Himatanthus bracteatus | 5 |
| Himatanthus sucuuba | 5, 8 |
| Hirtella bicornis | 5, 8 |
| Hirtella glandulosa | 5, 8 |
| Hirtella macrosepala | 5, 7 |
| Hirtella obidensis | 5, 8 |
| Hirtella triandra | 5, 8 |
| Homalolepis cedron | 5, 8 |
| Homalolepis morettii | 5 |
| Huberodendron swietenioides | 5 |
| Humiria balsamifera | 1, 3, 4, 5, 6, 7 |
| Humiriastrum cuspidatum | 5, 8 |
| Humiriastrum obovatum | 5 |
| Humiriastrum subcrenatum | 5 |
| Hura crepitans | 2, 5, 8 |
| Hydrochorea corymbosa | 1, 5, 6, 7, 8 |
| Hydrochorea gonggrijpii | 5, 7 |
| Hymenaea courbaril | 1, 2, 3, 4, 5, 6, 7, 8 |
| Hymenaea intermedia | 5, 8 |
| Hymenolobium excelsum | 2, 5, 8 |
| Hymenolobium flavum | 1, 3, 5, 6, 7, 8 |
| Hymenolobium heterocarpum | 2, 5, 8 |
| Hymenolobium modestum | 2, 5, 8 |
| Hymenolobium petraeum | 2, 5, 8 |
| Hymenolobium pulcherrimum | 2, 5, 8 |
| Hymenolobium sericeum | 2, 5, 8 |
| Hymenopus heteromorphus | 1, 5, 6, 8 |
| Hymenopus laevigatus | 5, 8 |
| Hymenopus latifolius | 5, 8 |
| Hymenopus macrophyllus | 5, 8 |
| Ilex inundata | 5 |
| Inga acrocephala | 5, 8 |
| Inga alba | 1, 3, 4, 5, 6, 7, 8 |
| Inga auristellae | 5, 8 |
| Inga bourgonii | 5 |
| Inga brachystachys | 5, 8 |
| Inga capitata | 5, 8 |
| Inga cayennensis | 5, 8 |
| Inga cinnamomea | 5, 8 |
| Inga cordatoalata | 5, 8 |
| Inga cylindrica | 5, 8 |
| Inga disticha | 5, 8 |
| Inga edulis | 5, 8 |
| Inga flagelliformis | 5, 8 |
| Inga gracilifolia | 5, 8 |
| Inga heterophylla | 5, 8 |
| Inga huberi | 5, 8 |
| Inga ingoides | 5, 8 |
| Inga lateriflora | 5, 8 |
| Inga laurina | 5, 8 |
| Inga leiocalycina | 5, 8 |
| Inga longiflora | 5, 8 |
| Inga macrophylla | 5, 8 |
| Inga marginata | 5, 8 |
| Inga obidensis | 5, 8 |
| Inga paraensis | 5, 8 |
| Inga pezizifera | 5, 8 |
| Inga rubiginosa | 5, 8 |
| Inga sertulifera | 5, 8 |
| Inga splendens | 5, 8 |
| Inga stipularis | 5, 8 |
| Inga thibaudiana | 5, 8 |
| Inga umbellifera | 5, 8 |
| Inga umbratica | 5, 8 |
| Inga vera | 5, 8 |
| Iryanthera crassifolia | 5, 8 |
| Iryanthera elliptica | 5, 8 |
| Iryanthera hostmannii | 5, 8 |
| Iryanthera juruensis | 5, 8 |
| Iryanthera laevis | 5, 8 |
| Iryanthera lancifolia | 3, 5, 8 |
| Iryanthera olacoides | 5, 8 |
| Iryanthera paradoxa | 5, 8 |
| Iryanthera paraensis | 5, 8 |
| Iryanthera sagotiana | 5, 8 |
| Jacaranda copaia | 1, 2, 3, 4, 5, 6, 7, 8 |
| Jacaratia spinosa | 5 |
| Kubitzkia mezii | 5, 7, 8 |
| Lacmellea aculeata | 5 |
| Lacunaria jenmanii | 5 |
| Laetia procera | 1, 2, 3, 4, 5, 6, 7, 8 |
| Laplacea fruticosa | 5, 7 |
| Lecointea amazonica | 5, 8 |
| Lecythis chartacea | 5, 8 |
| Lecythis confertiflora | 3, 5 |
| Lecythis congestiflora | 5, 7 |
| Lecythis corrugata | 1, 3, 5, 6, 8 |
| Lecythis holcogyne | 5 |
| Lecythis idatimon | 1, 5, 6, 8 |
| Lecythis persistens | 5 |
| Lecythis poiteaui | 5, 7, 8 |
| Lecythis prancei | 5 |
| Lecythis retusa | 5, 8 |
| Lecythis zabucajo | 3, 5, 7, 8 |
| Leptobalanus apetalus | 5, 7, 8 |
| Leptobalanus longistylus | 5, 8 |
| Leptobalanus octandrus | 5, 8 |
| Leptobalanus sclerophyllus | 5, 8 |
| Leptolobium nitens | 5, 8 |
| Licania affinis | 5, 8 |
| Licania alba | 3, 5, 8 |
| Licania bracteata | 5, 8 |
| Licania buxifolia | 5 |
| Licania canescens | 5, 8 |
| Licania cuprea | 5 |
| Licania densiflora | 5, 7, 8 |
| Licania hypoleuca | 5, 8 |
| Licania incana | 5, 8 |
| Licania kunthiana | 5, 8 |
| Licania laxiflora | 3, 5, 7 |
| Licania leptostachya | 5, 8 |
| Licania majuscula | 1, 3, 5, 6 |
| Licania membranacea | 5, 8 |
| Licania micrantha | 5, 8 |
| Licania pallida | 5, 8 |
| Licania robusta | 5, 8 |
| Licaria canella | 1, 5, 6, 8 |
| Licaria cannella | 1, 3, 5, 6, 7, 8 |
| Licaria chrysophylla | 5, 8 |
| Licaria crassifolia | 5 |
| Licaria guianensis | 5, 8 |
| Licaria martiniana | 5 |
| Licaria pachycarpa | 5, 8 |
| Licaria triandra | 5 |
| Lindackeria paludosa | 5, 8 |
| Lonchocarpus nicou | 5, 7 |
| Lonchocarpus sericeus | 5, 8 |
| Loxopterygium sagotii | 1, 3, 5, 6, 7 |
| Luehea grandiflora | 5 |
| Luehea speciosa | 5, 8 |
| Lueheopsis rosea | 1, 5, 6 |
| Lueheopsis rugosa | 5 |
| Mabea piriri | 5 |
| Machaerium nyctitans | 5 |
| Machaerium villosum | 5 |
| Maclura tinctoria | 5, 8 |
| Macoubea guianensis | 5, 7, 8 |
| Macrolobium acaciifolium | 5, 8 |
| Macrolobium angustifolium | 5, 8 |
| Macrolobium bifolium | 5, 8 |
| Macrolobium campestre | 5, 8 |
| Macrolobium multijugum | 5, 8 |
| Macrolobium pendulum | 5, 8 |
| Mahurea palustris | 5 |
| Malouetia tamaquarina | 5, 8 |
| Manilkara bidentata | 1, 2, 3, 4, 5, 6, 7, 8 |
| Manilkara elata | 2, 4, 5, 7, 8 |
| Manilkara paraensis | 5, 8 |
| Maprounea guianensis | 5 |
| Maquira calophylla | 5, 8 |
| Maquira guianensis | 5, 8 |
| Maquira sclerophylla | 2, 5, 8 |
| Martiodendron parviflorum | 1, 5, 6, 7, 8 |
| Matayba opaca | 5 |
| Maytenus guyanensis | 5, 8 |
| Melicoccus pedicellaris | 5, 7 |
| Mezilaurus itauba | 2, 5, 8 |
| Mezilaurus synandra | 5 |
| Miconia mirabilis | 5 |
| Miconia tschudyoides | 5 |
| Micrandra elata | 5, 8 |
| Micrandra rossiana | 5, 8 |
| Micropholis acutangula | 5, 8 |
| Micropholis egensis | 4, 5, 7, 8 |
| Micropholis guyanensis | 1, 5, 6, 8 |
| Micropholis melinoniana | 2, 4, 5, 8 |
| Micropholis mensalis | 5, 8 |
| Micropholis obscura | 5 |
| Micropholis trunciflora | 5, 8 |
| Micropholis venulosa | 5, 7, 8 |
| Micropholis williamii | 5, 8 |
| Minquartia guianensis | 5, 7, 8 |
| Monopteryx inpae | 4, 5 |
| Monteverdia myrsinoides | 5, 8 |
| Monteverdia pruinosa | 5, 8 |
| Moquilea egleri | 5, 8 |
| Moquilea guianensis | 5, 8 |
| Moquilea minutiflora | 5, 8 |
| Moquilea unguiculata | 5, 8 |
| Mora excelsa | 1, 3, 5, 6, 7 |
| Mora gonggrijpii | 3, 5, 7, 8 |
| Moronobea coccinea | 3, 4, 5, 8 |
| Mouriri collocarpa | 5, 8 |
| Mouriri crassifolia | 5, 8 |
| Mouriri duckeana | 5, 8 |
| Mouriri francavillana | 5, 8 |
| Mouriri grandiflora | 5, 8 |
| Mouriri huberi | 5, 8 |
| Mouriri sagotiana | 5, 8 |
| Myrcia guianensis | 5, 8 |
| Myrciaria floribunda | 5, 8 |
| Myroxylon balsamum | 5 |
| Naucleopsis oblongifolia | 5, 8 |
| Nectandra cissiflora | 5, 8 |
| Nectandra cuspidata | 5, 8 |
| Nectandra globosa | 5, 8 |
| Nectandra lanceolata | 5, 8 |
| Neea floribunda | 5 |
| Ocotea aciphylla | 5, 8 |
| Ocotea amazonica | 5, 8 |
| Ocotea argyrophylla | 5 |
| Ocotea aurantiodora | 5, 8 |
| Ocotea canaliculata | 3, 5, 8 |
| Ocotea cernua | 5, 8 |
| Ocotea cujumary | 5, 8 |
| Ocotea douradensis | 5, 8 |
| Ocotea floribunda | 3, 5, 7 |
| Ocotea glomerata | 1, 3, 5, 6, 7, 8 |
| Ocotea guianensis | 1, 5, 6, 7, 8 |
| Ocotea leucoxylon | 5, 8 |
| Ocotea oblonga | 3, 5 |
| Ocotea percurrens | 1, 5, 6 |
| Ocotea petalanthera | 5, 7, 8 |
| Ocotea puberula | 5, 7, 8 |
| Ocotea splendens | 5, 7, 8 |
| Ocotea tomentella | 3, 5, 8 |
| Ormosia arborea | 5 |
| Ormosia coarctata | 5 |
| Ormosia coccinea | 1, 3, 5, 6, 7, 8 |
| Ormosia coutinhoi | 3, 5, 8 |
| Ormosia discolor | 5, 8 |
| Ormosia flava | 5, 8 |
| Ormosia nobilis | 5, 8 |
| Ormosia paraensis | 5, 7, 8 |
| Osteophloeum platyspermum | 2, 5, 8 |
| Otoba parvifolia | 5 |
| Oxandra asbeckii | 5 |
| Pachira aquatica | 5, 8 |
| Pachira coriacea | 5, 8 |
| Pachira dolichocalyx | 5 |
| Pachira flaviflora | 5 |
| Pachira insignis | 5, 8 |
| Pachira nervosa | 5, 7 |
| Panopsis sessilifolia | 5, 8 |
| Parahancornia fasciculata | 2, 3, 5, 8 |
| Paramachaerium ormosioides | 4, 5 |
| Parinari campestris | 1, 3, 5, 6, 7, 8 |
| Parinari excelsa | 5, 8 |
| Parinari montana | 5, 8 |
| Parinari parvifolia | 5 |
| Parinari rodolphii | 3, 5, 8 |
| Parinariopsis licaniiflora | 5, 8 |
| Parkia decussata | 5, 8 |
| Parkia igneiflora | 5, 8 |
| Parkia multijuga | 2, 5, 8 |
| Parkia nitida | 1, 5, 6, 7, 8 |
| Parkia pendula | 1, 2, 4, 5, 6, 7, 8 |
| Parkia reticulata | 5, 8 |
| Parkia ulei | 5, 7, 8 |
| Parkia velutina | 5, 8 |
| Peltogyne catingae | 5, 8 |
| Peltogyne floribunda | 5, 8 |
| Peltogyne lecointei | 5, 8 |
| Peltogyne paniculata | 1, 5, 6, 8 |
| Peltogyne venosa | 1, 3, 5, 6, 7, 8 |
| Pentaclethra macroloba | 5, 8 |
| Pera glabrata | 5 |
| Perebea guianensis | 5, 8 |
| Perebea mollis | 5, 8 |
| Perebea rubra | 5, 8 |
| Phyllostylon rhamnoides | 5 |
| Platonia insignis | 1, 3, 4, 5, 6, 7, 8 |
| Platymiscium pinnatum | 1, 5, 6, 7, 8 |
| Platymiscium trinitatis | 2, 5, 8 |
| Pogonophora schomburgkiana | 5, 8 |
| Poraqueiba guianensis | 5, 8 |
| Pourouma bicolor | 5, 8 |
| Pourouma cecropiifolia | 5, 8 |
| Pourouma guianensis | 5, 8 |
| Pourouma melinonii | 5, 8 |
| Pourouma mollis | 5, 8 |
| Pourouma velutina | 5, 8 |
| Pouteria ambelaniifolia | 5, 8 |
| Pouteria anomala | 5, 8 |
| Pouteria bangii | 5, 8 |
| Pouteria bilocularis | 5, 8 |
| Pouteria caimito | 5, 8 |
| Pouteria campanulata | 5, 8 |
| Pouteria cladantha | 5, 8 |
| Pouteria coriacea | 5, 8 |
| Pouteria cuspidata | 1, 3, 5, 6, 8 |
| Pouteria decorticans | 5, 8 |
| Pouteria egregia | 5, 8 |
| Pouteria elegans | 5, 8 |
| Pouteria engleri | 5, 8 |
| Pouteria eugeniifolia | 5, 8 |
| Pouteria flavilatex | 5 |
| Pouteria glomerata | 5, 8 |
| Pouteria gongrijpii | 5, 8 |
| Pouteria guianensis | 3, 5, 8 |
| Pouteria hispida | 5, 8 |
| Pouteria jariensis | 5, 8 |
| Pouteria laevigata | 4, 5 |
| Pouteria macrocarpa | 5, 8 |
| Pouteria manaosensis | 5, 8 |
| Pouteria melanopoda | 5, 8 |
| Pouteria nemorosa | 5 |
| Pouteria opposita | 5 |
| Pouteria platyphylla | 5 |
| Pouteria reticulata | 5, 8 |
| Pouteria retinervis | 5, 8 |
| Pouteria rodriguesiana | 4, 5 |
| Pouteria sagotiana | 5, 8 |
| Pouteria singularis | 5 |
| Pouteria speciosa | 3, 5, 8 |
| Pouteria torta | 5 |
| Pouteria venosa | 5, 8 |
| Pouteria virescens | 5, 8 |
| Pradosia cochlearia | 5, 8 |
| Pradosia ptychandra | 1, 5, 6, 7 |
| Pradosia schomburgkiana | 5 |
| Pradosia surinamensis | 1, 5, 6, 7, 8 |
| Pradosia verticillata | 5, 8 |
| Protium altissimum | 1, 3, 5, 6, 8 |
| Protium altsonii | 5, 8 |
| Protium apiculatum | 5, 8 |
| Protium aracouchini | 5, 8 |
| Protium crenatum | 5, 8 |
| Protium cuneatum | 5, 8 |
| Protium decandrum | 1, 3, 5, 6, 8 |
| Protium giganteum | 5, 8 |
| Protium guianense | 5, 8 |
| Protium hebetatum | 5, 8 |
| Protium heptaphyllum | 2, 5, 8 |
| Protium morii | 5, 8 |
| Protium nitidifolium | 5, 8 |
| Protium opacum | 5, 8 |
| Protium pallidum | 5, 8 |
| Protium paniculatum | 5, 8 |
| Protium pilosum | 5, 8 |
| Protium polybotryum | 1, 5, 6, 8 |
| Protium robustum | 5, 8 |
| Protium sagotianum | 5, 7, 8 |
| Protium spruceanum | 5, 8 |
| Protium stevensonii | 5, 7, 8 |
| Protium strumosum | 5, 8 |
| Protium subserratum | 5, 8 |
| Protium surinamense | 1, 5, 6, 8 |
| Protium tenuifolium | 1, 5, 6, 8 |
| Protium trifoliolatum | 5, 8 |
| Protium unifoliolatum | 5, 8 |
| Pseudolmedia laevis | 5 |
| Pseudopiptadenia psilostachya | 4, 5, 8 |
| Pseudopiptadenia suaveolens | 1, 2, 4, 5, 6, 7 |
| Pterocarpus officinalis | 5, 8 |
| Pterocarpus rohrii | 2, 3, 5, 8 |
| Pterocarpus santalinoides | 5, 8 |
| Qualea coerulea | 1, 5, 6, 7, 8 |
| Qualea dinizii | 1, 2, 5, 6, 7, 8 |
| Qualea paraensis | 2, 5, 8 |
| Qualea rosea | 1, 4, 5, 6, 7 |
| Qualea tessmannii | 5 |
| Rauvolfia paraensis | 5, 8 |
| Rauvolfia pentaphylla | 5, 8 |
| Recordoxylon speciosum | 4, 5 |
| Rhodostemonodaphne grandis | 1, 5, 6 |
| Rhodostemonodaphne morii | 5 |
| Roupala montana | 2, 5, 8 |
| Ruizterania albiflora | 1, 4, 5, 6, 7, 8 |
| Ruizterania cassiquiarensis | 5, 8 |
| Sacoglottis cydonioides | 5, 7 |
| Sacoglottis guianensis | 1, 3, 5, 6, 7, 8 |
| Samanea saman | 5, 8 |
| Sapindus saponaria | 5 |
| Sapium ciliatum | 5, 8 |
| Sapium glandulosum | 5, 8 |
| Scleronema micranthum | 5, 8 |
| Senegalia bonariensis | 5 |
| Senna multijuga | 5, 8 |
| Sextonia rubra | 1, 2, 3, 4, 5, 6, 7, 8 |
| Simaba guianensis | 5, 8 |
| Simaba orinocensis | 5, 8 |
| Simarouba amara | 1, 2, 3, 4, 5, 6, 7, 8 |
| Sloanea brevipes | 5, 8 |
| Sloanea eichleri | 5, 8 |
| Sloanea fendleriana | 5, 8 |
| Sloanea floribunda | 5, 8 |
| Sloanea garckeana | 5, 8 |
| Sloanea grandiflora | 5, 8 |
| Sloanea guianensis | 5, 8 |
| Sloanea laurifolia | 5, 8 |
| Sloanea nitida | 5, 8 |
| Sloanea obtusifolia | 5, 8 |
| Spondias mombin | 2, 5, 8 |
| Stenostomum acreanum | 5 |
| Sterculia excelsa | 2, 5, 8 |
| Sterculia multiovula | 5 |
| Sterculia pruriens | 1, 3, 5, 6, 7, 8 |
| Sterculia rugosa | 3, 5 |
| Sterculia villifera | 5, 7 |
| Stryphnodendron adstringens | 5 |
| Stryphnodendron guianense | 5, 8 |
| Stryphnodendron paniculatum | 5, 8 |
| Stryphnodendron polystachyum | 5, 7, 8 |
| Stryphnodendron pulcherrimum | 5, 8 |
| Swartzia aptera | 5, 8 |
| Swartzia arborescens | 5, 8 |
| Swartzia benthamiana | 3, 5 |
| Swartzia brachyrachis | 5, 8 |
| Swartzia cardiosperma | 5, 8 |
| Swartzia corrugata | 5, 8 |
| Swartzia grandifolia | 5, 8 |
| Swartzia guianensis | 5, 7 |
| Swartzia laevicarpa | 5, 8 |
| Swartzia leiocalycina | 3, 5 |
| Swartzia oblanceolata | 5 |
| Swartzia panacoco | 4, 5 |
| Swartzia polyphylla | 5, 8 |
| Swartzia recurva | 5, 8 |
| Swartzia schomburgkii | 5 |
| Swartzia sprucei | 3, 5 |
| Swartzia ulei | 5, 8 |
| Symphonia globulifera | 1, 2, 3, 4, 5, 6, 7, 8 |
| Tabebuia insignis | 3, 5, 8 |
| Tachigali chrysophylla | 5, 8 |
| Tachigali glauca | 2, 5, 8 |
| Tachigali guianensis | 3, 5, 7, 8 |
| Tachigali melanocarpa | 5, 8 |
| Tachigali melinonii | 4, 5, 7, 8 |
| Tachigali micropetala | 3, 5, 8 |
| Tachigali paniculata | 5, 8 |
| Tachigali paraensis | 5, 7, 8 |
| Tachigali richardiana | 5 |
| Tachigali vulgaris | 5, 8 |
| Talisia carinata | 5, 8 |
| Talisia furfuracea | 5 |
| Talisia guianensis | 5, 8 |
| Talisia longifolia | 5, 8 |
| Talisia megaphylla | 5, 8 |
| Talisia retusa | 5, 8 |
| Talisia squarrosa | 3, 5 |
| Tapirira guianensis | 1, 2, 5, 6, 8 |
| Tapirira obtusa | 5, 8 |
| Tapirira retusa | 5, 8 |
| Taralea oppositifolia | 5, 8 |
| Terminalia amazonia | 1, 2, 3, 5, 6, 8 |
| Terminalia aubletii | 5 |
| Terminalia congesta | 5, 8 |
| Terminalia dichotoma | 1, 3, 5, 6, 7, 8 |
| Terminalia fanshawei | 3, 5 |
| Terminalia grandis | 2, 5, 8 |
| Terminalia guyanensis | 1, 5, 6, 7, 8 |
| Terminalia nitidissima | 5 |
| Terminalia oblonga | 5 |
| Terminalia oxycarpa | 5, 8 |
| Terminalia parvifolia | 5, 8 |
| Terminalia tetraphylla | 1, 5, 6, 7, 8 |
| Theobroma obovatum | 5 |
| Thyrsodium guianense | 5, 8 |
| Thyrsodium puberulum | 5 |
| Thyrsodium spruceanum | 5, 8 |
| Tovomita obovata | 5 |
| Trattinnickia burserifolia | 1, 2, 5, 6, 7, 8 |
| Trattinnickia demerarae | 3, 5, 7, 8 |
| Trattinnickia glaziovii | 5, 8 |
| Trattinnickia rhoifolia | 1, 3, 5, 6, 7, 8 |
| Trichilia cipo | 5, 8 |
| Trichilia elegans | 5, 8 |
| Trichilia martiana | 5, 8 |
| Trichilia micrantha | 5, 8 |
| Trichilia pallida | 5, 8 |
| Trichilia pleeana | 5, 8 |
| Trichilia quadrijuga | 5, 8 |
| Trichilia rubra | 5, 8 |
| Trichilia schomburgkii | 5, 8 |
| Trichilia septentrionalis | 5, 8 |
| Trichilia surinamensis | 5, 7 |
| Triplaris weigeltiana | 5, 8 |
| Trymatococcus amazonicus | 5 |
| Vantanea guianensis | 5, 8 |
| Vantanea parviflora | 5, 8 |
| Vatairea erythrocarpa | 4, 5, 8 |
| Vatairea guianensis | 1, 3, 5, 6, 7, 8 |
| Vatairea paraensis | 5, 8 |
| Vatairea sericea | 5, 8 |
| Vataireopsis speciosa | 5, 7, 8 |
| Vataireopsis surinamensis | 1, 5, 6 |
| Virola caducifolia | 5, 8 |
| Virola calophylla | 5, 8 |
| Virola elongata | 5, 8 |
| Virola kwatae | 4, 5 |
| Virola michelii | 1, 3, 4, 5, 6, 7, 8 |
| Virola minutiflora | 5, 8 |
| Virola mollissima | 5, 8 |
| Virola multicostata | 5, 8 |
| Virola multinervia | 5, 8 |
| Virola pavonis | 5, 8 |
| Virola sebifera | 5, 7, 8 |
| Virola surinamensis | 1, 2, 3, 4, 5, 6, 7, 8 |
| Virola theiodora | 5, 8 |
| Vitex guianensis | 5 |
| Vitex stahelii | 3, 5 |
| Vitex triflora | 5 |
| Vochysia densiflora | 5, 7 |
| Vochysia divergens | 5, 8 |
| Vochysia guianensis | 1, 4, 5, 6, 7, 8 |
| Vochysia lanceolata | 5 |
| Vochysia neyratii | 4, 5 |
| Vochysia surinamensis | 3, 4, 5, 8 |
| Vochysia tetraphylla | 3, 5 |
| Vochysia tomentosa | 1, 4, 5, 6, 7 |
| Vochysia vismiifolia | 5, 8 |
| Vouacapoua americana | 1, 4, 5, 6, 7, 8 |
| Xylopia amazonica | 5, 8 |
| Xylopia aromatica | 5, 8 |
| Xylopia benthamii | 5, 8 |
| Xylopia emarginata | 5, 8 |
| Xylopia nitida | 5, 8 |
| Xylopia pulcherrima | 5 |
| Xylopia sericea | 5, 8 |
| Zanthoxylum acuminatum | 5, 8 |
| Zanthoxylum rhoifolium | 5, 8 |
| Zygia cataractae | 5, 8 |
| Zygia cauliflora | 5, 8 |
| Zygia latifolia | 5, 8 |
| Zygia racemosa | 4, 5, 7, 8 |

1 Bhiki, C.R., Maas, P.J., Koek-Noorman, J., van Andel, T. (2016). Timber Trees of Suriname - A field guide for the identification of timber trees based on field, vegetative, floristic and wood characteristics. Jansen-Jacobs, M.J. (editor). LM Publishers. ISBN 9789460223914.

2 Brazilian Forest Service (SFB)(2016). Spécies madeireiras de interesse comercial. Data de atualização: 23/06/2016. Retrieved from: <http://snif.florestal.gov.br/images/xls/recursos_florestais/especies_florestais_especies_madeireiras_interesse_comercial_2016.csv>

3 Gérard, J., Miller, R.B., ter Welle, B.J.H. (1996). Major Timber Trees of Guyana - Timber Characteristics and Utilization. The Tropenbos Foundation. Tropenbos series 15. ISSN 1383-68111.

4 Guitet S., Brunaux, O., Traissac, S. (2014). Sylviculture pour la production de bois d’oeuvre des forêts du Nord de la Guyane - Etat des connaissances et recommandations*.* Office National des Forets (ONF). Retrieved from: <http://www1.onf.fr/guyane/++oid++57df/@@display_media.html>

5 Piponiot, C., Rödig, E., Putz, F. E., Rutishauser, E., Sist, P., Ascarrunz, N., Blanc, L., Derroire, G., Descroix, L., Guedes, M. C., Coronado, E. H., Huth, A., Kanashiro, M., Licona, J. C., Mazzei, L., D’Oliveira, M. V. N., Peña-Claros, M., Rodney, K., Shenkin, A., … Hérault, B. (2019). Can timber provision from Amazonian production forests be sustainable? Environmental Research Letters, 14(6), 64014. https://doi.org/10.1088/1748-9326/ab195e

6 Suriname Forestry Service (SBB) (2016). Production, export and import of Timber and Timberproducts 2017. Retrieved from: <https://sbbsur.com/>

7 Suriname Forestry Service (SBB) (2017). Production, export and import of Timber and Timberproducts 2017. Retrieved from: <https://sbbsur.com/>

8 State of Para (2010). *Instrução Normativa IDEFLOR nº 2 de 08/07/2010*. Retrieved from: <https://www.legisweb.com.br/legislacao/?id=148018>

#### Table S1.2. List of commercially relevant NTFP taxa in the Guiana Shield region

| **NTFP taxon** | **Main use** | **References** |
| --- | --- | --- |
| *Ambelania acida* | Food | 9 |
| *Anacardium giganteum* | Food | 9 |
| *Aniba rosiodora* | Medicinal | 6, 8, |
| *Annona sericea* | Food, Medicinal | 9 |
| *Aspidosperma album* | Crafts | 6, 9 |
| *Aspidosperma araracanga* | Crafts | 6, 9 |
| *Aspidosperma carapanauba* | Crafts | 6, 9 |
| *Aspidosperma cuspa* | Crafts | 6, 9 |
| *Aspidosperma desmanthum* | Crafts | 6, 9 |
| *Aspidosperma discolor* | Crafts | 6, 9 |
| *Aspidosperma excelsum* | Crafts | 6, 9 |
| *Aspidosperma helstonei* | Crafts | 6, 9 |
| *Aspidosperma megalocarpon* | Crafts | 6, 9 |
| *Aspidosperma parvifolium* | Crafts | 6, 9 |
| *Aspidosperma sandwithianum* | Crafts | 6, 9 |
| *Aspidosperma schultesii* | Crafts | 6, 9 |
| *Aspidosperma spp. (84 morphospecies)* | Crafts | 6, 9 |
| *Astrocaryum sciophilum* | Food, Medicinal, Crafts | 9 |
| *Attalea maripa* | Food, Medicinal, Crafts | 6, 3, 8, 9 |
| *Bagassa guianensis* | Crafts | 9 |
| *Bellucia grossularioides* | Food, Medicinal | 9 |
| *Bixa orellana* | Paint, Rituals | 9 |
| *Brosimum guianense* | Crafts | 9 |
| *Brosimum parinarioides* | Crafts | 8 |
| *Brosimum potabile* | Crafts | 8 |
| *Brosimum rubescens* | Crafts | 6, 8, 9 |
| *Brosimum utile* | Crafts | 6 |
| *Byrsonima crassifolia* | Medicinal | 9 |
| *Campomanesia aromatica* | Bathing, Medicinal | 9 |
| *Carapa guianensis* | Medicinal | 6, 8, 5, 9 |
| *Carapa spp. (4 morphospecies)* | Medicinal | 9 |
| *Carapa surinamensis* | Medicinal | 9 |
| *Caryocar nuciferum* | Food | 6 |
| *Caryocar villosum* | Food, Crafts | 8 |
| *Caryodendron amazonicum* | Food | 8 |
| *Casearia arborea* | Medicinal, Rituals | 9 |
| *Cecropia obtusa* | Rituals | 9 |
| *Cecropia peltata* | Medicinal, Rituals | 9 |
| *Cecropia sciadophylla* | Rituals | 9 |
| *Cedrela odorata* | Crafts, Medicinal | 6, 9 |
| *Clathrotropis brachypetala* | Medicinal | 9 |
| *Clusia grandiflora* | Crafts | 6 |
| *Clusia hoffmannseggiana* | Crafts | 6 |
| *Copaifera epunctata* | Medicinal | 6, 1, 4, 7, 8, 9 |
| *Copaifera guyanensis* | Medicinal | 6, 1, 4, 7, 8, 9 |
| *Copaifera martii* | Medicinal | 6, 1, 4, 7, 8, 9 |
| *Copaifera pubiflora* | Medicinal | 6, 1, 4, 7, 8, 9 |
| *Copaifera spp. (4 morphospecies)* | Medicinal | 6, 1, 4, 7, 8, 9 |
| *Cordia tetrandra* | Food, Medicinal | 9 |
| *Couma guianensis* | Crafts | 6, 8, |
| *Couma macrocarpa* | Crafts | 6 |
| *Couratari stellata* | Crafts, Rituals | 9 |
| *Dicorynia guianensis* | Crafts | 9 |
| *Didymopanax morototoni* | Crafts, Medicinal | 9 |
| *Dipteryx odorata* | Food, Medicinal, Hair product | 6, 8, 9 |
| *Duguetia pycnastera* | Crafts | 5 |
| *Endopleura uchi* | Food, Medicinal | 8 |
| *Eperua falcata* | Crafts, Rituals | 9 |
| *Eugenia moschata* | Food | 8 |
| *Eugenia patrisii* | Food | 9 |
| *Euterpe oleracea* | Food, Crafts, Rituals | 6, 2, 8, 5, 9 |
| *Euterpe precatoria* | Food, Crafts, Rituals | 6, 1, 8, 9 |
| *Euterpe spp.* | Food, Crafts | 9 |
| *Ficus insipida* | Crafts | 6, 9 |
| *Ficus nymphaeifolia* | Rituals | 9 |
| *Garcinia macrophylla* | Food | 8, 9 |
| *Garcinia madruno* | Food | 8 |
| *Geissospermum sericeum* | Medicinal | 9 |
| *Genipa americana* | Food | 6 |
| *Goupia glabra* | Medicinal | 9 |
| *Guarea gomma* | Medicinal, Rituals | 9 |
| *Guatteria schomburgkiana* | Food, Bathing, Medicinal | 9 |
| *Gustavia augusta* | Medicinal | 9 |
| *Handroanthus impetiginosus* | Medicinal | 8 |
| *Handroanthus serratifolius* | Medicinal | 8 |
| *Hevea benthamiana* | Crafts | 6 |
| *Hevea guianensis* | Crafts | 6 |
| *Hevea pauciflora* | Crafts | 6 |
| *Hevea spp. (5 morphospecies)* | Crafts | 6, 8 |
| *Himatanthus sucuuba* | Medicinal | 8 |
| *Hirtella paniculata* | Bathing, Medicinal | 9 |
| *Hymenaea courbaril* | Food, Medicinal | 6, 8 |
| *Inga alba* | Crafts, Medicinal | 8, 9 |
| *Inga capitata* | Food | 8 |
| *Inga cayennensis* | Food | 8 |
| *Inga cinnamomea* | Food | 8 |
| *Inga edulis* | Food | 8 |
| *Inga heterophylla* | Food | 9 |
| *Inga ingoides* | Food | 8 |
| *Inga laurina* | Food | 8 |
| *Inga macrophylla* | Food | 8 |
| *Inga stipularis* | Food | 8 |
| *Inga virgultosa* | Food | 9 |
| *Jacaranda copaia* | Medicinal, Crafts | 9 |
| *Leonia cymosa* | Medicinal | 9 |
| *Licania membranacea* | Medicinal, Rituals | 9 |
| *Lueheopsis rosea* | Rituals | 9 |
| *Lueheopsis rugosa* | Medicinal, Rituals | 9 |
| *Manicaria saccifera* | Crafts | 6, 5 |
| *Manilkara bidentata* | Crafts | 6 |
| *Manilkara elata* | Crafts | 6, 8 |
| *Maprounea guianensis* | Medicinal | 9 |
| *Mauritia flexuosa* | Food, Crafts | 6, 3, 8, 5, 9 |
| *Miconia lepidota* | Bathing, Medicinal | 9 |
| *Miconia prasina* | Bathing, Medicinal | 9 |
| *Myrciaria dubia* | Food | 6, 8 |
| *Myrciaria floribunda* | Bathing, Medicinal | 9 |
| *Ocotea guianensis* | Hair product, Crafts, Medicinal | 9 |
| *Oenocarpus bacaba* | Food | 6, 3, 9 |
| *Oenocarpus bataua* | Food | 6, 8 |
| *Ormosia amazonica* | Crafts | 8, 9 |
| *Ormosia bolivarensis* | Crafts | 8, 9 |
| *Ormosia coarctata* | Crafts | 8, 9 |
| *Ormosia coccinea* | Crafts | 8, 9 |
| *Ormosia costulata* | Crafts | 8, 9 |
| *Ormosia coutinhoi* | Crafts | 8, 9 |
| *Ormosia discolor* | Crafts | 8, 9 |
| *Ormosia flava* | Crafts | 8, 9 |
| *Ormosia grossa* | Crafts | 8, 9 |
| *Ormosia lignivalvis* | Crafts | 8, 9 |
| *Ormosia macrophylla* | Crafts | 8, 9 |
| *Ormosia melanocarpa* | Crafts | 8, 9 |
| *Ormosia nobilis* | Crafts | 8, 9 |
| *Ormosia paraensis* | Crafts | 8, 9 |
| *Ormosia spp. (38 morphospecies)* | Crafts | 8, 9 |
| *Ormosia stipularis* | Crafts | 8, 9 |
| *Palicourea guianensis* | Medicinal, Rituals | 8, 9 |
| *Parahancornia fasciculata* | Food | 8 |
| *Parinari campestris* | Food, Crafts, Rituals | 9 |
| *Parkia pendula* | Medicinal, Rituals | 9 |
| *Parkia ulei* | Crafts | 9 |
| *Platonia insignis* | NA | 8 |
| *Poraqueiba guianensis* | Food | 8 |
| *Poraqueiba sericea* | Food | 6, 8 |
| *Pouteria caimito* | Food | 8 |
| *Pouteria glomerata* | Food | 8 |
| *Protium altissimum* | Crafts, Rituals | 9 |
| *Protium heptaphyllum* | Crafts, Rituals | 9 |
| *Protium stevensonii* | Crafts, Rituals | 9 |
| *Pseudopiptadenia suaveolens* | Medicinal, Rituals | 9 |
| *Ptychopetalum olacoides* | Medicinal | 8 |
| *Quararibea guianensis* | Crafts, Rituals | 9 |
| *Quiina guianensis* | Crafts | 5 |
| *Simaba orinocensis* | Rituals | 9 |
| *Siparuna guianensis* | Medicinal, Rituals | 9 |
| *Sloanea grandiflora* | Rituals | 9 |
| *Spondias mombin* | Food | 6, 8 |
| *Spondias spp.* | Food | 8 |
| *Symphonia globulifera* | Crafts, Medicinal, Rituals | 9 |
| *Tabebuia insignis* | Medicinal | 5 |
| *Tabernaemontana siphilitica* | Medicinal, Rituals | 9 |
| *Tabernaemontana undulata* | Medicinal, Rituals | 9 |
| *Tachigali melinonii* | Medicinal | 9 |
| *Terminalia amazonia* | Bathing, Medicinal | 9 |
| *Theobroma cacao* | Food, Rituals | 8 |
| *Virola michelii* | Medicinal | 8 |
| *Virola sebifera* | Medicinal | 6 |
| *Virola surinamensis* | Medicinal | 6 |
| *Vismia cayennensis* | Bathing, Medicinal | 9 |
| *Vismia guianensis* | Medicinal | 9 |
| *Vismia japurensis* | Medicinal | 9 |
| *Vismia latifolia* | Medicinal | 9 |
| *Vismia macrophylla* | Medicinal | 9 |
| *Vouarana guianensis* | Rituals | 9 |
| *Zygia latifolia* | Medicinal, Rituals | 9 |
| *Zygia racemosa* | Medicinal | 9 |

1 Baraloto, C., Alverga, P., Quispe, S. B., Barnes, G., Chura, N. B., Brasil, I., Medeiros, H., Murphy, S., Rockwell, C. A., Shenkin, A., Silveira, M., & Southworth, J. (2014). Trade-offs among forest value components in community forests of southwestern Amazonia. 19(4).

2 Lopes, E., Soares-filho, B., Souza, F., Rajão, R., Merry, F., & Ribeiro, S. C. (2018). Landscape and Urban Planning Mapping the socio-ecology of Non Timber Forest Products ( NTFP ) extraction in the Brazilian Amazon : The case of açaí ( Euterpe precatoria Mart ) in Acre. January. https://doi.org/10.1016/j.landurbplan.2018.08.025

3 Moscoso, V., Albernaz, A. L., & Salomão, R. D. P. (2013). Niche modelling for twelve plant species (six timber species and six palm trees) in the Amazon region, using collection and field survey data. Forest Ecology and Management, 310, 652–662. https://doi.org/10.1016/j.foreco.2013.08.064

4 Newton, P., Peres, C. A., Desmoulière, S. J. M., & Watkinson, A. R. (2012). Cross-scale variation in the density and spatial distribution of an Amazonian non-timber forest resource. Forest Ecology and Management, 276, 41–51. https://doi.org/10.1016/j.foreco.2012.03.020

5 van Andel, T. R. (2000). Useful plant species in the seven forest hectare plots. In Non-Timber Forest Products of the North-West District of Guyana (pp. 94–146). Tropenbos-Guyana Programme-Georgetown, Guyana.

6 van Andel, T. R., Mackinven, A., & Bánki, O. (2003). Commercial Non-Timber Forest Products of the Guiana Shield - An inventory of commercial NTFP extraction. IUCN-NL. https://portals.iucn.org/library/node/8319

7 Plowden, C. (2004). The ethnobotany of copaíba (Copaifera) oleoresin in the Amazon. Economic Botany, 58(4), 729–733. https://doi.org/10.1663/0013-0001(2004)058[0729:TEOCCO]2.0.CO;2

8 Shanley, P., Cymerys, M., Serra, M., & Medina, G. (2011). Fruit trees and useful plants in Amazonian life. English edition. Food and Agriculture Organization of the United Nations, the Center for International Forestry Research and People and Plants International.

9 Steur, G., Verburg, R. W., Wassen, M. J., Teunissen, P. A., & Verweij, P. A. (2021). Exploring relationships between abundance of non-timber forest product species and tropical forest plant diversity. Ecological Indicators, 121(December 2020), 107202. https://doi.org/10.1016/j.ecolind.2020.107202

#### Table S1.3. Summary of plot data

Summary of the 283 1-ha plots used in this paper. AGC = Aboveground carbon stock estimates sensu method Sullivan et al. (2017) (Mg ha^-1^), Timber = commercially relevant timber stock (m^3^ ha^-1^), NTFPs = commercially relevant NTFP species abundance (# stems ha^-1^), Spp = woody species richness (# species ha^-1^). For visualization, all numbers have been truncated to two decimal points.

| **Plot_code** | **Country** | **Site_description** | **Latitude** | **Longitude** | **AGC** | **Timber** | **NTFPs** | **Spp** | **Region** | **Forest_type** | **Subregion** | **Guiana_Shield** | **Amazonia** | **Census_year** | **Owner/contact** | **Reference** |
| --- | --- | --- | --- | --- | --- | --- | --- | --- | --- | --- | --- | --- | --- | --- | --- | --- |
| AL_01 | Suriname | Alberga 1, road to | 5.34 | -55.77 | 178.4 | 105.71 | 246 | 78 | GS | TF | NPS | Y | Y | 2006 | Hans ter Steege, Olaf Bánki | 1 |
| GOL_01 | Suriname | Goliath 1 | 5.22 | -55.65 | 257.5 | 55.70 | 53 | 45 | GS | PZ | NPS | Y | Y | 2006 | Hans ter Steege, Olaf Bánki | 1 |
| BIT_02 | Suriname | Bitagron 2 | 5.08 | -56.2 | 226.3 | 66.26 | 289 | 42 | GS | PZ | NPS | Y | Y | 2006 | Hans ter Steege, Olaf Bánki | 2 |
| BIT_03 | Suriname | Bitagron 3 | 5.08 | -56.2 | 275.6 | 146.40 | 234 | 37 | GS | PZ | NPS | Y | Y | 2006 | Hans ter Steege, Olaf Bánki | 2 |
| BSH_01 | Suriname | BSH 1 | 5.14 | -55.78 | 271.6 | 63.53 | 225 | 33 | GS | PZ | NPS | Y | Y | 2006 | Hans ter Steege, Olaf Bánki | 3 |
| BSH_02 | Suriname | BSH 2 | 5.15 | -55.78 | 218.2 | 100.29 | 235 | 36 | GS | PZ | NPS | Y | Y | 2006 | Hans ter Steege, Olaf Bánki | 3 |
| BSH_03 | Suriname | BSH 3 | 5.15 | -55.78 | 248.2 | 141.09 | 280 | 31 | GS | PZ | NPS | Y | Y | 2006 | Hans ter Steege, Olaf Bánki | 3 |
| AL_02 | Suriname | Alberga 2, road to | 5.34 | -55.76 | 134.4 | 58.76 | 156 | 85 | GS | TF | NPS | Y | Y | 2006 | Hans ter Steege, Olaf Bánki | 4 |
| BIT_01 | Suriname | Bitagron 1 | 5.07 | -56.21 | 211.5 | 155.42 | 118 | 114 | GS | TF | NPS | Y | Y | 2006 | Hans ter Steege, Olaf Bánki | 4 |
| MHFR_01 | Guyana | Mabura Hill Forest Reserve, MHFR 1 (FMH-03) | 5.18 | -58.7 | 350.6 | 113.16 | 12 | 64 | GS | PZ | NPS | Y | Y | 2006 | Hans ter Steege, Olaf Bánki | 4 |
| MHFR_02 | Guyana | Mabura Hill Forest Reserve, MHFR 2 | 5.16 | -58.71 | 303.6 | 114.24 | 8 | 53 | GS | PZ | NPS | Y | Y | 2006 | Hans ter Steege, Olaf Bánki | 4 |
| MHFR_03 | Guyana | Mabura Hill Forest Reserve, MHFR 3 | 5.18 | -58.7 | 210.5 | 64.99 | 186 | 68 | GS | PZ | NPS | Y | Y | 2006 | Hans ter Steege, Olaf Bánki | 4 |
| NIR_01 | Suriname | Nickeri rivier 1 | 4.98 | -57 | 196.5 | 0.00 | 28 | 23 | GS | PZ | NPS | Y | Y | 2006 | Hans ter Steege, Olaf Bánki | 4 |
| NIR_02 | Suriname | Nickeri rivier 2 | 4.98 | -57 | 192 | 56.93 | 128 | 42 | GS | PZ | NPS | Y | Y | 2006 | Hans ter Steege, Olaf Bánki | 4 |
| NIR_03 | Suriname | Nickeri rivier 3, vicinity of | 4.99 | -57 | 242.2 | 152.53 | 173 | 120 | GS | TF | NPS | Y | Y | 2006 | Hans ter Steege, Olaf Bánki | 4 |
| NIR04 | Suriname | Nickeri rivier 4, vicinity of | 5 | -57 | 190.8 | 84.80 | 162 | 123 | GS | TF | NPS | Y | Y | 2006 | Hans ter Steege, Olaf Bánki | 4 |
| PIB_05 | Guyana | Mabura Hill, Pibiri 05 | 5.02 | -58.62 | 211.2 | 90.23 | 61 | 116 | GS | TF | NPS | Y | Y | 2006 | Hans ter Steege, Olaf Bánki | 4 |
| PIB_06 | Guyana | Mabura Hill, Pibiri 06 | 5.01 | -58.62 | 198.9 | 192.60 | 59 | 95 | GS | TF | NPS | Y | Y | 2006 | Hans ter Steege, Olaf Bánki | 4 |
| PIB_12 | Guyana | Mabura Hill, Pibiri 12 LE | 5.03 | -58.6 | 229.1 | 188.00 | 70 | 130 | GS | TF | NPS | Y | Y | 2006 | Hans ter Steege, Olaf Bánki | 4 |
| SIMCR_01 | Suriname | Simonskreek 1 | 5.32 | -54.94 | 137.9 | 6.63 | 58 | 43 | GS | PZ | NPS | Y | Y | 2006 | Hans ter Steege, Olaf Bánki | 4 |
| SIMCR_02 | Suriname | Simonskreek 2 | 5.32 | -54.94 | 132.3 | 0.00 | 17 | 31 | GS | PZ | NPS | Y | Y | 2006 | Hans ter Steege, Olaf Bánki | 4 |
| NM_01P | Suriname | Nassau Mts 1 | 4.78 | -54.62 | 196.5 | 122.27 | 147 | 112 | GS | TF | SGS | Y | Y | 2003 | Hans ter Steege, Olaf Bánki | 5 |
| NM_03P | Suriname | Nassau Mts 3 | 4.82 | -54.6 | 231.6 | 184.32 | 77 | 131 | GS | TF | SGS | Y | Y | 2003 | Hans ter Steege, Olaf Bánki | 5 |
| NM_04L | Suriname | Nassau Mts 4 | 4.93 | -54.52 | 226.1 | 75.24 | 219 | 143 | GS | TF | NPS | Y | Y | 2003 | Hans ter Steege, Olaf Bánki | 5 |
| NM_05L | Suriname | Nassau Mts 5 | 4.93 | -54.52 | 216.7 | 49.88 | 179 | 141 | GS | TF | NPS | Y | Y | 2003 | Hans ter Steege, Olaf Bánki | 5 |
| NM_06P | Suriname | Nassau Mts 6 | 4.83 | -54.61 | 259.3 | 119.28 | 89 | 137 | GS | TF | SGS | Y | Y | 2003 | Hans ter Steege, Olaf Bánki | 5 |
| ELB_01 | Venezuela | El Buey 01 | 8.24 | -62.18 | 199.5 | 91.58 | 82 | 87 | GS | TF | NWGS | Y | Y | 1993 | Hernan Castellanos | 6 |
| ELB_02 | Venezuela | El Buey 02 | 8.25 | -62.18 | 152.2 | 58.43 | 75 | 94 | GS | TF | NWGS | Y | Y | 1993 | Hernan Castellanos | 6 |
| ELB_03 | Venezuela | El Buey 03 | 8.24 | -62.18 | 173.5 | 51.15 | 73 | 75 | GS | TF | NWGS | Y | Y | 1993 | Hernan Castellanos | 6 |
| ELB_04 | Venezuela | El Buey 04 | 8.25 | -62.18 | 194.2 | 40.39 | 82 | 80 | GS | TF | NWGS | Y | Y | 1993 | Hernan Castellanos | 6 |
| ELB_05 | Venezuela | El Buey 05 | 8.24 | -62.17 | 175.8 | 54.70 | 81 | 81 | GS | TF | NWGS | Y | Y | 1993 | Hernan Castellanos | 6 |
| ELB_06 | Venezuela | El Buey 06 | 8.25 | -62.17 | 193.3 | 43.26 | 134 | 83 | GS | TF | NWGS | Y | Y | 1993 | Hernan Castellanos | 6 |
| ELB_07 | Venezuela | El Buey 07 | 8.24 | -62.18 | 169.2 | 36.33 | 66 | 71 | GS | TF | NWGS | Y | Y | 1993 | Hernan Castellanos | 6 |
| ELB_08 | Venezuela | El Buey 08 | 8.23 | -62.17 | 182.2 | 65.24 | 127 | 79 | GS | TF | NWGS | Y | Y | 1993 | Hernan Castellanos | 6 |
| ELB_09 | Venezuela | El Buey 09 | 8.23 | -62.17 | 150.7 | 36.42 | 125 | 75 | GS | TF | NWGS | Y | Y | 1993 | Hernan Castellanos | 6 |
| ELB_10 | Venezuela | El Buey 10 | 8.23 | -62.17 | 182.7 | 83.14 | 133 | 91 | GS | TF | NWGS | Y | Y | 1993 | Hernan Castellanos | 6 |
| ELB_11 | Venezuela | El Buey 11 | 8.23 | -62.17 | 118.3 | 73.38 | 91 | 68 | GS | TF | NWGS | Y | Y | 1993 | Hernan Castellanos | 6 |
| ELB_12 | Venezuela | El Buey 12 | 8.23 | -62.17 | 161.5 | 37.49 | 86 | 80 | GS | TF | NWGS | Y | Y | 1993 | Hernan Castellanos | 6 |
| ELB_13 | Venezuela | El Buey 13 | 8.23 | -62.17 | 109.9 | 53.47 | 92 | 81 | GS | TF | NWGS | Y | Y | 1993 | Hernan Castellanos | 6 |
| ELB_14 | Venezuela | El Buey 14 | 8.23 | -62.16 | 129.4 | 149.88 | 96 | 60 | GS | TF | NWGS | Y | Y | 1993 | Hernan Castellanos | 6 |
| ELB_15 | Venezuela | El Buey 15 | 8.23 | -62.16 | 135.2 | 59.36 | 125 | 64 | GS | TF | NWGS | Y | Y | 1993 | Hernan Castellanos | 6 |
| ELB_16 | Venezuela | El Buey 16 | 8.23 | -62.18 | 115.2 | 27.45 | 106 | 68 | GS | TF | NWGS | Y | Y | 1993 | Hernan Castellanos | 6 |
| ELB_17 | Venezuela | El Buey 17 | 8.23 | -62.18 | 187.8 | 97.97 | 58 | 61 | GS | TF | NWGS | Y | Y | 1993 | Hernan Castellanos | 6 |
| SPM | French Guiana | Piste de St Elie | 5.27 | -53.05 | 191.7 | 48.33 | 52 | 105 | GS | TF | NPS | Y | Y | 2001 | Daniel Sabatier, Émile Fonty | 7 |
| IWO_22 | Guyana | Iwokrama 22 | 4.63 | -58.74 | 274.4 | 199.14 | 31 | 79 | GS | PZ | NPS | Y | Y | 2010 | Roel Brienen | 8 |
| MHC_01 | Guyana | Mahdia | 5.32 | -59.14 | 299.9 | 137.78 | 77 | 69 | GS | TF | NPS | Y | Y | 2016 | Michelle Kalamandeen, David Galbraith | 9 |
| MHC_02 | Guyana | Mahdia | 5.3 | -58.91 | 201.3 | 60.03 | 19 | 90 | GS | TF | NPS | Y | Y | 2017 | Michelle Kalamandeen, David Galbraith | 9 |
| PUC_01 | Guyana | Puruni Plot 1 Control | 6.21 | -59.72 | 221.6 | 116.92 | 17 | 63 | GS | TF | NWGS | Y | Y | 2016 | Michelle Kalamandeen, David Galbraith | 9 |
| CNG_01 | Venezuela | Cerro Neblina Gentry | 0.83 | -66.17 | 189.8 | 10.09 | 15 | 166 | GS | PZ | SWPS | Y | Y | 1984 | Oliver Phillips | 10 |
| VG_CSN_1 | Suriname | Central Suriname Nature Reserve | 4.74 | -56.19 | 153.4 | 53.43 | 119 | 115 | GS | TF | SGS | Y | Y | 2015 | Linares-Palomino, R. and Wortel, V. | 10 |
| VG_CSN_2 | Suriname | Central Suriname Nature Reserve | 4.7 | -56.19 | 208.5 | 194.77 | 143 | 98 | GS | TF | SGS | Y | Y | 2015 | Linares-Palomino, R. and Wortel, V. | 10 |
| VG_CSN_3 | Suriname | Central Suriname Nature Reserve | 4.81 | -56.12 | 130.9 | 118.53 | 150 | 114 | GS | TF | SGS | Y | Y | 2015 | Linares-Palomino, R. and Wortel, V. | 10 |
| RERE_P032 | Venezuela | Rio Erebato | 5.52 | -64.61 | 136.7 | 142.91 | 39 | 71 | GS | TF | SWPS | Y | Y | 2015 | Hernan Castellanos | 11 |
| RERE_P033 | Venezuela | Rio Erebato | 5.53 | -64.61 | 144.7 | 71.89 | 31 | 76 | GS | TF | SWPS | Y | Y | 2015 | Hernan Castellanos | 11 |
| PSE10B | French Guiana | Piste de St Elie | 5.3 | -53.06 | 270.1 | 179.15 | 64 | 215 | GS | TF | NPS | Y | Y | 1988 | Daniel Sabatier, Jean-François Molino | 12 |
| PSE1B | French Guiana | Piste de St Elie | 5.3 | -53.05 | 288.5 | 144.16 | 113 | 182 | GS | TF | NPS | Y | Y | 1988 | Daniel Sabatier, Jean-François Molino | 12 |
| PSE2B | French Guiana | Piste de St Elie | 5.3 | -53.06 | 267.7 | 167.49 | 86 | 180 | GS | TF | NPS | Y | Y | 1988 | Daniel Sabatier, Jean-François Molino | 12 |
| PSE2C | French Guiana | Piste de St Elie | 5.3 | -53.06 | 209.8 | 107.65 | 128 | 181 | GS | TF | NPS | Y | Y | 1988 | Daniel Sabatier, Jean-François Molino | 12 |
| PSE3B | French Guiana | Piste de St Elie | 5.3 | -53.06 | 252.8 | 138.07 | 137 | 171 | GS | TF | NPS | Y | Y | 1988 | Daniel Sabatier, Jean-François Molino | 12 |
| PSE3C | French Guiana | Piste de St Elie | 5.3 | -53.06 | 221 | 120.40 | 88 | 158 | GS | TF | NPS | Y | Y | 1988 | Daniel Sabatier, Jean-François Molino | 12 |
| PSE4B | French Guiana | Piste de St Elie | 5.3 | -53.06 | 331 | 222.04 | 95 | 145 | GS | TF | NPS | Y | Y | 1988 | Daniel Sabatier, Jean-François Molino | 12 |
| PSE5B | French Guiana | Piste de St Elie | 5.3 | -53.06 | 244.6 | 163.64 | 106 | 140 | GS | TF | NPS | Y | Y | 1988 | Daniel Sabatier, Jean-François Molino | 12 |
| PSE6B | French Guiana | Piste de St Elie | 5.3 | -53.06 | 245.2 | 137.30 | 122 | 183 | GS | TF | NPS | Y | Y | 1988 | Daniel Sabatier, Jean-François Molino | 12 |
| PSE6F | French Guiana | Piste de St Elie | 5.3 | -53.06 | 268.8 | 134.26 | 89 | 174 | GS | TF | NPS | Y | Y | 1991 | Daniel Sabatier, Jean-François Molino | 12 |
| PSE6G | French Guiana | Piste de St Elie | 5.3 | -53.06 | 296.9 | 195.07 | 105 | 187 | GS | TF | NPS | Y | Y | 1991 | Daniel Sabatier, Jean-François Molino | 12 |
| PSE6H | French Guiana | Piste de St Elie | 5.3 | -53.06 | 327.3 | 247.43 | 66 | 184 | GS | TF | NPS | Y | Y | 1991 | Daniel Sabatier, Jean-François Molino | 12 |
| PSE6I | French Guiana | Piste de St Elie | 5.3 | -53.06 | 243.9 | 112.66 | 74 | 169 | GS | TF | NPS | Y | Y | 1991 | Daniel Sabatier, Jean-François Molino | 12 |
| PSE6J | French Guiana | Piste de St Elie | 5.3 | -53.06 | 224.2 | 140.55 | 82 | 182 | GS | TF | NPS | Y | Y | 1991 | Daniel Sabatier, Jean-François Molino | 12 |
| PSE7B | French Guiana | Piste de St Elie | 5.3 | -53.06 | 288 | 127.70 | 72 | 191 | GS | TF | NPS | Y | Y | 1988 | Daniel Sabatier, Jean-François Molino | 12 |
| PSE8B | French Guiana | Piste de St Elie | 5.3 | -53.06 | 259.3 | 151.91 | 124 | 204 | GS | TF | NPS | Y | Y | 1988 | Daniel Sabatier, Jean-François Molino | 12 |
| PSE9B | French Guiana | Piste de St Elie | 5.3 | -53.06 | 262.3 | 184.11 | 129 | 192 | GS | TF | NPS | Y | Y | 1988 | Daniel Sabatier, Jean-François Molino | 12 |
| PSEIV | French Guiana | Piste de St Elie | 5.29 | -53.06 | 269 | 100.73 | 93 | 219 | GS | TF | NPS | Y | Y | 1986 | Daniel Sabatier, Jean-François Molino | 12 |
| PSEP | French Guiana | Piste de St Elie | 5.29 | -53.06 | 290.2 | 220.96 | 64 | 179 | GS | TF | NPS | Y | Y | 1991 | Daniel Sabatier, Jean-François Molino | 12 |
| PSER | French Guiana | Piste de St Elie | 5.29 | -53.05 | 265.5 | 197.64 | 91 | 163 | GS | TF | NPS | Y | Y | 1991 | Daniel Sabatier, Jean-François Molino | 12 |
| BW_02 | Suriname | hectareplot 2 in Brownsweg, community forest Djanka Kondre HKV 308 | 4.98 | -55.2 | 237.5 | 186.22 | 89 | 121 | GS | TF | SGS | Y | Y | 2006 | Sofie Ruysschaert | 13 |
| BW_03 | Suriname | hectareplot 3 in Brownsweg, Birudumatu HKV 302 | 5.03 | -55.22 | 172.6 | 9.80 | 223 | 111 | GS | TF | NPS | Y | Y | 2006 | Sofie Ruysschaert | 13 |
| BW_04 | Suriname | hectareplot 4 in Brownsweg, Aboikoni HKV 302 | 4.99 | -55.29 | 172.5 | 102.64 | 121 | 127 | GS | TF | NPS | Y | Y | 2006 | Sofie Ruysschaert | 13 |
| POW_01 | Suriname | hectareplot 1 in Powakka, ERFPACHT TERREIN 200 | 5.46 | -55.06 | 178.6 | 134.53 | 256 | 109 | GS | TF | NPS | Y | Y | 2015 | Sofie Ruysschaert | 13 |
| ARW1 | French Guiana | Monts d'Arawa | 2.81 | -53.36 | 163.5 | 131.99 | 92 | 95 | GS | TF | SGS | Y | Y | 2006 | Daniel Sabatier, Jean-François Molino | 14 |
| ARW2 | French Guiana | Monts d'Arawa | 2.82 | -53.37 | 183.3 | 179.97 | 136 | 105 | GS | TF | SGS | Y | Y | 2006 | Daniel Sabatier, Jean-François Molino | 14 |
| ARW3 | French Guiana | Monts d'Arawa | 2.81 | -53.37 | 247.3 | 292.61 | 178 | 98 | GS | TF | SGS | Y | Y | 2006 | Daniel Sabatier, Jean-François Molino | 14 |
| BOU1 | French Guiana | Monts Tumuc Humac, Borne 1 | 2.21 | -54.45 | 209.8 | 128.01 | 223 | 168 | GS | TF | SGS | Y | Y | 2006 | Daniel Sabatier, Jean-François Molino | 14 |
| BOU2 | French Guiana | Monts Tumuc Humac, Borne 1 | 2.21 | -54.44 | 210.4 | 101.26 | 195 | 154 | GS | TF | SGS | Y | Y | 2006 | Daniel Sabatier, Jean-François Molino | 14 |
| BOU3 | French Guiana | Monts Tumuc Humac, Borne 1 | 2.21 | -54.44 | 236.9 | 147.64 | 216 | 203 | GS | TF | SGS | Y | Y | 2006 | Daniel Sabatier, Jean-François Molino | 14 |
| GF1_9 | French Guiana | Paracou, GUYAFLUX | 5.28 | -52.92 | 242.3 | 119.55 | 115 | 163 | GS | TF | SGS | Y | Y | 2004 | Daniel Sabatier, Jean-François Molino | 14 |
| GF4_8 | French Guiana | Paracou, GUYAFLUX | 5.28 | -52.92 | 165.1 | 50.65 | 73 | 137 | GS | TF | SGS | Y | Y | 2004 | Daniel Sabatier, Jean-François Molino | 14 |
| INI1 | French Guiana | Rivière Inini | 3.56 | -53.83 | 271.3 | 159.43 | 108 | 178 | GS | TF | SGS | Y | Y | 1990 | Daniel Sabatier, Jean-François Molino | 14 |
| INI2 | French Guiana | Rivière Inini | 3.56 | -53.83 | 279.6 | 144.02 | 173 | 176 | GS | TF | SGS | Y | Y | 1990 | Daniel Sabatier, Jean-François Molino | 14 |
| MEM1 | French Guiana | Massif des Emerillons | 3.25 | -53.09 | 211.1 | 166.25 | 135 | 139 | GS | TF | SGS | Y | Y | 2007 | Daniel Sabatier, Jean-François Molino | 14 |
| MEM2 | French Guiana | Massif des Emerillons | 3.26 | -53.08 | 248.2 | 278.11 | 86 | 124 | GS | TF | SGS | Y | Y | 2007 | Daniel Sabatier, Jean-François Molino | 14 |
| MEM3 | French Guiana | Massif des Emerillons | 3.25 | -53.08 | 237.9 | 231.58 | 129 | 127 | GS | TF | SGS | Y | Y | 2007 | Daniel Sabatier, Jean-François Molino | 14 |
| MPB1 | French Guiana | Montagne Plomb | 5 | -52.93 | 255.5 | 210.97 | 74 | 158 | GS | TF | SGS | Y | Y | 2002 | Daniel Sabatier, Jean-François Molino | 14 |
| MPB2 | French Guiana | Montagne Plomb | 5.01 | -52.93 | 212.8 | 53.50 | 143 | 163 | GS | TF | SGS | Y | Y | 2002 | Daniel Sabatier, Jean-François Molino | 14 |
| MPB3 | French Guiana | Montagne Plomb | 5.03 | -52.94 | 197.5 | 26.33 | 52 | 151 | GS | TF | SGS | Y | Y | 2004 | Daniel Sabatier, Jean-François Molino | 14 |
| MPB4 | French Guiana | Montagne Plomb | 5.03 | -52.94 | 229.8 | 131.14 | 114 | 115 | GS | TF | SGS | Y | Y | 2004 | Daniel Sabatier, Jean-François Molino | 14 |
| MPB5 | French Guiana | Montagne Plomb | 5.03 | -52.94 | 262.4 | 159.22 | 76 | 131 | GS | TF | SGS | Y | Y | 2005 | Daniel Sabatier, Jean-François Molino | 14 |
| MPB6 | French Guiana | Montagne Plomb | 5 | -52.95 | 224.8 | 151.37 | 71 | 194 | GS | TF | SGS | Y | Y | 2005 | Daniel Sabatier, Jean-François Molino | 14 |
| MPB7 | French Guiana | Montagne Plomb | 5.01 | -52.95 | 176.1 | 83.38 | 86 | 148 | GS | TF | SGS | Y | Y | 2005 | Daniel Sabatier, Jean-François Molino | 14 |
| MPB8 | French Guiana | Montagne Plomb | 4.99 | -53 | 187.5 | 103.13 | 54 | 168 | GS | TF | SGS | Y | Y | 2005 | Daniel Sabatier, Jean-François Molino | 14 |
| NOU1 | French Guiana | Nouragues | 4.09 | -52.68 | 275.8 | 220.78 | 122 | 183 | GS | TF | SGS | Y | Y | 1987 | Daniel Sabatier, Jean-François Molino | 14 |
| NOU2 | French Guiana | Nouragues | 4.09 | -52.68 | 300.7 | 262.80 | 131 | 214 | GS | TF | SGS | Y | Y | 1987 | Daniel Sabatier, Jean-François Molino | 14 |
| NOU3 | French Guiana | Nouragues | 4.09 | -52.67 | 263.8 | 198.30 | 59 | 182 | GS | TF | SGS | Y | Y | 1989 | Daniel Sabatier, Jean-François Molino | 14 |
| NOU4 | French Guiana | Nouragues | 4.08 | -52.67 | 258.8 | 234.31 | 142 | 193 | GS | TF | SGS | Y | Y | 1989 | Daniel Sabatier, Jean-François Molino | 14 |
| NOU7 | French Guiana | Nouragues | 4.08 | -52.68 | 173.7 | 117.28 | 58 | 191 | GS | TF | SGS | Y | Y | 1994 | Daniel Sabatier, Jean-François Molino | 14 |
| NOU8 | French Guiana | Nouragues | 4.08 | -52.68 | 207.5 | 178.15 | 39 | 176 | GS | TF | SGS | Y | Y | 1994 | Daniel Sabatier, Jean-François Molino | 14 |
| PIA1 | French Guiana | Piton de l'Armontabo | 3.73 | -52.32 | 259.9 | 235.22 | 105 | 177 | GS | TF | SGS | Y | Y | 2006 | Daniel Sabatier, Jean-François Molino | 14 |
| SLD | French Guiana | Saül, La Douane | 3.62 | -53.18 | 260.2 | 311.15 | 157 | 176 | GS | TF | SGS | Y | Y | 2001 | Daniel Sabatier, Jean-François Molino | 14 |
| SRV1 | French Guiana | Savane roche Virginie | 4.19 | -52.14 | 249.2 | 176.10 | 86 | 169 | GS | TF | SGS | Y | Y | 2006 | Daniel Sabatier, Jean-François Molino | 14 |
| SRV2 | French Guiana | Savane roche Virginie | 4.18 | -52.14 | 226.4 | 145.94 | 112 | 176 | GS | TF | SGS | Y | Y | 2008 | Daniel Sabatier, Jean-François Molino | 14 |
| SRV3 | French Guiana | Savane roche Virginie | 4.19 | -52.15 | 283.4 | 147.40 | 162 | 215 | GS | TF | SGS | Y | Y | 2008 | Daniel Sabatier, Jean-François Molino | 14 |
| TORT1 | French Guiana | Montagne Tortue | 4.22 | -52.41 | 169.7 | 111.68 | 64 | 183 | GS | TF | SGS | Y | Y | 2002 | Daniel Sabatier, Jean-François Molino | 14 |
| TORT2 | French Guiana | Montagne Tortue | 4.22 | -52.41 | 196.1 | 132.82 | 83 | 202 | GS | TF | SGS | Y | Y | 2002 | Daniel Sabatier, Jean-François Molino | 14 |
| WAN1 | French Guiana | Rivière Wanapi | 2.51 | -53.82 | 193.4 | 221.82 | 152 | 105 | GS | TF | SGS | Y | Y | 2007 | Daniel Sabatier, Jean-François Molino | 14 |
| WAN2 | French Guiana | Rivière Wanapi | 2.51 | -53.83 | 162.4 | 140.26 | 117 | 123 | GS | TF | SGS | Y | Y | 2007 | Daniel Sabatier, Jean-François Molino | 14 |
| WAN3 | French Guiana | Rivière Wanapi | 2.52 | -53.83 | 206.7 | 250.69 | 152 | 94 | GS | TF | SGS | Y | Y | 2007 | Daniel Sabatier, Jean-François Molino | 14 |
| ICANA_01 | Brazil | TF PAMAALI, Sao Gabriel da Cachoeira / Rio Icana / comunidade de Jandu Cachoeira | 1.55 | -68.68 | 167.7 | 34.30 | 62 | 191 | GS | TF | SWPS | Y | Y | 2007 | Juliana Stropp, Hans ter Steege | 16 |
| ICANA_02 | Brazil | CP Jandu Cachoeira, Sao Gabriel da Cachoeira / Rio Icana / Igarape Pamaali / close to the Indigenous School | 1.48 | -68.71 | 112.3 | 2.10 | 10 | 116 | GS | PZ | SWPS | Y | Y | 2007 | Juliana Stropp, Hans ter Steege | 16 |
| SGC_01 | Brazil | TF 1 COMARA, Sao Gabriel da Cachoeira / airforce area | -0.08 | -66.89 | 203.4 | 41.48 | 22 | 125 | GS | TF | SWPS | Y | Y | 2008 | Juliana Stropp, Hans ter Steege | 16 |
| SGC_02 | Brazil | TF 2 COMARA, Sao Gabriel da Cachoeira / airforce area | -0.09 | -66.89 | 187.7 | 80.23 | 38 | 141 | GS | TF | SWPS | Y | Y | 2008 | Juliana Stropp, Hans ter Steege | 16 |
| SGC_03 | Brazil | TF Torre do LBA, Sao Gabriel da Cachoeira / Pico da Neblina National Park / LBA site | 0.21 | -66.77 | 180.6 | 53.01 | 26 | 154 | GS | TF | SWPS | Y | Y | 2008 | Juliana Stropp, Hans ter Steege | 16 |
| SGC_04 | Brazil | CP 1 COMARA, Sao Gabriel da Cachoeira / airforce area | -0.1 | -66.88 | 199.1 | 19.03 | 30 | 101 | GS | PZ | SWPS | Y | Y | 2008 | Juliana Stropp, Hans ter Steege | 16 |
| SGC_05 | Brazil | CP 2 COMARA, Sao Gabriel da Cachoeira / airforce area | -0.11 | -66.88 | 172.8 | 11.43 | 38 | 94 | GS | PZ | SWPS | Y | Y | 2008 | Juliana Stropp, Hans ter Steege | 16 |
| SGC_06 | Brazil | CP Itacoatiara-Mirim, Sao Gabriel da Cachoeira / comunidade de Itacoatiara-Mirim | -0.17 | -67.01 | 211 | 6.58 | 15 | 85 | GS | PZ | SWPS | Y | Y | 2008 | Juliana Stropp, Hans ter Steege | 16 |
| AGP-01 | Colombia | Amacayacu: Agua Pudre E | -3.72 | -70.31 | 121.2 | NA | NA | 265 | WAN | TF | NA | N | Y | 1992 | Sullivan et al. (2017) | 17 |
| AGP-02 | Colombia | Amacayacu: Agua Pudre U | -3.72 | -70.3 | 123.6 | NA | NA | 252 | WAN | TF | NA | N | Y | 1991 | Sullivan et al. (2017) | 17 |
| ALF-01 | Brazil | Alta Floresta plot 1 | -9.6 | -55.94 | 94.65 | NA | NA | 132 | BS | TF | NA | N | Y | 2002 | Sullivan et al. (2017) | 17 |
| ALF-02 | Brazil | Alta Floresta plot 2 | -9.58 | -55.92 | 109.8 | NA | NA | 110 | BS | TF | NA | N | Y | 2008 | Sullivan et al. (2017) | 17 |
| ALP-01 | Peru | Allpahuayo A | -3.95 | -73.43 | 129.6 | NA | NA | 264 | WAN | TF | NA | N | Y | 1990 | Sullivan et al. (2017) | 17 |
| ALP-30 | Peru | Allpahuayo C | -3.95 | -73.43 | 107.3 | NA | NA | 100 | WAN | TF | NA | N | Y | 2001 | Sullivan et al. (2017) | 17 |
| ALP-40 | Peru | Allpahuayo D | -3.94 | -73.44 | 107.5 | NA | NA | 45 | WAN | PZ | NA | N | Y | 2006 | Sullivan et al. (2017) | 17 |
| AMD-01 | Brazil | Fazenda Amanda (inventario 5) | -1.83 | -46.75 | 123.2 | NA | NA | 138 | EA | TF | NA | N | Y | 1997 | Sullivan et al. (2017) | 17 |
| AMD-02 | Brazil | Fazenda Amanda (inventario 6) | -1.83 | -46.75 | 184 | NA | NA | 121 | EA | TF | NA | N | Y | 1997 | Sullivan et al. (2017) | 17 |
| BBC-01 | Bolivia | Bosque Continuo BC-01 | -14.3 | -60.53 | 117.4 | NA | NA | 65 | WAS | TF | NA | N | Y | 1999 | Sullivan et al. (2017) | 17 |
| BBC-02 | Bolivia | Bosque Continuo BC-02 | -14.3 | -60.53 | 93.59 | NA | NA | 57 | WAS | TF | NA | N | Y | 1999 | Sullivan et al. (2017) | 17 |
| BDF-01 | Brazil | BDFFP, 2303 Dimona.5-6 | -2.34 | -60.1 | 175.4 | NA | NA | 258 | CA | TF | NA | N | Y | 1985 | Sullivan et al. (2017) | 17 |
| BDF-03 | Brazil | BDFFP, 1101 Gaviao | -2.42 | -59.85 | 149.4 | NA | NA | 279 | CA | TF | NA | N | Y | 1981 | Sullivan et al. (2017) | 17 |
| BDF-04 | Brazil | BDFFP, 1102 Gaviao | -2.43 | -59.85 | 159.1 | NA | NA | 272 | CA | TF | NA | N | Y | 1981 | Sullivan et al. (2017) | 17 |
| BDF-05 | Brazil | BDFFP, 1103 Gaviao | -2.43 | -59.85 | 124.5 | NA | NA | 260 | CA | TF | NA | N | Y | 1981 | Sullivan et al. (2017) | 17 |
| BDF-06 | Brazil | BDFFP, 1201 Gaviao | -2.41 | -59.86 | 101.3 | NA | NA | 233 | CA | TF | NA | N | Y | 1981 | Sullivan et al. (2017) | 17 |
| BDF-07 | Brazil | BDFFP, 1105 Gaviao | -2.4 | -59.9 | 156.7 | NA | NA | 261 | CA | TF | NA | N | Y | 1981 | Sullivan et al. (2017) | 17 |
| BDF-08 | Brazil | BDFFP, 1109 Gaviao | -2.4 | -59.9 | 149.4 | NA | NA | 251 | CA | TF | NA | N | Y | 1981 | Sullivan et al. (2017) | 17 |
| BDF-09 | Brazil | BDFFP, 1113 Florestal | -2.4 | -59.85 | 170.8 | NA | NA | 271 | CA | TF | NA | N | Y | 1987 | Sullivan et al. (2017) | 17 |
| BDF-10 | Brazil | BDFFP, 1301 Florestal 1= plot 1301.1 and 1301.3 | -2.39 | -59.86 | 146.1 | NA | NA | 311 | CA | TF | NA | N | Y | 1983 | Sullivan et al. (2017) | 17 |
| BDF-11 | Brazil | BDFFP, 1301 Florestal 2= plots 1301.4,5,6 | -2.38 | -59.85 | 170.4 | NA | NA | 277 | CA | TF | NA | N | Y | 1983 | Sullivan et al. (2017) | 17 |
| BDF-12 | Brazil | BDFFP, 1301 Florestal 3=plots 1301.7,8 | -2.39 | -59.85 | 176.1 | NA | NA | 285 | CA | TF | NA | N | Y | 1983 | Sullivan et al. (2017) | 17 |
| BDF-13 | Brazil | BDFFP, 3402 Cabo Frio | -2.4 | -59.91 | 167.2 | NA | NA | 254 | CA | TF | NA | N | Y | 1985 | Sullivan et al. (2017) | 17 |
| BDF-14 | Brazil | BDFFP, 3304 Porto Alegre | -2.36 | -59.97 | 175 | NA | NA | 272 | CA | TF | NA | N | Y | 1984 | Sullivan et al. (2017) | 17 |
| BEE-01 | Bolivia | BEEM plot 1 | -16.53 | -64.58 | 107.2 | NA | NA | 90 | WAS | TF | NA | N | Y | 2002 | Sullivan et al. (2017) | 17 |
| BEE-05 | Bolivia | BEEM plot 5 | -16.53 | -64.58 | 96.87 | NA | NA | 81 | WAS | TF | NA | N | Y | 2002 | Sullivan et al. (2017) | 17 |
| BNT-01 | Brazil | Bionte 1 | -2.64 | -60.16 | 150.7 | NA | NA | 159 | CA | TF | NA | N | Y | 1986 | Sullivan et al. (2017) | 17 |
| BNT-02 | Brazil | Bionte 02 | -2.64 | -60.15 | 149.4 | NA | NA | 182 | CA | TF | NA | N | Y | 1986 | Sullivan et al. (2017) | 17 |
| BNT-04 | Brazil | Bionte 4 | -2.63 | -60.15 | 138.8 | NA | NA | 161 | CA | TF | NA | N | Y | 1986 | Sullivan et al. (2017) | 17 |
| CAX-08 | Brazil | Caxiuana Terra Preta | -1.85 | -51.47 | 110.9 | NA | NA | 85 | EA | TF | NA | N | Y | 2003 | Sullivan et al. (2017) | 17 |
| CDM-01 | Peru | Cabeza de Mono | -10.33 | -75.3 | 148.1 | NA | NA | 147 | WAS | TF | NA | N | Y | 1983 | Sullivan et al. (2017) | 17 |
| CHO-02 | Bolivia | Chore Bosque Lianas 2 | -14.34 | -61.16 | 35.79 | NA | NA | 122 | WAS | TF | NA | N | Y | 1996 | Sullivan et al. (2017) | 17 |
| CPP-01 | Brazil | Fazenda Santo Amaro 1 | -1.84 | -47.1 | 153.9 | NA | NA | 128 | EA | TF | NA | N | Y | 1997 | Sullivan et al. (2017) | 17 |
| CPP-02 | Brazil | Fazenda Santo Amaro 2 | -1.84 | -47.1 | 167.7 | NA | NA | 124 | EA | TF | NA | N | Y | 2002 | Sullivan et al. (2017) | 17 |
| CRP-01 | Bolivia | Cerro Pelao 1 | -14.54 | -61.5 | 77.72 | NA | NA | 60 | WAS | TF | NA | N | Y | 1994 | Sullivan et al. (2017) | 17 |
| CRP-02 | Bolivia | Cerro Pelao 2 | -14.54 | -61.5 | 89.04 | NA | NA | 66 | WAS | TF | NA | N | Y | 1994 | Sullivan et al. (2017) | 17 |
| CUZ-01 | Peru | Cuzco Amazonico, CUZAM1E | -12.54 | -69.06 | 114.3 | NA | NA | 144 | WAS | TF | NA | N | Y | 1989 | Sullivan et al. (2017) | 17 |
| CUZ-02 | Peru | Cuzco Amazonico, CUZAM1U | -12.54 | -69.06 | 104.6 | NA | NA | 126 | WAS | TF | NA | N | Y | 1989 | Sullivan et al. (2017) | 17 |
| CUZ-03 | Peru | Cuzco Amazonico, CUZAM2E | -12.53 | -69.05 | 96.59 | NA | NA | 125 | WAS | TF | NA | N | Y | 1989 | Sullivan et al. (2017) | 17 |
| CUZ-04 | Peru | Cuzco Amazonico, CUZAM2U | -12.54 | -69.05 | 119.8 | NA | NA | 158 | WAS | TF | NA | N | Y | 1989 | Sullivan et al. (2017) | 17 |
| DOI-01 | Brazil | RESEX Chico Mendes: Seringal Dois Irmãos 1 | -10.57 | -68.32 | 105.7 | NA | NA | 158 | WAS | TF | NA | N | Y | 1991 | Sullivan et al. (2017) | 17 |
| FLO-01 | Brazil | Fazenda Floresta, Ribeirão Cascalheira, MT - Plot 1 | -12.81 | -51.85 | 78.26 | NA | NA | 77 | BS | TF | NA | N | Y | 2008 | Sullivan et al. (2017) | 17 |
| GMT-01 | Brazil | Granja Marathon | -1.11 | -47.8 | 153.8 | NA | NA | 147 | EA | TF | NA | N | Y | 1997 | Sullivan et al. (2017) | 17 |
| HCC-11 | Bolivia | Huanchaca Uno I, bosque galeria | -13.91 | -60.82 | 160.8 | NA | NA | 141 | WAS | TF | NA | N | Y | 1996 | Sullivan et al. (2017) | 17 |
| HCC-12 | Bolivia | Huanchaca Uno 2, bosque galeria | -13.91 | -60.82 | 146.5 | NA | NA | 112 | WAS | TF | NA | N | Y | 1996 | Sullivan et al. (2017) | 17 |
| HCC-21 | Bolivia | Huanchaca Dos, plot1 | -14.53 | -60.74 | 101.2 | NA | NA | 88 | WAS | TF | NA | N | Y | 1996 | Sullivan et al. (2017) | 17 |
| HCC-22 | Bolivia | Huanchaca Dos, plot 2 | -14.53 | -60.73 | 114.8 | NA | NA | 108 | WAS | TF | NA | N | Y | 1996 | Sullivan et al. (2017) | 17 |
| HCC-23 | Bolivia | Isla Huanchaca, plot 1 | -14.56 | -60.75 | 100.5 | NA | NA | 122 | WAS | TF | NA | N | Y | 1999 | Sullivan et al. (2017) | 17 |
| IMA-03 | Venezuela | NA | 7.62 | -61.25 | 193.8 | NA | NA | 69 | GS | TF | NA | N | Y | 2014 | Sullivan et al. (2017) | 17 |
| IMA-04 | Venezuela | NA | 7.62 | -61.25 | 165.2 | NA | NA | 69 | GS | TF | NA | N | Y | 2014 | Sullivan et al. (2017) | 17 |
| IWO-03 | Guyana | Iwokrama 03, mixed forest | 4.53 | -58.78 | 254.4 | NA | NA | 74 | GS | TF | NA | N | Y | 2010 | Sullivan et al. (2017) | 17 |
| IWO-09 | Guyana | Iwokrama 9, white sand | 4.61 | -58.73 | 182.3 | NA | NA | 55 | GS | PZ | NA | N | Y | 2010 | Sullivan et al. (2017) | 17 |
| IWO-11 | Guyana | Iwokrama 11, Brown sand | 4.62 | -58.72 | 239.1 | NA | NA | 62 | GS | TF | NA | N | Y | 2010 | Sullivan et al. (2017) | 17 |
| IWO-12 | Guyana | Iwokrama 12, Turtle Mountain | 4.73 | -58.72 | 159.3 | NA | NA | 81 | GS | TF | NA | N | Y | 2010 | Sullivan et al. (2017) | 17 |
| IWO-21 | Guyana | Iwokrama 21, white sand | 4.63 | -58.74 | 181.7 | NA | NA | 40 | GS | PZ | NA | N | Y | 2002 | Sullivan et al. (2017) | 17 |
| JAS-02 | Ecuador | Jatun Sacha 2 | -1.07 | -77.62 | 114.9 | NA | NA | 239 | WAN | TF | NA | N | Y | 1987 | Sullivan et al. (2017) | 17 |
| JAS-03 | Ecuador | Jatun Sacha 3 | -1.08 | -77.61 | 109.2 | NA | NA | 218 | WAN | TF | NA | N | Y | 1988 | Sullivan et al. (2017) | 17 |
| JAS-04 | Ecuador | Jatun Sacha 4 Full plot | -1.07 | -77.61 | 151.3 | NA | NA | 245 | WAN | TF | NA | N | Y | 2007 | Sullivan et al. (2017) | 17 |
| JAS-05 | Ecuador | Jatun Sacha 5 Full plot (until 2007) | -1.06 | -77.62 | 121.7 | NA | NA | 180 | WAN | TF | NA | N | Y | 1989 | Sullivan et al. (2017) | 17 |
| JEN-11 | Peru | Jenaro Herrera A Terraza Alta | -4.88 | -73.63 | 133 | NA | NA | 262 | WAN | TF | NA | N | Y | 2005 | Sullivan et al. (2017) | 17 |
| JEN-12 | Peru | Jenaro Herrera B Varillal | -4.9 | -73.63 | 115 | NA | NA | 122 | WAN | PZ | NA | N | Y | 2005 | Sullivan et al. (2017) | 17 |
| JRI-01 | Brazil | Jari 1 | -0.89 | -52.19 | 199.3 | NA | NA | 150 | EA | TF | NA | N | Y | 1985 | Sullivan et al. (2017) | 17 |
| LAS-02 | Peru | Jacaratia Los Amigos | -12.57 | -70.09 | 117.4 | NA | NA | 189 | WAS | TF | NA | N | Y | 2008 | Sullivan et al. (2017) | 17 |
| LFB-01 | Bolivia | Los Fierros Bosque I | -14.58 | -60.83 | 103.6 | NA | NA | 105 | WAS | TF | NA | N | Y | 1993 | Sullivan et al. (2017) | 17 |
| LFB-02 | Bolivia | Los Fierros Bosque II | -14.58 | -60.83 | 125.1 | NA | NA | 83 | WAS | TF | NA | N | Y | 1993 | Sullivan et al. (2017) | 17 |
| LGB-01 | Bolivia | Las Gamas, bosque galeria | -14.8 | -60.39 | 138.7 | NA | NA | 89 | WAS | TF | NA | N | Y | 1993 | Sullivan et al. (2017) | 17 |
| LOR-01 | Colombia | Amacayacu: Lorena E | -3.06 | -69.99 | 142.8 | NA | NA | 232 | WAN | TF | NA | N | Y | 1992 | Sullivan et al. (2017) | 17 |
| LOR-02 (incl. LOR-03) | Colombia | Amacayacu: Lorena U subplot 1-25 | -3.06 | -69.99 | 135.1 | NA | NA | 226 | WAN | TF | NA | N | Y | 1992 | Sullivan et al. (2017) | 17 |
| MBT-01 | Bolivia | Mabet plot 01 | -10.07 | -65.89 | 99.82 | NA | NA | 143 | WAS | TF | NA | N | Y | 2010 | Sullivan et al. (2017) | 17 |
| MBT-02 | Bolivia | Mabet plot 02 | -10.05 | -65.89 | 105.4 | NA | NA | 140 | WAS | TF | NA | N | Y | 2010 | Sullivan et al. (2017) | 17 |
| MBT-04 | Bolivia | Mabet Plot 4 | -10.31 | -65.55 | 105.7 | NA | NA | 163 | WAS | TF | NA | N | Y | 2010 | Sullivan et al. (2017) | 17 |
| MBT-05 | Bolivia | Mabet plot 5 | -10.03 | -65.63 | 136.7 | NA | NA | 209 | WAS | TF | NA | N | Y | 2010 | Sullivan et al. (2017) | 17 |
| MBT-06 | Bolivia | Mabet plot 6 | -10.04 | -65.64 | 111.1 | NA | NA | 199 | WAS | TF | NA | N | Y | 2010 | Sullivan et al. (2017) | 17 |
| MBT-07 | Bolivia | Mabet plot 7 | -9.91 | -65.74 | 116.4 | NA | NA | 152 | WAS | TF | NA | N | Y | 2010 | Sullivan et al. (2017) | 17 |
| MBT-08 | Bolivia | Mabet plot 08 | -9.94 | -65.75 | 90.97 | NA | NA | 162 | WAS | TF | NA | N | Y | 2010 | Sullivan et al. (2017) | 17 |
| NCR-01 | Bolivia | Noel Monte Cristo, plot 1 | -14.64 | -61.16 | 169.5 | NA | NA | 64 | WAS | TF | NA | N | Y | 1995 | Sullivan et al. (2017) | 17 |
| NOU-11 | French Guiana | Nouragues Petit Plateau 20H | 4.08 | -52.68 | 227 | NA | NA | 205 | GS | TF | NA | N | Y | 1992 | Sullivan et al. (2017) | 17 |
| NOU-17 | French Guiana | Nouragues Petit Plateau 20F | 4.08 | -52.68 | 202.6 | NA | NA | 202 | GS | TF | NA | N | Y | 1992 | Sullivan et al. (2017) | 17 |
| NOU-18 | French Guiana | Nouragues Petit Plateau 21F | 4.08 | -52.68 | 210.8 | NA | NA | 226 | GS | TF | NA | N | Y | 1992 | Sullivan et al. (2017) | 17 |
| PAK-01 | Peru | Pakitza, Manu River, dissected alluvial, plot1 (TEAM VGCOU-3) | -11.94 | -71.28 | 105.3 | NA | NA | 139 | WAS | TF | NA | N | Y | 1991 | Sullivan et al. (2017) | 17 |
| PAY-01 | Ecuador | Payamino | -0.45 | -77.03 | 135.7 | NA | NA | 244 | WAN | TF | NA | N | Y | 1987 | Sullivan et al. (2017) | 17 |
| PNY-03 | Peru | Paujil-Ozuz Bosque Humedo Tropical | -10.31 | -75.29 | 104 | NA | NA | 180 | WAS | TF | NA | N | Y | 2004 | Sullivan et al. (2017) | 17 |
| PNY-04 | Peru | Paujil Bosque Humedo Tropical | -10.34 | -75.25 | 105.7 | NA | NA | 189 | WAS | TF | NA | N | Y | 2007 | Sullivan et al. (2017) | 17 |
| PNY-05 | Peru | Paujil-Venado Bosque Humedo Tropical | -10.35 | -75.25 | 128.2 | NA | NA | 156 | WAS | TF | NA | N | Y | 2008 | Sullivan et al. (2017) | 17 |
| PNY-06 | Peru | Paujil-Venado Bosque Humedo Tropical | -10.36 | -75.25 | 110.7 | NA | NA | 132 | WAS | TF | NA | N | Y | 2008 | Sullivan et al. (2017) | 17 |
| PNY-07 | Peru | Paujil-Venado Bosque Humedo Tropical | -10.35 | -75.26 | 84.41 | NA | NA | 201 | WAS | TF | NA | N | Y | 2008 | Sullivan et al. (2017) | 17 |
| PPB-01 | Brazil | Peixe-Boi Parcela 01 | -1.18 | -47.32 | 127.8 | NA | NA | 131 | EA | TF | NA | N | Y | 1991 | Sullivan et al. (2017) | 17 |
| PPB-02 | Brazil | Peixe-Boi Parcela 02 | -1.18 | -47.32 | 111.4 | NA | NA | 169 | EA | TF | NA | N | Y | 1991 | Sullivan et al. (2017) | 17 |
| PPB-03 | Brazil | Peixe-Boi Parcela 03 | -1.18 | -47.32 | 120.2 | NA | NA | 130 | EA | TF | NA | N | Y | 1991 | Sullivan et al. (2017) | 17 |
| PTA-01 | Venezuela | Venezia 2.1 | 5.11 | -67.74 | 122.2 | NA | NA | 100 | GS | TF | NA | N | Y | 2011 | Sullivan et al. (2017) | 17 |
| PTA-03 | Venezuela | Venezia 1.1 | 5.11 | -67.74 | 104 | NA | NA | 99 | GS | TF | NA | N | Y | 2011 | Sullivan et al. (2017) | 17 |
| PTB-01 | Brazil | Porto Trombetas | -1.17 | -56.41 | 212.6 | NA | NA | 191 | CA | TF | NA | N | Y | 1997 | Sullivan et al. (2017) | 17 |
| PTB-02 | Brazil | PortoTrombetas | -1.48 | -56.39 | 105.8 | NA | NA | 185 | CA | TF | NA | N | Y | 1997 | Sullivan et al. (2017) | 17 |
| RBR-01 | Brazil | Rondonia_BR-364 | -11 | -61.95 | 139.6 | NA | NA | 175 | BS | TF | NA | N | Y | 1986 | Sullivan et al. (2017) | 17 |
| RCS-01 | Peru | Reserva Comunal El Sira - Bosque Pre-Montano | -9.47 | -74.77 | 127 | NA | NA | 158 | WAS | TF | NA | N | Y | 2010 | Sullivan et al. (2017) | 17 |
| RCS-05 | Peru | Reserva Comunal El Sira - Llano Amazonico | -9.62 | -74.93 | 102.4 | NA | NA | 195 | WAS | TF | NA | N | Y | 2011 | Sullivan et al. (2017) | 17 |
| RET-05 | Bolivia | Reserva El Tigre 05 | -10.97 | -65.72 | 116.2 | NA | NA | 110 | WAS | TF | NA | N | Y | 1995 | Sullivan et al. (2017) | 17 |
| RET-06 | Bolivia | Reserva El Tigre 06 | -10.97 | -65.72 | 129.4 | NA | NA | 110 | WAS | TF | NA | N | Y | 1995 | Sullivan et al. (2017) | 17 |
| RET-08 | Bolivia | Reserva El Tigre 08 | -10.97 | -65.72 | 123.3 | NA | NA | 96 | WAS | TF | NA | N | Y | 1995 | Sullivan et al. (2017) | 17 |
| RET-09 | Bolivia | Reserva El Tigre 09 | -10.97 | -65.72 | 121.6 | NA | NA | 100 | WAS | TF | NA | N | Y | 1995 | Sullivan et al. (2017) | 17 |
| RPA-01 | Peru | Rio Pariamanu | -12.39 | -69.36 | 138 | NA | NA | 169 | WAS | TF | NA | N | Y | 1998 | Sullivan et al. (2017) | 17 |
| RPI-01 | Peru | Rio Piedras | -12.36 | -69.23 | 129.4 | NA | NA | 139 | WAS | TF | NA | N | Y | 1995 | Sullivan et al. (2017) | 17 |
| RTH-01 | Peru | Rio Tahuamanu | -11.37 | -69.66 | 141.9 | NA | NA | 156 | WAS | TF | NA | N | Y | 1996 | Sullivan et al. (2017) | 17 |
| SAA-01 | Brazil | Floresta Santana do Araguaia-Pará | -9.79 | -50.43 | 82.84 | NA | NA | 95 | BS | TF | NA | N | Y | 2011 | Sullivan et al. (2017) | 17 |
| SCR-04 | Venezuela | San Carlos de Rio Negro, MAB site, Tall Caatinga, plot A | 1.93 | -67.04 | 171.1 | NA | NA | 38 | GS | PZ | NA | N | Y | 2004 | Sullivan et al. (2017) | 17 |
| SCR-05 | Venezuela | San Carlos de Rio Negro, MAB site, Yevaro, plot B | 1.93 | -67.04 | 185.5 | NA | NA | 118 | GS | TF | NA | N | Y | 2004 | Sullivan et al. (2017) | 17 |
| SHI-01 | Ecuador | Shiripuno | -1.02 | -76.98 | 90.96 | NA | NA | 192 | WAN | TF | NA | N | Y | 1994 | Sullivan et al. (2017) | 17 |
| SNP-01 | Brazil | Serra Norte PA | -6.04 | -50.15 | 139.9 | NA | NA | 105 | BS | TF | NA | N | Y | 1987 | Sullivan et al. (2017) | 17 |
| SUC-01 | Peru | Sucusari A | -3.25 | -72.91 | 129.4 | NA | NA | 308 | WAN | TF | NA | N | Y | 1992 | Sullivan et al. (2017) | 17 |
| SUC-02 | Peru | Sucusari B | -3.25 | -72.9 | 133.4 | NA | NA | 278 | WAN | TF | NA | N | Y | 1992 | Sullivan et al. (2017) | 17 |
| SUC-04 | Peru | Sucusari D | -3.25 | -72.89 | 136.9 | NA | NA | 252 | WAN | TF | NA | N | Y | 2001 | Sullivan et al. (2017) | 17 |
| SUC-05 | Peru | Sucusari E | -3.26 | -72.89 | 129.5 | NA | NA | 186 | WAN | TF | NA | N | Y | 2001 | Sullivan et al. (2017) | 17 |
| TAM-01 | Peru | Tambopata plot zero | -12.84 | -69.29 | 105.1 | NA | NA | 165 | WAS | TF | NA | N | Y | 1983 | Sullivan et al. (2017) | 17 |
| TAM-02 | Peru | Tambopata plot one | -12.83 | -69.29 | 112.9 | NA | NA | 167 | WAS | TF | NA | N | Y | 1979 | Sullivan et al. (2017) | 17 |
| TAM-05 | Peru | Tambopata plot three | -12.83 | -69.27 | 109.4 | NA | NA | 166 | WAS | TF | NA | N | Y | 1983 | Sullivan et al. (2017) | 17 |
| TAM-06 | Peru | Tambopata plot four | -12.84 | -69.3 | 104.9 | NA | NA | 172 | WAS | TF | NA | N | Y | 1983 | Sullivan et al. (2017) | 17 |
| TAM-07 | Peru | Tambopata plot six | -12.83 | -69.26 | 120.4 | NA | NA | 155 | WAS | TF | NA | N | Y | 1983 | Sullivan et al. (2017) | 17 |
| TAM-08 | Peru | Tambopata plot seven | -12.83 | -69.27 | 103.4 | NA | NA | 136 | WAS | TF | NA | N | Y | 2001 | Sullivan et al. (2017) | 17 |
| TAN-02 | Brazil | Fazenda Tanguro, plot 2 | -13.09 | -52.38 | 73.32 | NA | NA | 53 | BS | TF | NA | N | Y | 2009 | Sullivan et al. (2017) | 17 |
| TAN-03 | Brazil | Fazenda Tanguro plot 3 | -12.82 | -52.36 | 84.11 | NA | NA | 50 | BS | TF | NA | N | Y | 2009 | Sullivan et al. (2017) | 17 |
| TAN-04 | Brazil | Fazenda Tanguro, plot 4 (TROBIT) | -12.92 | -52.37 | 75.38 | NA | NA | 54 | BS | TF | NA | N | Y | 2008 | Sullivan et al. (2017) | 17 |
| TEC-01 | Brazil | TEAM Caxiuana plot 1 VG-CAX-1 | -1.71 | -51.46 | 190.1 | NA | NA | 159 | EA | TF | NA | N | Y | 2002 | Sullivan et al. (2017) | 17 |
| TEC-02 | Brazil | TEAM Caxiuana plot 2 VG-CAX-2 | -1.74 | -51.49 | 215.2 | NA | NA | 197 | EA | TF | NA | N | Y | 2003 | Sullivan et al. (2017) | 17 |
| TEC-04 | Brazil | TEAM Caxiuana plot 4 VG-CAX-4 | -1.75 | -51.52 | 196.6 | NA | NA | 158 | EA | TF | NA | N | Y | 2003 | Sullivan et al. (2017) | 17 |
| TEC-05 | Brazil | TEAM Caxiuana plot 5 VG-CAX-5 | -1.78 | -51.59 | 212.7 | NA | NA | 180 | EA | TF | NA | N | Y | 2003 | Sullivan et al. (2017) | 17 |
| TEC-06 | Brazil | TEAM Caxiuana plot 6 VG-CAX-6 | -1.73 | -51.43 | 163.9 | NA | NA | 160 | EA | TF | NA | N | Y | 2003 | Sullivan et al. (2017) | 17 |
| TEM-03 | Brazil | TEAM Manaus plot 3: Cabo Frio VG-MAS-1 | -2.41 | -59.9 | 131.4 | NA | NA | 280 | CA | TF | NA | N | Y | 2003 | Sullivan et al. (2017) | 17 |
| TEM-04 | Brazil | TEAM Manaus plot 4 VG-MAS-4 | -2.43 | -59.79 | 116.6 | NA | NA | 265 | CA | TF | NA | N | Y | 2003 | Sullivan et al. (2017) | 17 |
| TEM-05 | Brazil | TEAM Manaus plot 5 VG-MAS-5 | -2.62 | -60.21 | 133.9 | NA | NA | 267 | CA | TF | NA | N | Y | 2004 | Sullivan et al. (2017) | 17 |
| TEM-06 | Brazil | TEAM Manaus plot 6 VG-MAS-6 | -2.6 | -60.11 | 123.8 | NA | NA | 264 | CA | TF | NA | N | Y | 2004 | Sullivan et al. (2017) | 17 |
| YAN-01 | Peru | Yanamono A | -3.43 | -72.84 | 123.1 | NA | NA | 261 | WAN | TF | NA | N | Y | 1983 | Sullivan et al. (2017) | 17 |
| YUT-01 | Ecuador | Yutsuntsa | -2.35 | -76.43 | 121.7 | NA | NA | 226 | WAN | TF | NA | N | Y | 2006 | Sullivan et al. (2017) | 17 |
| CHBR_01 | Guyana | Charabaru 1 | 4.95 | -58.36 | 247 | 167.74 | 103 | 60 | GS | TF | NPS | Y | Y | 2003 | Hans ter Steege, Olaf Bánki | 18 |
| CHBR_02 | Guyana | Charabaru 2 | 4.95 | -58.35 | 165.5 | 48.50 | 67 | 64 | GS | PZ | NPS | Y | Y | 2003 | Hans ter Steege, Olaf Bánki | 18 |
| CHBR_03 | Guyana | Charabaru 3 | 4.95 | -58.37 | 185.4 | 35.25 | 43 | 38 | GS | PZ | NPS | Y | Y | 2003 | Hans ter Steege, Olaf Bánki | 18 |
| CHBR_04 | Guyana | Charabaru 4 | 4.92 | -58.35 | 215.7 | 141.56 | 120 | 75 | GS | TF | NPS | Y | Y | 2003 | Hans ter Steege, Olaf Bánki | 18 |
| BB_02P | Suriname | Brownsberg 2 | 4.93 | -55.19 | 222.5 | 153.28 | 68 | 138 | GS | TF | SGS | Y | Y | 2003 | Hans ter Steege, Olaf Bánki | 19 |
| BB_03P | Suriname | Brownsberg 3 | 4.95 | -55.19 | 170 | 62.69 | 72 | 136 | GS | TF | SGS | Y | Y | 2003 | Hans ter Steege, Olaf Bánki | 19 |
| BB_04M | Suriname | Brownsberg 4 | 4.97 | -55.18 | 234.8 | 257.34 | 96 | 121 | GS | TF | SGS | Y | Y | 2003 | Hans ter Steege, Olaf Bánki | 19 |
| BB_05L | Suriname | Brownsberg 5 | 4.99 | -55.2 | 189.4 | 101.63 | 149 | 126 | GS | TF | SGS | Y | Y | 2003 | Hans ter Steege, Olaf Bánki | 19 |
| BB_06M | Suriname | Brownsberg 6 | 4.94 | -55.18 | 196.7 | 139.22 | 111 | 135 | GS | TF | SGS | Y | Y | 2003 | Hans ter Steege, Olaf Bánki | 19 |
| BB_07L | Suriname | Brownsberg 7 | 4.92 | -55.13 | 130.1 | 50.93 | 235 | 125 | GS | TF | SGS | Y | Y | 2003 | Hans ter Steege, Olaf Bánki | 19 |
| BB_08L | Suriname | Brownsberg 8 | 4.93 | -55.14 | 169.6 | 69.70 | 131 | 114 | GS | TF | SGS | Y | Y | 2003 | Hans ter Steege, Olaf Bánki | 19 |
| LM_02P | Suriname | Lely Mts 02 | 4.26 | -54.74 | 248.6 | 150.64 | 102 | 136 | GS | TF | SGS | Y | Y | 2005 | Hans ter Steege, Olaf Bánki | 20 |
| LM_04M | Suriname | Lely Mts 04 | 4.25 | -54.73 | 229.7 | 149.52 | 76 | 145 | GS | TF | SGS | Y | Y | 2005 | Hans ter Steege, Olaf Bánki | 20 |
| LM_05S | Suriname | Lely Mts 05 | 4.25 | -54.73 | 125.4 | 4.26 | 6 | 33 | GS | TF | SGS | Y | Y | 2005 | Hans ter Steege, Olaf Bánki | 20 |
| LM_06L | Suriname | Lely Mts 06 | 4.26 | -54.78 | 181.1 | 140.38 | 78 | 115 | GS | TF | SGS | Y | Y | 2005 | Hans ter Steege, Olaf Bánki | 20 |
| LM_07L | Suriname | Lely Mts 07 | 4.27 | -54.78 | 182.6 | 120.43 | 106 | 107 | GS | TF | SGS | Y | Y | 2005 | Hans ter Steege, Olaf Bánki | 20 |
| LM_08M | Suriname | Lely Mts 08 | 4.27 | -54.75 | 212.6 | 156.48 | 73 | 111 | GS | TF | SGS | Y | Y | 2005 | Hans ter Steege, Olaf Bánki | 20 |
| BEI1 | French Guiana | Haute Beiman | 4.44 | -54.13 | 277 | 153.22 | 77 | 166 | GS | TF | SGS | Y | Y | 2012 | Daniel Sabatier, Jean-François Molino | 21 |
| BEI2 | French Guiana | Haute Beiman | 4.46 | -54.14 | 212.5 | 213.61 | 129 | 197 | GS | TF | SGS | Y | Y | 2012 | Daniel Sabatier, Jean-François Molino | 21 |
| CVX1 | French Guiana | Montagne des Chevaux | 4.68 | -52.36 | 211.4 | 69.17 | 100 | 123 | GS | TF | SGS | Y | Y | 2009 | Daniel Sabatier, Émile Fonty | 21 |
| PSEY | French Guiana | Piste de St Elie | 5.28 | -53.05 | 226.6 | 145.05 | 89 | 200 | GS | TF | NPS | Y | Y | 2009 | Daniel Sabatier, Émile Fonty | 21 |
| PSEZ | French Guiana | Piste de St Elie | 5.28 | -53.05 | 166.4 | 101.00 | 75 | 186 | GS | TF | NPS | Y | Y | 2009 | Daniel Sabatier, Émile Fonty | 21 |
| TORT3 | French Guiana | Crique Tortue | 4.19 | -52.44 | 185.2 | 95.53 | 64 | 204 | GS | TF | SGS | Y | Y | 2009 | Daniel Sabatier, Jean-François Molino | 21 |
| TRE1 | French Guiana | Montagne de Kaw, Trésor | 4.61 | -52.28 | 214.6 | 134.82 | 40 | 161 | GS | TF | SGS | Y | Y | 2003 | Daniel Sabatier, Hans ter Steege, Jean-François Molino, Marie-Françoise Prévost | 21 |
| GURI_03 | Venezuela | Guri, Cola | 7.24 | -62.84 | 76.3 | 6.79 | 103 | 39 | GS | TF | NWGS | Y | Y | 2010 | John Terborgh, Kenneth Feeley, Miles Sillman | 22 |
| NWG_05 | Guyana | Santa Rosa, Moruca River Kabrora, mixed primary forest | 7.6 | -58.95 | 246.8 | 165.05 | 54 | 87 | GS | TF | NWGS | Y | Y | 1997 | Tinde van Andel | 23 |
| NWG_03 | Guyana | Kariako, Barama River mixed primary forest | 7.42 | -59.73 | 227.5 | 183.02 | 43 | 83 | GS | TF | NWGS | Y | Y | 1996 | Tinde van Andel | 24 |
| CAM1 | French Guiana | Rivière Camopi, crique Cacao | 2.36 | -53.19 | 198.2 | 142.36 | 137 | 144 | GS | TF | SGS | Y | Y | 1987 | Daniel Sabatier, Jean-François Molino | 14, 15 |

1 Bánki, O. S. (2006) Tree diversity in the vicinity of Kabo: A field report. Ecology & Biodiversity, Utrecht University.

2 Bánki, O. S. (2006) Tree diversity plots in the vicinity of Bitagron: A field report. Ecology & Biodiversity, Utrecht University.

3 Bánki, O. S. (2006) Tree diversity plots on white sand in the Bruynzeel Suriname Houtmaatschappij Kabo Concession: A field report. Ecology & Biodiversity, Utrecht University.

4 Bánki, O. S. (2010) Does neutral theory explain community composition in the Guiana Shield forests? PhD Thesis, Dept. Biology, Utrecht University.

5 Bánki, O. S., ter Steege, H., Jansen-Jacobs, M. J. & Raghoenandan, U. P. D. (2003) Plant diversity of the Nassau Mountains Suriname. Report of the 2003 Expedition. NHN-Utrecht, BBS-Paramaribo.

6 Castellanos unpublished data

7 Fonty, É., Molino, J.-F., Prévost, M.-F. & Sabatier, D. (2011) A new case of neotropical monodominant forest: Spirotropis longifolia (Leguminosae-Papilionoideae) in French Guiana. Journal of Tropical Ecology, 27, 641-644.

8 Forestplot.net

9 Kalamandeen et al unpubl.

10 Linares-Palomino, R. and Wortel, V., 2015-10-28, Vegetation - Trees & Lianas Metadata Version 1.5, VT-20180521030329_3993

10 Lopez-Gonzalez, G., Lewis, S. L., Burkitt, M., T.R., B. & Phillips, O. L. (2009) ForestPlots.net Database. Www.forestplots.net.

11 Nicolás Castaño Arboleda, unpublished data

12 Pélissier, R., Dray, S. & Sabatier, D. (2002) Within-plot relationships between tree species occurrences and hydrological soil constraints: an example in French Guiana investigated through canonical correlation analysis. Plant Ecology, 162, 143-156.

13 Ruysschaert, S. (2018). Non-timber forest products in Suriname : diversity, knowledge and use of plants in an Ameridian and Maroon community. Ghent University. Faculty of Bioscience Engineering, Ghent, Belgium.

14 Sabatier, D. & Molino, J.-F. unpublished data

15 Sabatier, D. & Prévost, M. F. (1987) Une forêt a cacaoyers sauvages sur le haut-camopi, en Guyane Française. Cayenne.

16 Stropp, J., Sleen, P.V., Assunção, P.A., Silva, A.L. & Steege, H.T. (2011) Tree communities of white-sand and terra-firme forests of the upper rio negro. Acta Amazonica, 41, 521-544

17 Sullivan MJP, Talbot J, Lewis SL, Phillips OL, Qie L, Begne SK, Chave J, Cuni Sanchez A, Hubau W, Lopez-Gonzalez G, Miles L, Monteagudo-Mendoza A, Sonké B, Sunderland T, ter Steege H, White LJT, Affum-Baffoe K, Aiba S, Almeida EC, Almeida de Oliveira E, Alvarez-Loayza P, Álvarez Dávila E, Andrade A, Aragão LEOC, Ashton P, Aymard C. GA, Baker TR, Balinga M, Banin LF, Baraloto C, Bastin J-F, Berry N, Bogaert J, Bonal D, Bongers F, Brienen R, Camargo JLC, Cerón C, Chama Moscoso V, Chezeaux E, Clark CJ, Cogollo Pacheco A, Comiskey JA, Cornejo Valverde F, Honorio Coronado E, Dargie G, Davies SJ, De Canniere C, Djuikouo K. MN, Doucet J-L, Erwin TL, Espejo JS, Ewango CEN, Fauset S, Feldpausch TR, Herrera R, Gilpin M, Gloor E, Hall J, Harris DJ, Hart TB, Kartawinata K, Khoon Kho L, Kitayama K, Laurance SGW, Laurance WF, Leal ME, Lovejoy T, Lovett J, Lukasu FM, Makana JR, Malhi Y, Maracahipes L, Marimon BS, Marimon BH, Marshall AR, Morandi PS, Mukendi JT, Mukinzi J, Nilus R, Núñez Vargas P, Pallqui Camacho NC, Pardo G, Peña-Claros M, Pétronelli P, Pickavance GC, Poulsen AD, Poulsen JR, Primack RB, Priyadi H, Quesada CA, Reitsma J, Réjou-Méchain M, Restrepo Z, Rutishauser E, Salim KA, Salomão RP, Samsoedin I, Sheil D, Sierra R, Silveira M, Slik JWF, Steel L, Taedoumg H, Tan S, Terborgh JW, Thomas SC, Toledo M, Umunay P, Valenzuela Gamarra L, Vieira ICG, Vos VA, Wang O, Willcock S, Zemagho L. Data from Diversity and carbon storage across the tropical forest biome. ForestPlots.net.

18 ter Steege, H. (2004) Tree diversity plots in the Charabaru creek area, upper Berbice, Guyana. NHN-Utrecht., Utrecht.

19 ter Steege, H. B., O. S., van Andel, T. R., Behari-Ramdas, J., Ramharakh, G. (2004b) Plant diversity in the Brownsberg Nature Park, Suriname. Report of the Nov-Dec 2003 expedition. NHN-Utrecht, BBS-Paramaribo, Utrecht.

20 ter Steege, H., Bánki, O.S., Jansen-Jacobs, M., Ramharakh, G. & Tjon, K. (2005 ) Plant diversity of Lely Mountains, Suriname. Report of the Nov-Dec 2004 Expedition. NHN-Utrecht, BBS-Paramaribo. .

21 ter Steege, H., Sebastier, D., Molino, J. F., Bánki, O., Prévost F, M., Pelissier, R. . (2003) Report of the establishment of a permanent one-hectare plot in Réserve Naturelle Volontarie Trésor. Unpublished.

22 Terborgh, J., K. Feeley, M. R. Silman, P. Nuñez V, and N. Balukjan. 2006. Vegetation dynamics of predator-free land-bridge islands. Journal of Ecology 94:253–263.

23 van Andel, T. R. (2003) Floristic composition and diversity of three swamp forests in northwest Guyana. Plant Ecology, 167, 293-317.

24 van Andel, T.R. (2001) Floristic composition and diversity of mixed primary and secondary forests in northwest Guyana. Biodiversity and Conservation, 10, 1645-1682.

### Annex 2: Supplementary results

#### Guiana Shield linear relationships across environmental covariables

**Table S2.1**. Detailed results for the bivariate linear models of the three ecosystem services of aboveground carbon stock, timber stock and NTFP abundance predicted by woody species richness across the Guiana Shield dataset (n = 151). Showing model coefficients with its standard error (SE), the t-test value, the p -value of the t-test (H0: b = 0), and the R^2^ of the total model.

| Model | | Coefficient | SE | t-value | p-value | R^2^ |
| --- | --- | --- | --- | --- | --- | --- |
| Model AGC ~ Spp | |  |  |  |  |  |
|  | Intercept | 163.68276 | 9.86965 | 16.584 | <2.00E-16 |  |
|  | Species richness | 0.39147 | 0.07381 | 5.304 | 4.03E-07 |  |
|  |  |  |  |  |  | 0.1588 |
| Model Timber ~ Spp | |  |  |  |  |  |
|  | Intercept | 48.58106 | 13.33746 | 3.642 | 0.000372 |  |
|  | Species richness | 0.57467 | 0.09974 | 5.761 | 4.62E-08 |  |
|  |  |  |  |  |  | 0.1822 |
| Model NTFP ~ Spp | |  |  |  |  |  |
|  | Intercept | 104.80933 | 12.59899 | 8.319 | 5.19E-14 |  |
|  | Species richness | -0.01695 | 0.09422 | -0.18 | 0.857 |  |
|  |  |  |  |  |  | 0.0002172 |

**Table S2.2.** Detailed results for the multivariate linear models of the three ecosystem services of aboveground carbon stock, timber stock and NTFP abundance predicted by woody species richness and either biogeographical subregion or forest types across the Guiana Shield dataset (n = 151, 1-ha plots). Showing model coefficients with its standard error (SE), the t-test value, the p -value of the t-test (H0: b = 0), and the R^2^ of the total model. Forest types included were: TF = terra firme forests (n = 130) and PZ = white sand forests (n = 21). Biogeographical subregions included were: SGS = forests of the Southern Guiana Shield (n = 63), NWGS = forests of the North-Western Guiana Shield (n = 21), NPS = forests of the Northern Pleistocene sands (n = 56) and SWPS = forests of the South-Western Pleistocene sands in the upper Rio Negro region (n = 11).

| Model | | | Coefficient  estimate | Coefficient  SE | t-value | t-test  p-value | Rel. contr. R^2^ | R^2^ |
| --- | --- | --- | --- | --- | --- | --- | --- | --- |
| Carbon stock | | |  |  |  |  |  |  |
|  | Species richness | | 0.34971 | 0.07527 | 4.646 | 7.48E-06 | 0.1286891 |  |
|  | Biogeographical subregion | |  |  |  |  | 0.2046719 |  |
|  |  | SGS (intercept) | 163.91438 | 12.16237 | 13.477 | <2.00E-16 |  |  |
|  |  | NWGS | -23.26744 | 11.63995 | -1.999 | 0.047472 |  |  |
|  |  | NPS | 28.46651 | 7.81838 | 3.641 | 0.000376 |  |  |
|  |  | SWPS | -32.6318 | 13.52743 | -2.412 | 0.017095 |  |  |
|  |  |  |  |  |  |  |  | 0.3334 |
| Carbon stock | | |  |  |  |  |  |  |
|  | Species richness | | 0.54094 | 0.08191 | 6.604 | 6.74E-10 | 0.19310891 |  |
|  | Forest type | |  |  |  |  | 0.03514065 |  |
|  |  | TF (Intercept) | 139.12729 | 11.62969 | 11.963 | <2.00E-16 |  |  |
|  |  | PZ | 43.36936 | 11.88436 | 3.649 | 0.000364 |  |  |
|  |  |  |  |  |  |  |  | 0.2282 |
| Timber stock | | |  |  |  |  |  |  |
|  | Species richness | | 0.4159 | 0.1041 | 3.995 | 1.02E-04 | 0.1282011 |  |
|  | Biogeographical subregion | |  |  |  |  | 0.1930405 |  |
|  |  | SGS (intercept) | 89.6015 | 16.8199 | 5.327 | 3.70E-07 |  |  |
|  |  | NWGS | -47.7668 | 16.0974 | -2.967 | 0.003511 |  |  |
|  |  | NPS | -20.6756 | 10.8124 | -1.912 | 0.057807 |  |  |
|  |  | SWPS | -96.5015 | 18.7077 | -5.158 | 7.99E-07 |  |  |
|  |  |  |  |  |  |  |  | 0.3212 |
| Timber stock | | |  |  |  |  |  |  |
|  | Species richness | | 0.4474 | 0.1137 | 3.936 | 0.000127 | 0.13250222 |  |
|  | Forest type | |  |  |  |  | 0.07649242 |  |
|  |  | TF (Intercept) | 69.4906 | 16.1366 | 4.306 | 3.01E-05 |  |  |
|  |  | PZ | -36.93 | 16.49 | -2.24 | 0.026612 |  |  |
|  |  |  |  |  |  |  |  | 0.209 |
| NTFP abundance | | |  |  |  |  |  |  |
|  | Species richness | | -0.10557 | 0.09929 | -1.063 | 0.2894 | 0.003384304 |  |
|  | Biogeographical subregion | |  |  |  |  | 0.150451398 |  |
|  |  | SGS (intercept) | 125.51139 | 16.04461 | 7.82E+00 | 9.54E-13 |  |  |
|  |  | NWGS | -29.57142 | 15.35544 | -1.926 | 0.0561 |  |  |
|  |  | NPS | 1.22552 | 10.31402 | 1.19E-01 | 0.9056 |  |  |
|  |  | SWPS | -83.20705 | 17.84541 | -4.663 | 6.98E-06 |  |  |
|  |  |  |  |  |  |  |  | 0.1538 |
| NTFP abundance | | |  |  |  |  |  |  |
|  | Species richness | | -0.06197 | 0.10892 | -0.569 | 0.57 | 0.001196909 |  |
|  | Forest type | |  |  |  |  | 0.003614758 |  |
|  |  | TF (Intercept) | 112.20508 | 15.46363 | 7.256 | 2.08E-11 |  |  |
|  |  | PZ | -13.06222 | 15.80226 | -0.827 | 0.41 |  |  |
|  |  |  |  |  |  |  |  | 0.004812 |

**Table S2.3**. Detailed results for the optimized linear models of the three ecosystem services of carbon stock, timber stock and NTFP abundance predicted by species richness and environmental covariables across the Guiana Shield dataset (n = 151, 1-ha plots). Originally included predictors were species richness, forest type, biogeographical subregion, latitude and longitude. Forest types included were: TF = terra firme forests (n = 130) and PZ = white sand forests (n = 21). Biogeographical subregions included were: SGS = forests of the Southern Guiana Shield (n = 63), NWGS = forests of the North-Western Guiana Shield (n = 21), NPS = forests of the Northern Pleistocene sands (n = 56) and SWPS = forests of the South-Western Pleistocene sands in the upper Rio Negro region (n = 11). Showing model coefficients (b) with their standard error (SE), t-test value, p -value of the t-test (H0: b = 0) and relative contribution to total R^2^; and total model R^2^.

| Model | | | Coefficient  b | Coefficient  SE | t-value | t-test  p-value | Rel. contr. R^2^ | R^2^ |
| --- | --- | --- | --- | --- | --- | --- | --- | --- |
| Carbon stock | | |  |  |  |  |  |  |
|  | Intercept (TF & SGS) | | 142.04436 | 14.18498 | 10.014 | <2.00E-16 |  |  |
|  | Species richness | | 0.49916 | 0.09061 | 5.509 | 1.60E-07 | 0.15135157 |  |
|  | Forest type | |  |  |  |  | 0.02360114 |  |
|  |  | PZ | 36.98588 | 13.10284 | 2.823 | 0.00543 |  |  |
|  | Biogeographical subregion | |  |  |  |  | 0.1931324 |  |
|  |  | NWGS | -12.57085 | 11.98652 | -1.049 | 2.96E-01 |  |  |
|  |  | NPS | 22.14163 | 7.96011 | 2.782 | 0.00613 |  |  |
|  |  | SWPS | -45.50795 | 13.98085 | -3.255 | 0.00141 |  |  |
|  |  |  |  |  |  |  |  | 0.3681 |
| Timber stock | | |  |  |  |  |  |  |
|  | Intercept (TF & SGS) | | 111.4114 | 19.8741 | 5.606 | 1.01E-07 |  |  |
|  | Species richness | | 0.2668 | 0.127 | 2.102 | 0.037294 | 0.09360782 |  |
|  | Forest type | |  |  |  |  | 0.06473521 |  |
|  |  | PZ | -36.8843 | 18.3579 | -2.009 | 0.046375 |  |  |
|  | Biogeographical subregion | |  |  |  |  | 0.18128327 |  |
|  |  | NWGS | -58.434 | 16.7939 | -3.479 | 0.000664 |  |  |
|  |  | NPS | -14.3681 | 11.1526 | -1.288 | 0.199687 |  |  |
|  |  | SWPS | -83.6607 | 19.5881 | -4.271 | 3.50E-05 |  |  |
|  |  |  |  |  |  |  |  | 0.3396 |
| NTFP abundance | | |  |  |  |  |  |  |
|  | Biogeographical subregion | |  |  |  |  | 0.1473 |  |
|  |  | SGS (intercept) | 110.063 | 6.809 | 16.164 | <2.00E-16 |  |  |
|  |  | NWGS | -22.016 | 13.618 | -1.617 | 0.108 |  |  |
|  |  | NPS | 4.222 | 9.926 | 0.425 | 6.71E-01 |  |  |
|  |  | SWPS | -80.427 | 17.661 | -4.554 | 1.10E-05 |  |  |
|  |  |  |  |  |  |  |  | 0.1473 |

**Table S2.4**. Detailed results for the bivariate linear models of the three ecosystem services of aboveground carbon stock (AGB), timber stock (timber) and NTFP abundance (NTFP) predicted by woody species richness, aggregated and separate per biogeographical subregion, across the Guiana Shield dataset (n = 151). Showing model coefficients with its standard error (SE), the t-test value, the p -value of the t-test (H0: b = 0), and the R^2^ of the total model.

| Model - subset | | | Coefficient | SE | t-value | p-value | R^2^ |
| --- | --- | --- | --- | --- | --- | --- | --- |
| Model AGC ~ Spp | | |  |  |  |  |  |
|  | Aggregated (n = 151) | |  |  |  |  |  |
|  |  | Intercept | 163.68276 | 9.86965 | 16.584 | <2.00E-16 |  |
|  |  | Species richness | 0.39147 | 0.07381 | 5.304 | 4.03E-07 |  |
|  |  |  |  |  |  |  | 0.1588 |
|  | SGS (n = 63) | |  |  |  |  |  |
|  |  | Intercept | 135.3323 | 18.7655 | 7.212 | 1.00E-09 |  |
|  |  | Species richness | 0.545 | 0.1246 | 4.375 | 4.82E-05 |  |
|  |  |  |  |  |  |  | 0.2389 |
|  | NWGS (n = 21) | |  |  |  |  |  |
|  |  | Intercept | 33.5981 | 48.7157 | 0.69 | 0.4987 |  |
|  |  | Species richness | 1.7816 | 0.6427 | 2.772 | 1.21E-02 |  |
|  |  |  |  |  |  |  | 0.2879 |
|  | NPS (n = 56) | |  |  |  |  |  |
|  |  |  | 202.9563 | 14.1427 | 14.351 | <2e-16 |  |
|  |  |  | 0.26 | 0.1071 | 2.427 | 0.0186 |  |
|  |  |  |  |  |  |  | 0.09835 |
|  | SWPS (n = 11) | |  |  |  |  |  |
|  |  |  | 153.2535 | 32.0558 | 4.781 | 0.001 |  |
|  |  |  | 0.1666 | 0.255 | 0.653 | 0.53 |  |
|  |  |  |  |  |  |  | 0.04528 |
| Model Timber ~ Spp | | |  |  |  |  |  |
|  | Aggregated (n = 151) | |  |  |  |  |  |
|  |  | Intercept | 48.58106 | 13.33746 | 3.642 | 0.000372 |  |
|  |  | Species richness | 0.57467 | 0.09974 | 5.761 | 4.62E-08 |  |
|  |  |  |  |  |  |  | 0.1822 |
|  | SGS (n = 63) | |  |  |  |  |  |
|  |  | Intercept | 107.871 | 34.4695 | 3.129 | 0.00269 |  |
|  |  | Species richness | 0.291 | 0.2288 | 1.272 | 0.20822 |  |
|  |  |  |  |  |  |  | 0.02584 |
|  | NWGS (n = 21) | |  |  |  |  |  |
|  |  | Intercept | 22.0379 | 62.3417 | 0.354 | 0.728 |  |
|  |  | Species richness | 0.6807 | 0.8225 | 0.828 | 0.418 |  |
|  |  |  |  |  |  |  | 0.03479 |
|  | NPS (n = 56) | |  |  |  |  |  |
|  |  |  | 56.876 | 15.182 | 3.746 | 0.000438 |  |
|  |  |  | 0.518 | 0.115 | 4.504 | 3.61E-05 |  |
|  |  |  |  |  |  |  | 0.2731 |
|  | SWPS (n = 11) | |  |  |  |  |  |
|  |  |  | 77.8414 | 43.6885 | 1.782 | 0.108 |  |
|  |  |  | -0.2903 | 0.3476 | -0.835 | 0.425 |  |
|  |  |  |  |  |  |  | 0.07194 |
| Model NTFP ~ Spp | | |  |  |  |  |  |
|  | Aggregated (n = 151) | |  |  |  |  |  |
|  |  | Intercept | 104.80933 | 12.59899 | 8.319 | 5.19E-14 |  |
|  |  | Species richness | -0.01695 | 0.09422 | -0.18 | 0.857 |  |
|  |  |  |  |  |  |  | 0.0002172 |
|  | SGS (n = 63) | |  |  |  |  |  |
|  |  | Intercept | 102.62274 | 24.51845 | 4.186 | 9.28E-05 |  |
|  |  | Species richness | 0.05085 | 0.16275 | 0.312 | 0.756 |  |
|  |  |  |  |  |  |  | 0.001598 |
|  | NWGS (n = 21) | |  |  |  |  |  |
|  |  | Intercept | 82.43355 | 42.10075 | 1.958 | 0.0651 |  |
|  |  | Species richness | 0.07509 | 0.55546 | 0.135 | 0.8939 |  |
|  |  |  |  |  |  |  | 0.000961 |
|  | NPS (n = 56) | |  |  |  |  |  |
|  |  |  | 136.5336 | 21.0638 | 6.482 | 2.85E-08 |  |
|  |  |  | -0.1886 | 0.1596 | -1.182 | 0.242 |  |
|  |  |  |  |  |  |  | 0.02522 |
|  | SWPS (n = 11) | |  |  |  |  |  |
|  |  |  | 18.05263 | 15.1741 | 1.19 | 0.265 |  |
|  |  |  | 0.09653 | 0.12072 | 0.8 | 0.445 |  |
|  |  |  |  |  |  |  | 0.06633 |


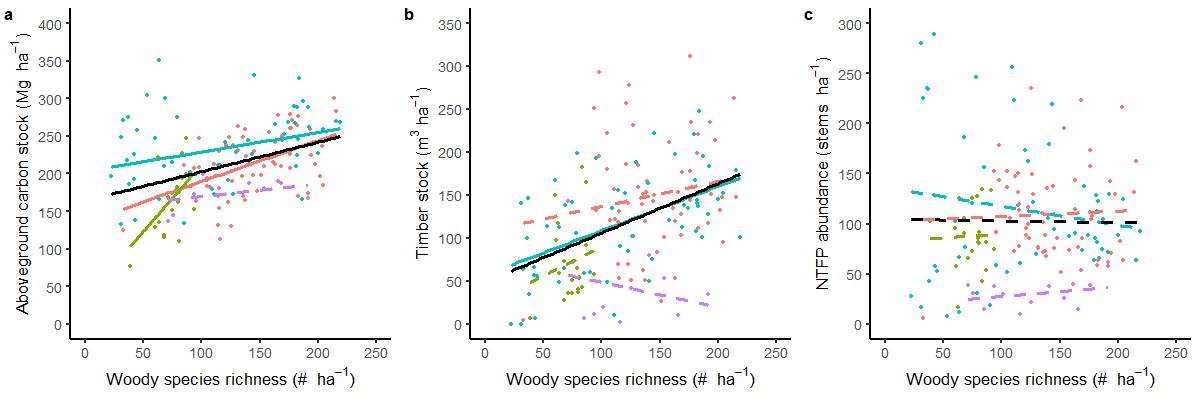


**Figure S2.1**. Visualisation of linear bivariate relationships between carbon stock and species richness (panel a), between timber stock and species richness (panel b) and between NTFP abundance and species richness (panel c) for each of the four subregions of the Guiana Shield separately (different colours), and aggregated across all subregions (black line). Colours according to the subregion of the Guiana Shield: Forests of the Southern Guiana Shield (SGS, red, n = 63), Forests of the North-Western Guiana Shield (NWGS, green, n = 21), Forests of the Northern Pleistocene sands (NPS, turquoise, n = 56) and Forests of the South-Western Pleistocene sands in the upper Rio Negro region (SWPS, purple, n = 11). Solid lines indicate significant relationships, p < 0.05, and dashed lines non-significant relationships. Model details included in Table S2.4.

**Table S2.5**. Analysis of variance F-tests for aboveground carbon stock, timber stock, NTFP abundance and woody species richness across ~ biogeographical subregions in the Guiana Shield dataset (4 subregions, n = 151).

| **Model** | **F-statistic** | **Df1** | **Df2** | **p-value** | **Multiple R-squared** |
| --- | --- | --- | --- | --- | --- |
| Carbon stock ~ Biogeographical subregion | 15.03 | 3 | 147 | 1.38E-08 | 0.2348 |
| Timber stock ~ Biogeographical subregion | 16.08 | 3 | 147 | 4.326e-09 | 0.247 |
| NTFP abundance ~ Biogeographical subregion | 8.463 | 3 | 147 | 3.177e-05 | 0.1473 |
| Species richness ~ Biogeographical subregion | 14.0 | 3 | 147 | 4.467e-08 | 0.2222 |

**Table S2.6**. Summary of post-hoc Tukey contrasts of aboveground carbon stock, timber stock, NTFP abundance and woody species richness across biogeographical subregions in the Guiana Shield dataset (4 subregions, n = 151). Simultaneous Tests for General Linear Hypotheses. Showing adjusted p values of single-step method.

| **Linear Hypothesis** | | **Estimate** | **SE** | **t-value** | **Adjusted p-value** |
| --- | --- | --- | --- | --- | --- |
| Carbon stock ~ Biogeographical subregion | |  |  |  |  |
|  | H_0_: NWGS - SGS = 0 | -48.296 | 11.017 | -4.384 | <0.001 |
|  | H_0_: NPS - SGS = 0 | 18.539 | 8.03 | 2.309 | 0.095 |
|  | H_0_: SWPS - SGS = 0 | -41.841 | 14.288 | -2.928 | 0.0192 |
|  | H_0_: NPS - NWGS = 0 | 66.836 | 11.188 | 5.974 | <0.001 |
|  | H_0_: SWPS - NWGS = 0 | 6.456 | 16.274 | 0.397 | 0.9777 |
|  | H_0_: SWPS - NPS= 0 | -60.38 | 14.42 | -4.187 | <0.001 |
| Timber stock ~ Biogeographical subregion | |  |  |  |  |
|  | H_0_: NWGS - SGS = 0 | -77.53 | 14.98 | -5.176 | <0.001 |
|  | H_0_: NPS - SGS = 0 | -32.48 | 10.92 | -2.975 | 0.01696 |
|  | H_0_: SWPS - SGS = 0 | -107.45 | 19.42 | -5.532 | <0.001 |
|  | H_0_: NPS - NWGS = 0 | 45.05 | 15.21 | 2.962 | 0.01698 |
|  | H_0_: SWPS - NWGS = 0 | -29.92 | 22.12 | -1.352 | 0.51838 |
| NTFP abundance ~ Biogeographical subregion | |  |  |  |  |
|  | H_0_: NWGS - SGS = 0 | -22.016 | 13.618 | -1.617 | 0.3598 |
|  | H_0_: NPS - SGS = 0 | 4.222 | 9.926 | 0.425 | 0.9727 |
|  | H_0_: SWPS - SGS = 0 | -80.427 | 17.661 | -4.554 | <0.001 |
|  | H_0_: NPS - NWGS = 0 | 26.238 | 13.829 | 1.897 | 0.2232 |
|  | H_0_: SWPS - NWGS = 0 | -58.411 | 20.115 | -2.904 | 0.0201 |
| Species richness ~ Biogeographical subregion | |  |  |  |  |
|  | H_0_: NWGS - SGS = 0 | -71.571 | 11.307 | -6.33 | <0.001 |
|  | H_0_: NPS - SGS = 0 | -28.387 | 8.242 | -3.444 | 0.00392 |
|  | H_0_: SWPS - SGS = 0 | -26.333 | 14.664 | -1.796 | 0.26819 |
|  | H_0_: NPS - NWGS = 0 | 43.185 | 11.483 | 3.761 | 0.00118 |
|  | H_0_: SWPS - NWGS = 0 | 45.238 | 16.702 | 2.709 | 0.03503 |


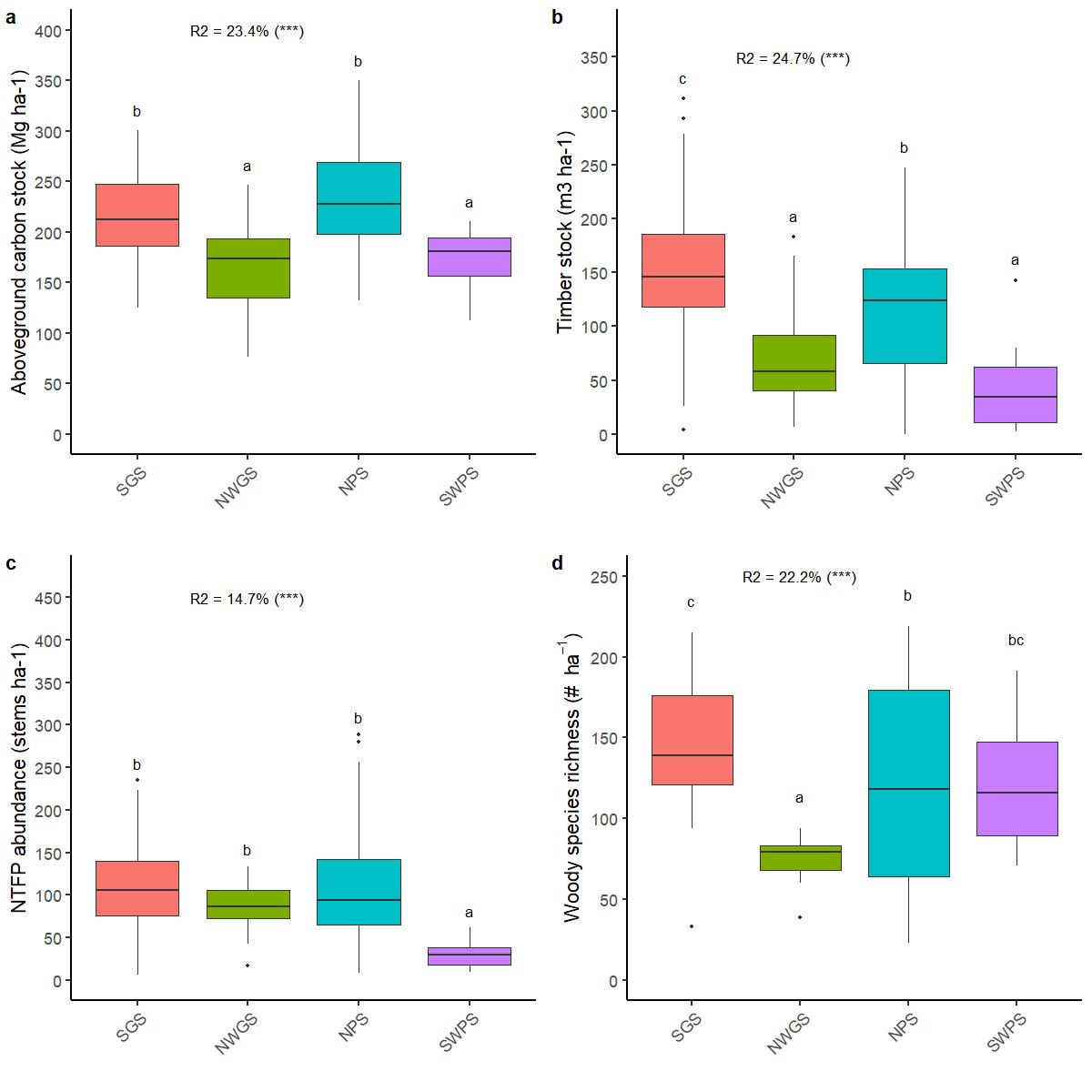


**Figure S2.2.** Box plots of mean aboveground carbon stock (Panel a), timber stock (Panel b), NTFP abundance (Panel c) and species richness (Panel d), across four subregions of the Guiana Shield. R2 is given, and the p-value rank of the F-test is given between parentheses. P-value ranks: p < 0.001 (***), p < 0.01 (**), p < 0.05 (*), p ≥ 0.05 (ns). Significance differences between the means on the basis of Post-hoc Tukey Contrasts are indicated by unique letter combinations. Subregion: Forests of the Southern Guiana Shield (SGS, red, n = 63), Forests of the North-Western Guiana Shield (NWGS, green, n = 21), Forests of the Northern Pleistocene sands (NPS, turquoise, n = 56) and Forests of the South-Western Pleistocene sands (SWPS, purple, n = 11). F-test values and Tukey contrasts are included in Tables S2.5-S2.6.

**Table S2.7**. Detailed results for the bivariate linear models of the three ecosystem services of aboveground carbon stock, timber stock and NTFP abundance predicted by woody species richness, aggregated and separate per forest type, across the Guiana Shield dataset (n = 151). Showing model coefficients with its standard error (SE), the t-test value, the p -value of the t-test (H0: b = 0), and the R^2^ of the total model.

| Model - subset | | | Coefficient | SE | t-value | p-value | R^2^ |
| --- | --- | --- | --- | --- | --- | --- | --- |
| Model AGC ~ Spp | | |  |  |  |  |  |
|  | Aggregated (n = 151) | |  |  |  |  |  |
|  |  | Intercept | 163.68276 | 9.86965 | 16.584 | <2.00E-16 |  |
|  |  | Species richness | 0.39147 | 0.07381 | 5.304 | 4.03E-07 |  |
|  |  |  |  |  |  |  | 0.1588 |
|  | TF (n = 130) | |  |  |  |  |  |
|  |  | Intercept | 127.51249 | 10.78668 | 11.821 | <2.00E-16 |  |
|  |  | Species richness | 0.6276 | 0.07634 | 8.221 | 1.94E-13 |  |
|  |  |  |  |  |  |  | 0.3456 |
|  | PZ (n = 21) | |  |  |  |  |  |
|  |  | Intercept | 238.4232 | 26.4577 | 9.011 | 2.74E-08 |  |
|  |  | Species richness | -0.3688 | 0.3756 | -0.982 | 0.339 |  |
|  |  |  |  |  |  |  | 0.04829 |
| Model Timber ~ Spp | | |  |  |  |  |  |
|  | Aggregated (n = 151) | |  |  |  |  |  |
|  |  | Intercept | 48.58106 | 13.33746 | 3.642 | 0.000372 |  |
|  |  | Species richness | 0.57467 | 0.09974 | 5.761 | 4.62E-08 |  |
|  |  |  |  |  |  |  | 0.1822 |
|  | TF (n = 130) | |  |  |  |  |  |
|  |  | Intercept | 58.2685 | 16.6812 | 3.493 | 0.000656 |  |
|  |  | Species richness | 0.5311 | 0.1181 | 4.499 | 1.51E-05 |  |
|  |  |  |  |  |  |  | 0.1365 |
|  | PZ (n = 21) | |  |  |  |  |  |
|  |  | Intercept | 86.5963 | 25.0805 | 3.453 | 0.00267 |  |
|  |  | Species richness | -0.4316 | 0.3561 | -1.212 | 0.24035 |  |
|  |  |  |  |  |  |  | 0.07177 |
| Model NTFP ~ Spp | | |  |  |  |  |  |
|  | Aggregated (n = 151) | |  |  |  |  |  |
|  |  | Intercept | 104.80933 | 12.59899 | 8.319 | 5.19E-14 |  |
|  |  | Species richness | -0.01695 | 0.09422 | -0.18 | 8.57E-01 |  |
|  |  |  |  |  |  |  | 0.0002172 |
|  | TF (n = 131) | |  |  |  |  |  |
|  |  | Intercept | 96.25597 | 13.42427 | 7.17 | 5.32E-11 |  |
|  |  | Species richness | 0.05704 | 0.095 | 0.6 | 5.49E-01 |  |
|  |  |  |  |  |  |  | 0.002808 |
|  | PZ (n = 21) | |  |  |  |  |  |
|  |  | Intercept | 175.9396 | 40.7178 | 4.321 | 0.000368 |  |
|  |  | Species richness | -1.3112 | 0.5781 | -2.27E+00 | 0.035165 |  |
|  |  |  |  |  |  |  | 0.2131 |


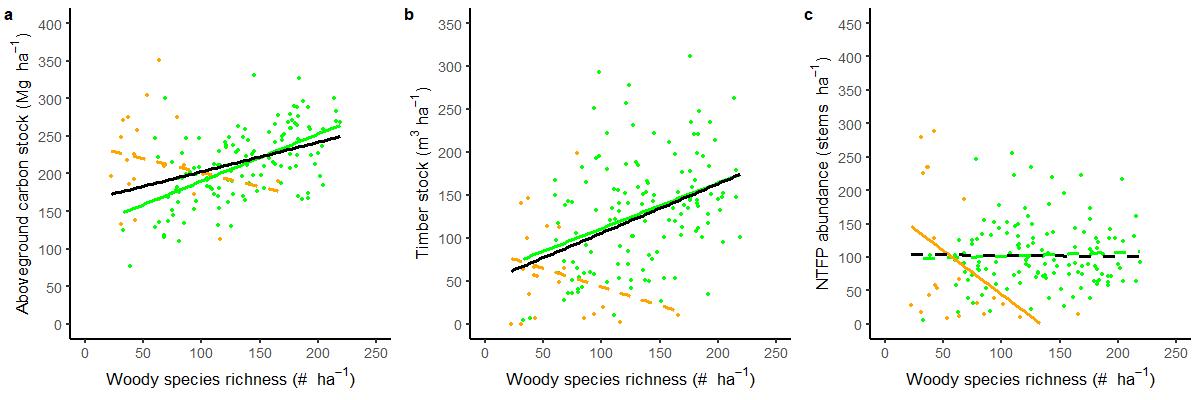


**Figure S2.3**. Visualisation of linear bivariate relationships between ecosystem services and species richness for different forest types and across all forest types for 151 1-ha tropical forest plots of the Guiana Shield. Showing relationships between carbon stock and species richness (panel a), timber stock and species richness (panel b), and NTFP abundance and species richness (panel c). Relationships across all forest types (aggregated): black lines; relationships for terra firme forests alone (TF, n = 130): green lines; relationships for white sand forests alone: orange lines (PZ, n = 21). Here, solid lines for p < 0.05 and dashed lines for p ≥ 0.05. Forest plots coloured to forest types: terra firme forests: green; white sand forests: orange. Model details included in Table S2.7.

**Table S2.8**. Analysis of variance F-tests for aboveground carbon stock, timber stock, NTFP abundance and woody species richness across forest types in the Guiana Shield dataset (2 forest types, n = 151).

| **Model** | **F-statistic** | **Df1** | **Df2** | **p-value** | **Multiple R-squared** |
| --- | --- | --- | --- | --- | --- |
| Carbon stock ~ Forest type | 0.125 | 1 | 149 | 0.7242 | 0.0008381 |
| Timber stock ~ Forest type | 21.52 | 1 | 149 | 7.622e-06 | 0.1262 |
| NTFP abundance ~ Forest type | 0.3937 | 1 | 149 | 0.5313 | 0.002635 |
| Species richness ~ Forest type | 49.67 | 1 | 149 | 6.287e-11 | 0.25 |

**Table S2.9.** Summary of post-hoc Tukey contrasts of aboveground carbon stock, timber stock, NTFP abundance and woody species richness across forest types in the Guiana Shield dataset (2 forest types, n = 151). Simultaneous Tests for General Linear Hypotheses. Showing adjusted p values of single-step method.

| **Linear Hypothesis** | | **Estimate** | **SE** | **t-value** | **Adjusted p-value** |
| --- | --- | --- | --- | --- | --- |
| Carbon stock ~ Forest type | |  |  |  |  |
|  | H_0_: PZ - TF = 0 | 4.126 | 11.671 | 0.354 | 0.724 |
| Timber stock ~ Forest type | |  |  |  |  |
|  | H_0_: PZ - TF = 0 | -69.39 | 14.96 | -4.638 | 7.62E-06 |
| NTFP abundance ~ Forest type | |  |  |  |  |
|  | H_0_: PZ - TF = 0 | -8.567 | 13.654 | -0.627 | 5.31E-01 |
| Species richness ~ Forest type | |  |  |  |  |
|  | H_0_: PZ - TF = 0 | -72.55 | 10.29 | -7.048 | 6.29E-11 |


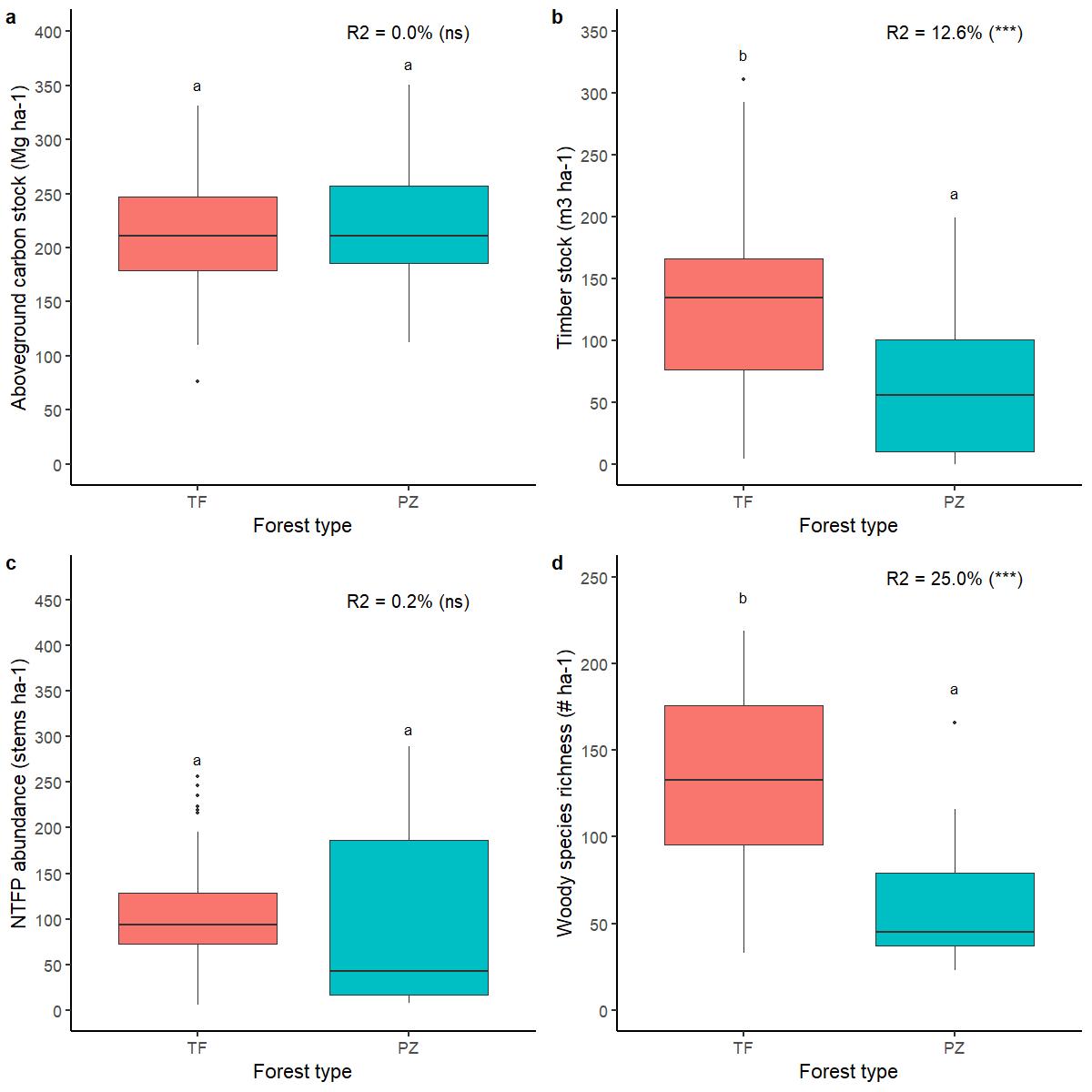


**Figure S2.4.** Box plots of mean aboveground carbon stock (Panel a), mean timber stock (Panel b), mean NTFP abundance (Panel c) and mean woody species richness (Panel d) per forest type across the Guiana Shield dataset (n = 151). R^2^ is given, and the p-value rank of the F-test is given between parentheses. P-value ranks: p < 0.001 (***), p < 0.01 (**), p < 0.05 (*), p ≥ 0.05 (ns). Significance differences between the means on the basis of Post-hoc Tukey Contrasts are indicated by unique letter combinations. Forest types were: TF = terra firme forests (n = 130) and PZ = white sand forests (n = 21). F-test values and Tukey contrasts are included in Tables S2.8-2.9.

#### Amazonia linear relationships across environmental covariables

**Table S2.10**. Detailed results for the bivariate linear models of the three ecosystem services of aboveground carbon stock, timber stock and NTFP abundance predicted by woody species richness across the Amazonia dataset (n = 283). Showing model coefficients with its standard error (SE), the t-test value, the p -value of the t-test (H0: b = 0), and the R^2^ of the total model.

| Model | | Coefficient | SE | t-value | p-value | R^2^ |
| --- | --- | --- | --- | --- | --- | --- |
| Carbon stock | |  |  |  |  |  |
|  | Intercept | 176.390492 | 8.679143 | 20.323 | <2e-16 |  |
|  | Species richness | -0.007433 | 0.056021 | -0.133 | 0.8950 |  |
|  |  |  |  |  |  | 6.264e-05 |

**Table S2.11**. Detailed results for the optimized linear models of aboveground carbon stock predicted by woody species richness and environmental covariables across the Amazonia dataset (n = 283). Originally included predictors were species richness, biogeographical region, forest type, latitude and longitude. Showing model coefficients with its standard error (SE), the t-test value, the p -value of the t-test (H0: b = 0), and the R^2^ of the total model. Abbreviations are: GS = Guiana Shield, BS = Brazilian Shield, WAN = North-western Amazonia, WAS = South-western Amazonia, CA = Central Amazonia and EA = East Amazonia; TF = terra firme forests and PZ = white sand forests. Showing model coefficients (b) with their standard error (SE), t-test value, p -value of the t-test (H0: b = 0) and relative contribution to total R^2^; and total model R^2^.

| Model | | | Coefficient  estimate | Coefficient  SE | t-value | t-test  p-value | Rel. contr. R^2^ | R^2^ |
| --- | --- | --- | --- | --- | --- | --- | --- | --- |
| Carbon stock | | |  |  |  |  |  |  |
|  | Intercept (GS & TF) | | 160.76682 | 7.70549 | 20.864 | <2.00E-16 |  |  |
|  | Species richness | | 0.36773 | 0.05252 | 7.002 | 1.93E-11 | 0.03372553 |  |
|  | Biogeographical region | |  |  |  |  | 0.54889001 |  |
|  |  | BS | -98.00679 | 13.18136 | -7.435 | 1.33E-12 |  |  |
|  |  | WAN | -119.64254 | 10.0467 | -11.909 | <2.00E-16 |  |  |
|  |  | WAS | -97.00299 | 6.18059 | -15.695 | <2.00E-16 |  |  |
|  |  | CA | -102.37756 | 10.52598 | -9.726 | <2.00E-16 |  |  |
|  |  | EA | -52.08941 | 10.30015 | -5.057 | 7.78E-07 |  |  |
|  | Forest type | |  |  |  |  | 0.01710433 |  |
|  |  | PZ | 29.33733 | 9.04465 | 3.244 | 0.00133 |  |  |
|  |  |  |  |  |  |  |  | 0.5997 |

**Table S2.12**. Detailed results for the multivariate linear models of aboveground carbon stock predicted by woody species richness and either biogeographical region or forest types across the Amazonia dataset (n = 283). Showing model coefficients with its standard error (SE), the t-test value, the p -value of the t-test (H0: b = 0), and the R^2^ of the total model. Abbreviations are: GS = Guiana Shield, BS = Brazilian Shield, WAN = North-western Amazonia, WAS = South-western Amazonia, CA = Central Amazonia and EA = East Amazonia; TF = terra firme forests and PZ = white sand forests.

| Model | | | Coefficient  estimate | Coefficient  SE | t-value | t-test  p-value | Rel. contr. R^2^ | R^2^ |
| --- | --- | --- | --- | --- | --- | --- | --- | --- |
| Carbon stock | | |  |  |  |  |  |  |
|  | Species richness | | 0.28893 | 0.04736 | 6.101 | 3.55E-09 | 0.02805688 |  |
|  | Biogeographical region | |  |  |  |  | 0.55634896 |  |
|  |  | GS (intercept) | 174.65751 | 6.51541 | 26.807 | <2.00E-16 |  |  |
|  |  | BS | -104.44702 | 13.25382 | -7.881 | 7.57E-14 |  |  |
|  |  | WAN | -113.49069 | 10.03478 | -11.31 | <2.00E-16 |  |  |
|  |  | WAS | -100.29658 | 6.20088 | -16.175 | <2.00E-16 |  |  |
|  |  | CA | -96.79167 | 10.56174 | -9.164 | <2.00E-16 |  |  |
|  |  | EA | -54.54438 | 10.44798 | -5.221 | 3.51E-07 |  |  |
|  |  |  |  |  |  |  |  | 0.5844 |
| Carbon stock | | |  |  |  |  |  |  |
|  | Species richness | | 0.06036 | 0.06063 | 0.996 | 0.32033 | 0.001754594 |  |
|  | Forest type | |  |  |  |  | 0.024563278 |  |
|  |  | TF (Intercept) | 163.46102 | 9.78535 | 16.705 | <2.00E-16 |  |  |
|  |  | PZ | 36.24848 | 13.19202 | 2.748 | 0.00639 |  |  |
|  |  |  |  |  |  |  |  | 0.02632 |

**Table S2.13**. Detailed results for the bivariate linear models of aboveground carbon stock predicted by woody species richness, aggregated and separate per biogeographical region and per forest type, across Amazonia dataset (n = 283). Showing model coefficients with its standard error (SE), the t-test value, the p -value of the t-test (H0: b = 0), and the R^2^ of the total model.

| Model - subset | | | Coefficient | SE | t-value | p-value | R^2^ |
| --- | --- | --- | --- | --- | --- | --- | --- |
| Model AGC ~ Spp | | |  |  |  |  |  |
|  | Aggregated (n = 283) | |  |  |  |  |  |
|  |  | Intercept | 176.390492 | 8.679143 | 20.323 | <2e-16 |  |
|  |  | Species richness | -0.007433 | 0.056021 | -0.133 | 8.95E-01 |  |
|  |  |  |  |  |  |  | 6.264e-05 |
|  | GS (n = 165) | |  |  |  |  |  |
|  |  | Intercept | 164.22352 | 9.11304 | 18.021 | <2.00E-16 |  |
|  |  | Species richness | 0.37436 | 0.06875 | 5.445 | 1.87E-07 |  |
|  |  |  |  |  |  |  | 0.1539 |
|  | BS (n = 9) | |  |  |  |  |  |
|  |  | Intercept | 51.6467 | 15.7236 | 3.285 | 0.0134 |  |
|  |  | Species richness | 0.4853 | 0.1536 | 3.16 | 0.0159 |  |
|  |  |  |  |  |  |  | 0.5879 |
|  | WAN (n = 21) | |  |  |  |  |  |
|  |  | Intercept | 98.36182 | 9.52782 | 10.324 | 3.14E-09 |  |
|  |  | Species richness | 0.11902 | 0.04188 | 2.842 | 0.0104 |  |
|  |  |  |  |  |  |  | 0.2983 |
|  | WAS (n = 51) | |  |  |  |  |  |
|  |  | Intercept | 111.4714 | 10.75578 | 10.364 | 6.06E-14 |  |
|  |  | Species richness | 0.013 | 0.07668 | 0.169 | 0.866 |  |
|  |  |  |  |  |  |  | 0.0005858 |
|  | CA (n = 22) | |  |  |  |  |  |
|  |  | Intercept | 138.20344 | 34.51495 | 4.004 | 0.000697 |  |
|  |  | Species richness | 0.04483 | 0.13768 | 0.326 | 0.748104 |  |
|  |  |  |  |  |  |  | 0.7481 |
|  | EA (n = 15) | |  |  |  |  |  |
|  |  | Intercept | 47.2778 | 44.5883 | 1.06 | 0.3083 |  |
|  |  | Species richness | 0.7908 | 0.3022 | 2.616 | 0.0213 |  |
|  |  |  |  |  |  |  | 0.3449 |
|  | TF (n = 257) | |  |  |  |  |  |
|  |  | Intercept | 161.24536 | 9.87748 | 16.325 | <2e-16 |  |
|  |  | Species richness | 0.07515 | 0.06133 | 1.225 | 0.222 |  |
|  |  |  |  |  |  |  | 0.005853 |
|  | PZ (n = 26) | |  |  |  |  |  |
|  |  | Intercept | 227.1358 | 24.4714 | 9.282 | 2.06E-09 |  |
|  |  | Species richness | -0.3878 | 0.3499 | -1.108 | 0.279 |  |
|  |  |  |  |  |  |  | 0.0487 |


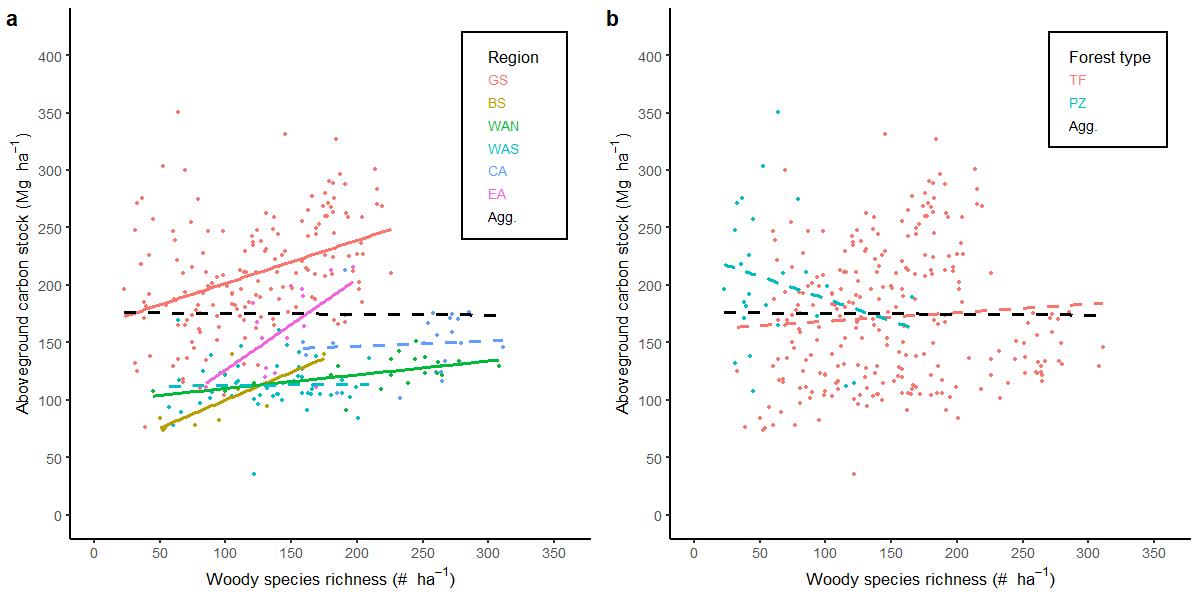


**Figure S2.5.** Visualisation of linear bivariate relationships between ecosystem services and species richness for different biogeographical regions (Panel a), forest types (Panel b) and aggregated (“Agg.”, black line) across 283 1-ha Amazonian lowland tropical forest plots. Solid lines relationship p < 0.05 and dashed lines relationship p ≥ 0.05. Abbreviations for regions: GS = Guiana Shield (n = 165), BS = Brazilian Shield (n = 9), WAN = North-western Amazonia (n = 21), WAS = South-western Amazonia (n = 51), CA = Central Amazonia (n = 22) and EA = East Amazonia (n = 15); for forest types: TF = terra firme forests (n = 257) and PZ = white sand forests (n = 26). Model details included in Table S2.13

**Table S2.14**. Analysis of variance F-tests for aboveground carbon stock and woody species richness across biogeographical regions and across forest types in the Amazonia dataset (6 regions, 5 forest types, n = 283).

| **Model** | **F-statistic** | **Df1** | **Df2** | **p-value** | **Multiple R-squared** |
| --- | --- | --- | --- | --- | --- |
| Carbon stock ~ Region | 62.06 | 5 | 277 | < 2.2e-16 | 0.5284 |
| Species richness ~ Region | 38.16 | 5 | 277 | < 2.2e-16 | 0.4078 |
| Carbon stock ~ Forest type | 6.577 | 1 | 281 | 0.01085 | 0.02287 |
| Species richness ~ Forest type | 55.77 | 1 | 281 | 1.032e-12 | 0.1656 |

**Table S2.15**. Summary of post-hoc Tukey contrasts of aboveground carbon stock and woody species richness across biogeographical regions and across forest types in the Amazonia dataset (6 regions, 5 forest types, n = 283). Showing adjusted p values of single-step method.

| **Linear Hypothesis** | | **Estimate** | **SE** | **t-value** | **Adjusted p-value** |
| --- | --- | --- | --- | --- | --- |
| Carbon stock ~ Region | |  |  |  |  |
|  | H0: BS - GS == 0 | -112.415 | 14.025 | -8.015 | <0.001 |
|  | H0: WAN - GS == 0 | -85.53 | 9.493 | -9.01 | <0.001 |
|  | H0: WAS - GS == 0 | -9.67E+01 | 6.564 | -14.735 | <0.001 |
|  | H0: CA - GS == 0 | -60.661 | 9.3 | -6.523 | <0.001 |
|  | H0: EA - GS == 0 | -4.79E+01 | 11.05 | -4.335 | <0.001 |
|  | H0: WAN - BS == 0 | 2.69E+01 | 16.324 | 1.647 | 0.54161 |
|  | H0: WAS - BS == 0 | 1.57E+01 | 14.814 | 1.059 | 0.88549 |
|  | H0: CA - BS == 0 | 51.754 | 16.212 | 3.192 | 0.01739 |
|  | H0: EA - BS == 0 | 64.516 | 17.276 | 3.734 | 0.00269 |
|  | H0: WAS - WAN == 0 | -11.196 | 10.624 | -1.054 | 0.88759 |
|  | H0: CA - WAN == 0 | 24.869 | 12.5 | 1.99 | 0.32673 |
|  | H0: EA - WAN == 0 | 37.631 | 13.851 | 2.717 | 0.06731 |
|  | H0: CA - WAS == 0 | 36.065 | 10.451 | 3.451 | 0.00742 |
|  | H0: EA - WAS == 0 | 48.827 | 12.035 | 4.057 | <0.001 |
|  | H0: EA - CA == 0 | 12.762 | 13.72 | 0.93 | 0.93085 |
| Species richness ~ Region | |  |  |  |  |
|  | H0: BS - GS == 0 | -27.578 | 16.734 | -1.648 | 0.541 |
|  | H0: WAN - GS == 0 | 96.771 | 11.326 | 8.544 | <0.001 |
|  | H0: WAS - GS == 0 | 12.357 | 7.832 | 1.578 | 0.588 |
|  | H0: CA - GS == 0 | 125.048 | 11.096 | 11.27 | <0.001 |
|  | H0: EA - GS == 0 | 23 | 13.184 | 1.745 | 0.477 |
|  | H0: WAN - BS == 0 | 124.349 | 19.477 | 6.384 | <0.001 |
|  | H0: WAS - BS == 0 | 39.935 | 17.675 | 2.259 | 0.196 |
|  | H0: CA - BS == 0 | 152.626 | 19.344 | 7.89 | <0.001 |
|  | H0: EA - BS == 0 | 50.578 | 20.612 | 2.454 | 0.129 |
|  | H0: WAS - WAN == 0 | -84.415 | 12.675 | -6.66 | <0.001 |
|  | H0: CA - WAN == 0 | 28.277 | 14.914 | 1.896 | 0.381 |
|  | H0: EA - WAN == 0 | -73.771 | 16.527 | -4.464 | <0.001 |
|  | H0: CA - WAS == 0 | 112.692 | 12.47 | 9.037 | <0.001 |
|  | H0: EA - WAS == 0 | 10.643 | 14.359 | 0.741 | 0.973 |
|  | H0: EA - CA == 0 | -102.048 | 16.369 | -6.234 | <0.001 |
| Carbon stock ~ Forest type | |  |  |  |  |
|  | H0: PZ - TF == 0 | 30.9 | 12.05 | 2.565 | 0.0108 |
| Species richness ~ Forest type | |  |  |  |  |
|  | H0: PZ - TF == 0 | -88.55 | 11.86 | -7.468 | 1.03E-12 |

**
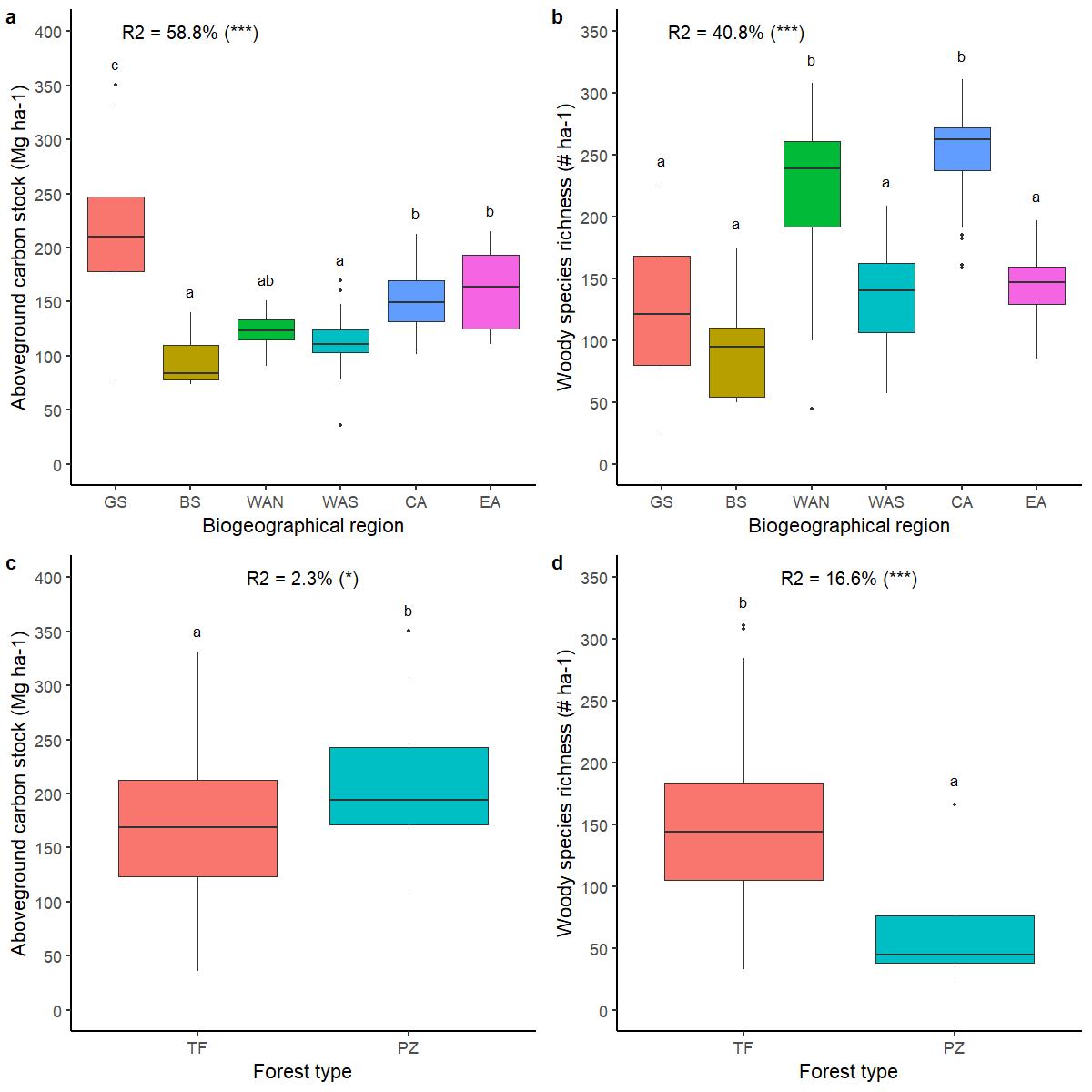
**

**Figure S2.6.** Box plots of aboveground carbon stock and woody species richness values per biogeographical region (Panels a and b) and per forest type (Panels c and d) across the Amazonia dataset (n = 283). R2 is given, and the p-value rank of the F-test is given between parentheses. P-value ranks: p < 0.001 (***), p < 0.01 (**), p < 0.05 (*), p ≥ 0.05 (ns). Significance differences between the means on the basis of Post-hoc Tukey Contrasts are indicated by unique letter combinations. Abbreviations are: GS = Guiana Shield (n = 165), BS = Brazilian Shield (n = 9), WAN = North-western Amazonia (n = 21), WAS = South-western Amazonia (n = 51), CA = Central Amazonia (n = 22) and EA = East Amazonia (n = 15); for forest types: TF = terra firme forests (n = 257) and PZ = white sand forests (n = 26). F-test values and Tukey contrasts can be found in Table S2.14-S2.15.
